# Longitudinal immune profiling following autologous hematopoietic stem cell transplantation in multiple sclerosis: insights into immune reconstitution and disease modulation

**DOI:** 10.3389/fimmu.2025.1601223

**Published:** 2025-06-30

**Authors:** Malin Müller, Ivan Pavlovic, Anna Wiberg, Joachim Burman

**Affiliations:** ^1^ Department of Medical Sciences, Uppsala University, Uppsala, Sweden; ^2^ Department of Immunology, Genetics and Pathology, Uppsala University, Uppsala, Sweden

**Keywords:** multiple sclerosis (MS), autologous hematopoietic stem cell transplantation (AHSCT), neuroimmunology, mass cytometry, immune reconstitution, flow cytometry

## Abstract

**Introduction:**

Autologous hematopoietic stem cell transplantation (AHSCT) is an effective treatment for relapsing remitting multiple sclerosis, yet the mechanisms underlying immune reset and sustained remission remain incompletely understood. This study provides a longitudinal immune profiling of patients undergoing AHSCT, with a specific focus on immune reconstitution at two years post-AHSCT.

**Methods:**

Peripheral blood mononuclear cells (PBMCs) were collected from 22 relapsing-remitting multiple sclerosis patients at baseline and multiple time points post-AHSCT. Immune reconstitution was characterized using high-dimensional mass cytometry (CyTOF) and flow cytometry to assess phenotypic changes in B cells, T cells, and myeloid cells.

**Results:**

AHSCT led to profound alterations in immune cell populations. B-cell recovery was marked by a rapid expansion of naïve B cells, while memory B cells and plasmablasts remained depleted. Notably, patients with evidence of inflammatory disease activity (EIDA) post-AHSCT exhibited higher pre-transplant frequencies of non-switched IgD^+^IgM^+^ memory B cells, raising the possibility of a potential biomarker for treatment response. Myeloid-cell reconstitution showed a decline in classical monocytes and an increase in non-classical monocytes and plasmacytoid dendritic cells, potentially shifting the immune balance toward a more tolerogenic state. CD4 T-cell reconstitution demonstrated a shift from central memory (T_cm_) to effector memory (T_em_) phenotypes, with a selective depletion of polyfunctional Th1/Th17cells lacking PD-1 expression. Clusters enriched for PD-1^+^ T_em_ CD4 T cells appeared to differ between patients with and without EIDA. Furthermore, an increase in atypical naïve CCR7^⁻^CD62L^⁻^ CD4 T cells was observed in EIDA patients, raising questions about their role in the pathophysiology of MS. CD8 T-cell reconstitution followed a similar pattern, with a shift from a naïve/T_cm_-dominant to a T_em_-skewed population, albeit with substantial interpatient variability. Mucosal-associated invariant T cells (MAIT) cells showed a sustained decrease, possibly reflecting microbiota alterations post-transplant.

**Conclusions:**

Taken together, these findings provide an exploratory characterization of immune reconstitution following AHSCT, highlighting candidate biomarkers and mechanisms that warrant validation in larger cohorts to guide patient stratification and monitor treatment responses in multiple sclerosis.

## 1 Introduction

Multiple sclerosis (MS) is a chronic autoimmune disorder of the central nervous system (CNS) characterized by inflammation, demyelination, and progressive neuronal damage. It remains one of the most common causes of non-traumatic disability in young adults, with substantial individual and societal burdens (1). While the precise etiology of MS is not fully understood, it is widely accepted that an interplay of genetic, environmental, and immunological factors triggers the disease. This results in autoreactive T and B lymphocytes attacking myelin and other CNS components, leading to MS lesions and neurodegeneration (1).

Autologous hematopoietic stem cell transplantation (AHSCT) has emerged as a transformative treatment for aggressive and treatment-refractory forms of relapsing-remitting MS (RRMS). The procedure involves immune ablation using high-dose chemotherapy, followed by reinfusion of autologous hematopoietic stem cells. This dual approach achieves comprehensive immune resetting, eliminating autoreactive immune cells and facilitating the reconstitution of a less autoreactive immune repertoire (2). AHSCT has demonstrated high efficacy in achieving sustained remission, with up to 80% of treated patients showing long-term freedom from relapses, new MRI lesions, and disability progression (3).

The mechanisms underlying the efficacy of AHSCT are multifaceted. Immune ablation eliminates autoreactive T and B cells, while immune reconstitution, mediated by thymic rebound, promotes the generation of naive T cells and a diversified T-cell receptor (TCR) repertoire. This process is critical for restoring immune tolerance and reducing CNS inflammation (4). AHSCT also modulates the cytokine milieu, reducing levels of pro-inflammatory mediators such as IL-17, while increasing anti-inflammatory cytokines like IL-10, fostering an environment conducive to long-term immune homeostasis (5, 6).

On a cellular level, the therapy profoundly alters the composition of immune cell subsets. Regulatory T cells (T_regs_) and CD56^high^ natural killer (NK) cells expand early post-transplantation, enhancing immunoregulatory functions, while pathogenic memory B cells and plasmablasts are significantly depleted. These shifts mitigate the pathogenic immune responses characteristic of MS (7–9). Moreover, the diversification of Epstein-Barr virus (EBV)-specific cytotoxic T-cell responses following AHSCT provides insights into the interplay between viral immunity and autoimmune pathogenesis (10). However, no clear association between increased immune regulatory functions, decrease in specific pathogenic immune cells and an event free outcome have been found.

Despite the overall promise of the treatment, challenges persist, including treatment-related toxicity, incomplete immune reset in some patients, and the risk of secondary autoimmunity. Refining conditioning regimens to minimize toxicity while maintaining efficacy remains a priority for optimizing AHSCT outcomes.

This study investigates the immunological changes following AHSCT in 22 patients with RRMS, focusing particularly on T- and B-cell reconstitution and associated phenotypic shifts apparent at two years post-AHSCT. We selected the two-year post-AHSCT time point as our primary analytical focus as immune reconstitution is expected to be largely complete at this stage (11). To provide additional depth and context, we supplemented this analysis with all other available samples obtained at various irregular intervals. Using mass cytometry and flow cytometry, we comprehensively profiled peripheral blood mononuclear cells (PBMCs) at baseline and at multiple time points post-transplantation. By characterizing these immunological shifts, we aimed to elucidate the mechanisms underlying the long-term efficacy of AHSCT in MS and identify potential biomarkers predictive of clinical outcomes.

## 2 Methods

### 2.1 Participants

Patients with relapsing-remitting multiple sclerosis (RRMS), diagnosed according to the 2017 revised McDonald criteria (12), who underwent AHSCT with a cyclophosphamide and anti-thymocyte globulin (ATG) conditioning regimen at Uppsala University Hospital between October 2011 and June 2022, were invited to participate. Blood samples were collected from 22 patients scheduled for AHSCT, at baseline (pre-AHSCT) (n=20) and at various follow-up intervals post-AHSCT. Summary demographics of study subjects (**Table 1**), as well as detailed demographics, sampling time points and performed analysis are provided in **Supplementary Table S1**.

**Table 1:** The table contains a summary of the demographic and clinical data of multiple sclerosis (MS) patients who underwent hematopoietic stem cell transplantation (HSCT), as well as the ages of healthy control (HC) subjects.

Characteristics	MS, AHS CT (n=22)	Healthy controls (n=17)	MS, Newly diagnosed (n=22)
Age at inclusion, Median [range]	30 [22-48]	35 [23-62]	No info
Sex, F/M, (%women)	14/8 (64)	12/5 (71)	No info
Disease duration (years), Median [range]	1.75 [0.1-16.7]	n/a	No info
EDSS, baseline, Median [range]	3.5 [2.0-7.0]	n/a	No info
Number of previous treatments, Median [range]	2 [0-5]	n/a	No info
Annual relapse rate, Median [range]	0 [0-1]	n/a	No info
New MRI leasions rate, Median [range]	0 [0-1]	n/a	No info
Disease activity, NEDA/EIDA (%NEDA)	18/4 (82)	n/a	No info

Patient characteristics for the control group of newly diagnosed (ND) MS patients were blinded and therefore inaccessible. n/a; not applicable.

Healthy controls (HC) and newly diagnosed MS patients (ND) served as comparison groups. Pseudonymized HC samples were obtained from blood donors matched by sex and age, whereas ND samples were derived from anonymized biobank material, thus precluding access to detailed clinical and demographic data. Consequently, demographic and clinical characteristics for ND patients are not reported in **Table 1**.

Four patients showed potential evidence of disease activity post-AHSCT: three experienced clear clinical relapses, new T2 lesions, and gadolinium-enhancing lesions on MRI, indicating definite inflammatory disease activity. One additional patient had a single new small T2 lesion detected on MRI without associated clinical symptoms or gadolinium enhancement; thus, it is uncertain whether this represents true inflammatory disease activity or an incidental finding. This patient was included in descriptive analyses but is highlighted separately (with a distinct color in figures) to indicate this uncertainty. Due to the small number of patients with disease activity (three definitive, one uncertain), formal statistical comparisons between patients with and without disease activity post-AHSCT were not performed.

### 2.2 Peripheral blood mononuclear cell isolation

Peripheral blood mononuclear cells (PBMCs) were isolated using Ficoll-Paque PLUS density gradient centrifugation (Cytiva). Isolated cells were cryopreserved in fetal calf serum supplemented with 10% dimethyl sulfoxide (DMSO) and stored at -170°C.

### 2.3 Absolute cell counts of CD4 and CD8 T cells in whole blood (flow cytometry)

Blood samples were drawn at the clinic and T cell count were performed as part of the clinical immune reconstitution follow up. For all these timepoints were not phenotype analysis performed. For detailed information about patients and time points for these samples see **Supplementary Table S1**. A volume of 50µl blood, acquired in EDTA tubes, were pipetted in to Trucount™ tubes (BD Bioscience) containing a known number of fluorescent beads and stained with antibodies for CD3-FITC, CD45-Per Cp5.5, CD4-PeCy7 and CD8-APCCy7 incubated in the dark for 15 minutes at room temperature (RT). The erythrocytes were lysed using BD FACS™ lysing solution, diluted 1:10, and the sample was incubated for another 15 min in the dark at RT.

Cells were analyzed by flow cytometry on a DxFLEX instrument (Beckman Coulter), CD45^+^ leukocytes were gated on CD3^+^CD4^+^ and CD3^+^CD8^+^ and frequencies of these cells were established. CD4 and CD8 T cells were enumerated in relation to the fixed number of fluorescent beads in each sample.

### 2.4 Mass cytometry (CyTOF)

Cryopreserved PBMCs were analyzed using mass cytometry at the CryoSciLifeLab Cellular Immunomonitoring Facility in Stockholm, Sweden. Analysis included B/Myeloid, T-cell and intracellular panels (**Supplementary Figures 1**, **S1.1, S1.2**). Thawed cells were incubated in Benzonase-supplemented media and allowed to recover for 2 hours at 37°C and 5% CO_2_ before staining.

#### 2.4.1 Barcoding (CyTOF)

Cells were barcoded using the Cell-ID 20-Plex Pd Barcoding Kit (Fluidigm). After barcoding, samples were pooled, washed, and stained with surface antigen antibodies (**Supplementary Figures 1**; **S1.1, S1.2**). Non-fixated samples were stored in 2% formaldehyde, while samples for intracellular staining underwent fixation and permeabilization using eBio Fixation/Permeabilization buffers.

#### 2.4.2 Intracellular antigen staining (CyTOF)

Fixed and permeabilized cells were incubated with antibodies against CTLA-4 and Ki67 for 45 minutes. DNA staining with intercalator Iridium (Fluidigm) was performed before acquisition on a Helios mass cytometer (Fluidigm). Signal normalization was achieved using equilibration beads.

### 2.5 Flow cytometry

Upon analysis, frozen PBMCs were thawed at 37°C and washed twice in pre-warmed (37°C) RPMI-1640 medium supplemented with 10% heat-inactivated fetal calf serum, 10 mM HEPES, and 2 mM L-glutamine (all from Gibco). Cell viability and counts were assessed, and 1 × 10^6^ cells per sample were resuspended in calcium- and magnesium-free phosphate-buffered saline (PBS) containing 5% normal mouse serum to block nonspecific binding.

#### 2.5.1 Cell surface staining (flow cytometry)

Cells were incubated with pre-prepared antibody cocktails (**Supplementary Table S2**) for 30 minutes on ice in the dark, washed twice in PBS, and resuspended in PBS supplemented with ethylenediaminetetraacetic acid (EDTA). For viability assessment, 7-AAD was added 10 minutes before acquisition. Flow cytometry was performed using a FACSVerse (BD Biosciences).

#### 2.5.2 Nuclear antigen staining for Helios and FoxP3 (flow cytometry)

Surface-stained PBMCs were incubated with fixable viability dye for 30 minutes on ice in the dark, followed by fixation and permeabilization with the True-Nuclear™ Transcription Buffer Set (BioLegend). Cells were then blocked with 5% normal mouse serum and stained intracellularly for Helios and FoxP3. Samples were washed, resuspended in PBS-EDTA, and stored for acquisition.

### 2.6 Data processing and analysis

Mass cytometry data were normalized and de-barcoded. Files were analyzed using FlowJo (versions 10.6.2 and 10.8.1) for gating and phenotypic assessments. Briefly, cells of all CyTOF FCS files from panel 1 and panel 2 were subjected to initial clean up gating in FlowJo (**Supplementary Figures 1**; **S1.3**) and FCS files containing CD45^+^ leukocytes together with relevant channels were exported and imported into new FlowJo worksheets. Equal numbers of cells per subset (e.g., 40,000 CD45^+^ cells, 20,000 T cells) were randomly selected (down sampled) and files concatenated for downstream analysis. The resulting mass cytometry data were processed in FlowJo using high-dimensional reduction algorithms (UMAP or t-SNE), followed by cell subset clustering with the graph-based clustering algorithm PhenoGraph clustering tool. The phenotype of each identified cell cluster was further characterized using Cluster Explorer, which enabled the visualization and annotation of clusters based on marker expression profiles. The Myeloid cell population was defined as CD3^-^CD19^-^CD20^-^HLA-DR^+^ and NK cells CD3^-^CD19^-^CD20^-^HLA-DR^neg/low^. For gaiting strategy of NK and Myeloid cells and marker inclusions for Myeloid cell subsets see **Supplementary Figures 1**; **S1.4, S1.5**. Expression patterns of markers used in each immune cell subset analysis are outlined in **Supplementary Figures 1**; **S1.6-S1.8**.

Flow cytometric data was performed in FlowJo (versions 10.6.2 and 10.8.1). Initial gating strategy for phenotype analysis of live PBMCs (panel 1-4) (**Supplementary Figures 1**; **S1.9**) and for fixated permeabilized PBMCs analyzing nuclear expressed antigens (**Supplementary Figures 1**; **SS1.10**). Gating strategies for each flow cytometric panel (panel 1-5) (**Supplementary Figures 1**; **S1.11-S1.15**). Definitions of CD4 T helper subsets are presented in **Supplementary Figures 1**, **S1.16**.

### 2.7 Statistical analysis

Data are presented as medians with minimum and maximum values. The Wilcoxon matched-pairs signed-rank test was used for paired baseline and post-AHSCT comparisons. Differences between unpaired groups were assessed using the Mann-Whitney U test. For multiple group comparisons, Kruskal-Wallis tests followed by Dunn’s *post hoc* tests were applied. Statistical significance was defined as p < 0.05. Analyses were performed using GraphPad Prism (version 10).

### 2.8 Ethical considerations

The study was approved by the Regional Ethical Review Board in Uppsala (Dnr 2010/450/1 and 2012/080/1). The study was performed in concordance with the Declaration of Helsinki (1964), and all patients provided written informed consent.

## 3 Results

### 3.1 CD4 and CD8 T cell recovery

**Figure 1:**
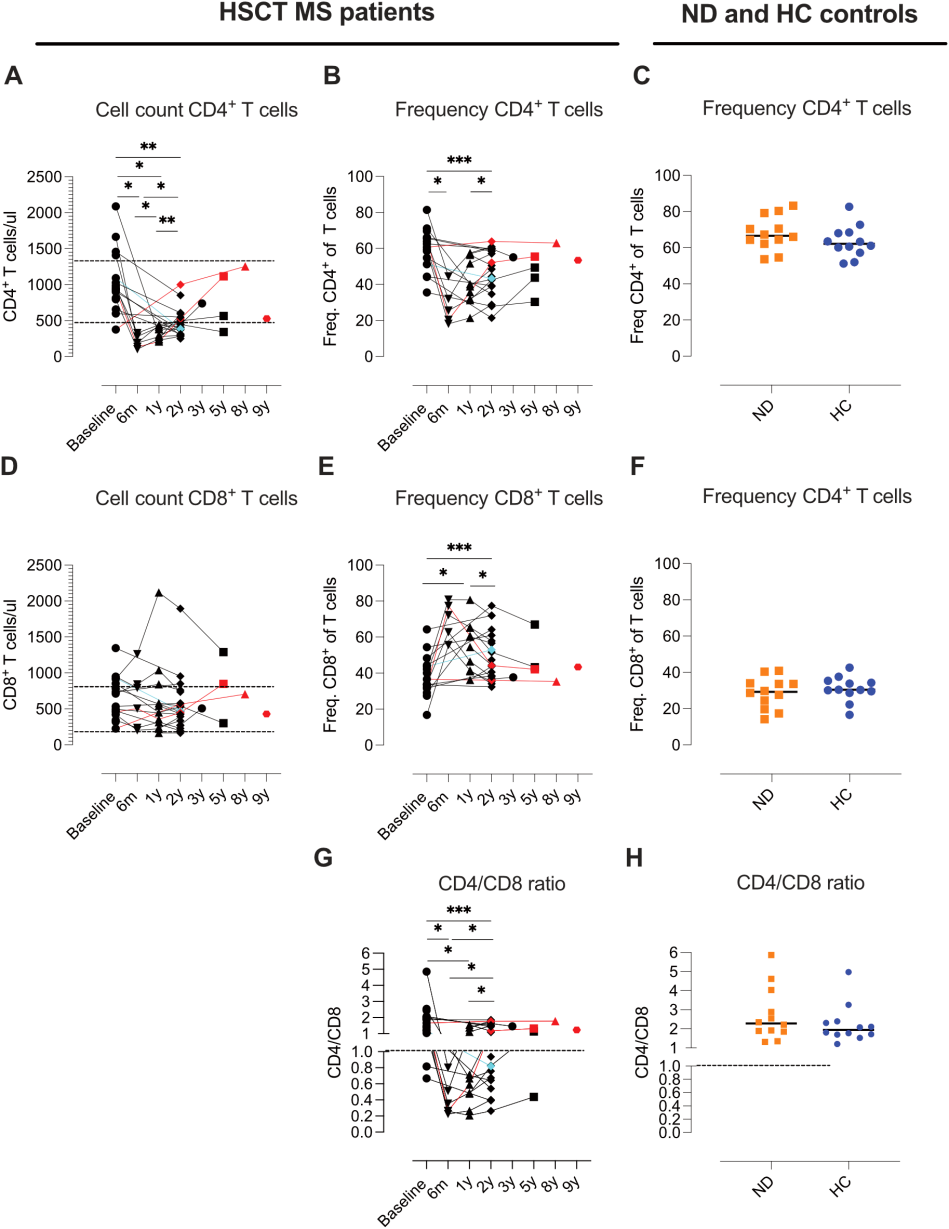
Dynamics of CD4 and CD8 T-cell reconstitution in MS patients following AHSCT. Absolute CD3, CD4 and CD8 T cell counts in AHSCT treated patients were conducted in whole blood by flow cytometry and frequencies of CD4 and CD8 T cells were calculated out of total amount of CD3 T cells. Absolute CD4 T cell counts **(A)** and CD8 T cell counts **(D)**. Frequencies of CD4 T cells **(B)** and CD8 T cells **(C)**, as well as CD4/CD8 ratios **(G)**. Time points and sample analyzed for AHSCT treated patients are as follows, baseline (n=16) and post-AHSCT: 6 months (n=6), 1 year (n=11), 2 years (n=21), 3.5 years (n=1), 5 years (n=3), 8 years (n=1), and 9 years (n=1). For reference, frequencies of CD4 and CD8 T cells and CD4/CD8 ratios in newly diagnosed (ND) MS patients (n=12) and healthy controls (HC) (n=12) **(C, F, H)**. The horizontal lines in panels **(A, D)** indicate the upper and lower limits of the normal reference range for T-cell subset counts in blood (CD4: 490–1340 cells/µl; CD8: 190–800 cells/µl). In panels **(G, H)**, a CD4/CD8 ratio of 1 is marked as the threshold for normality. Patients with a relapse post-AHSCT are highlighted in red, while one patient with a new T2 lesion post-AHSCT is marked in turquoise. Statistical analyses were performed with the Wilcoxon matched-pair test to compare paired samples at each time point (*p<0.05, **p<0.01, ***p<0.001).

Whole blood collected at baseline, 6 months, 1 year, 2 years as well as a few later timepoints were analyzed for absolute counts of CD4 and CD8 T cells by flow cytometry. CD4 T cells demonstrated significantly reduced absolute counts at 6 months, 1 year, and 2 years post-AHSCT compared to baseline, with gradual recovery observed over time. However, for most patients CD4 T cell counts and frequencies remained below baseline throughout the follow-up period (**Figures 1A, B**). In contrast, CD8 T cell counts remained within normal ranges, with no significant changes (**Figure 1D**).

The CD4/CD8 ratio was inverted in all patients at 6 months post AHSCT but showed partial normalization in six patients by two years (**Figures 1G, H**). Patients with EIDA had all regained normal CD4 counts and CD4/CD8 ratios during the observation period. However, this finding was not exclusive for relapsed patients.

### 3.2 Mass cytometric analysis of PBMC at baseline and post-AHSCT

Cryopreserved PBMC samples from 12 of the 22 MS patients treated with AHSCT, collected at baseline and two years post-treatment, were selected for immunophenotypic analysis using CyTOF panel 1. Samples from 10 newly diagnosed, untreated MS patients (ND) and 8 healthy controls (HC) were included as reference groups. Two antibody panels were utilized: panel 1 (26 markers), designed to capture an overall leukocyte phenotype with a focus on B cells and myeloid cells (**Supplementary Figures 1**, **S1.1**), and panel 2 (24 markers), tailored for an in-depth analysis of T cell phenotypes (**Supplementary Figures 1**; **S1.2**).

Mass cytometry data were processed in FlowJo, where high-dimensional reduction algorithms (UMAP or t-SNE) were applied to visualize cellular distributions. Cell subsets were then identified using the PhenoGraph clustering algorithm. The phenotype of each identified cluster was further characterized using Cluster Explorer, which facilitated the visualization and annotation of clusters based on marker expression profiles.

### 3.3 Decreased myeloid cell and elevated B-cell proportions post-AHSCT

**Figure 2:**
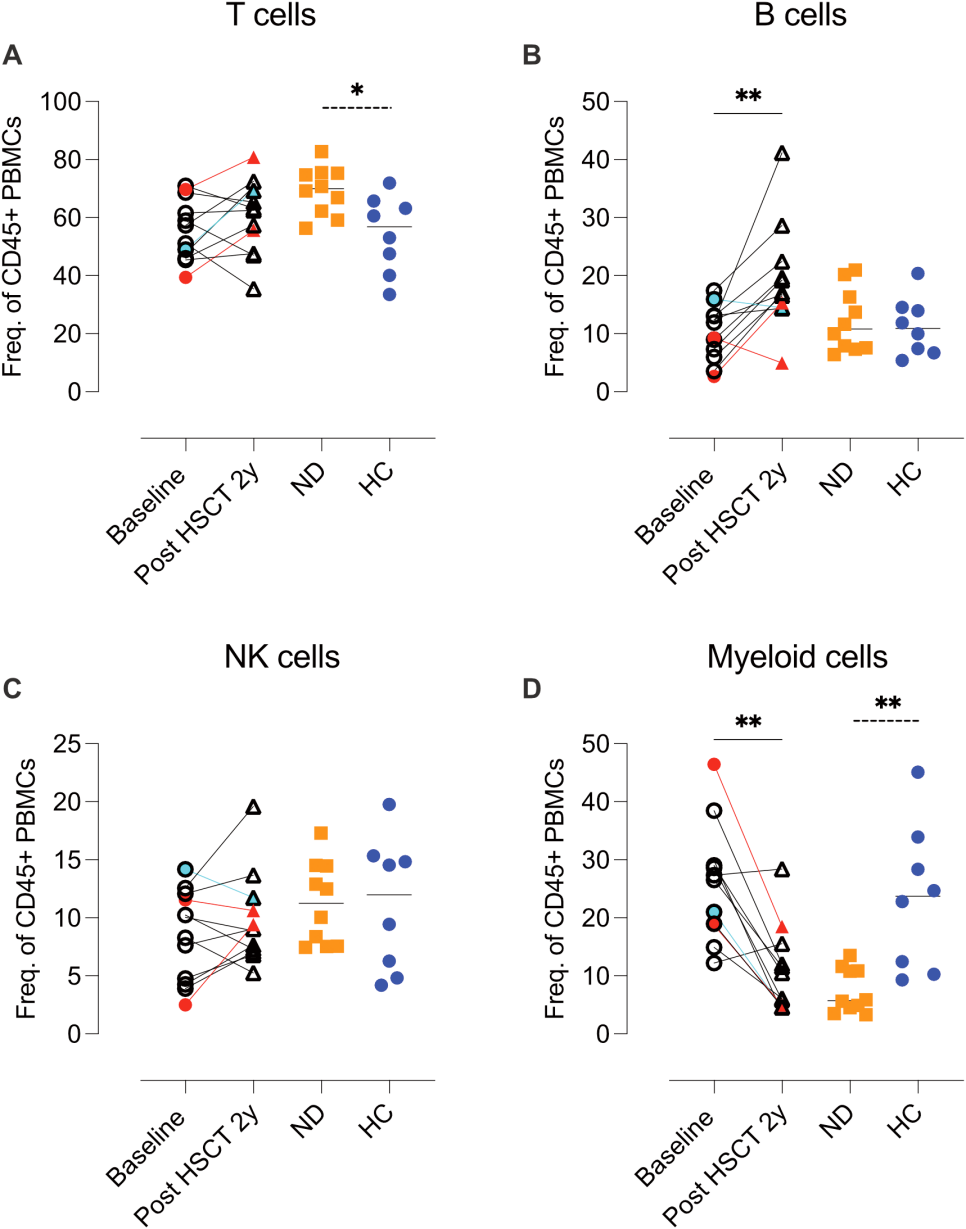
Comparative analysis of frequencies of T, B, NK and myeloid cells in MS patients at baseline and two years post-HSCT. Analysis of mass cytometric data (CyTOF, panel 1) of cell composition of the CD45^+^ leukocyte population. Summary graphs show the frequencies of T cells **(A)**, B cells **(B)**, NK cells **(C)**, and myeloid cells **(D)** in each subject at baseline and two years post-AHSCT (n=12). Additionally, data from newly diagnosed MS patients (ND) (n=10) and healthy controls (HC) (n=8) are included for comparison. The cell populations were manually gated and defined as follows: T cells CD3^+^CD19^-^CD14^-^; B cells CD3^-^CD19^+^CD20^+^/^-^; NK cells CD3^-^CD19^-^CD14^-^HLA-DR^-/dim^; Myeloid cells CD3^-^CD19^-^CD20^-^HLA-DR^+/high^. Frequencies were calculated as a proportion of total CD45^+^ leukocytes. Patients with a relapse post-AHSCT are highlighted in red, while one patient with a new T2 lesion post-AHSCT is marked in turquoise. Statistical analyses were performed using the Wilcoxon matched-pair test (solid line, **p<0.01) to compare paired samples and the Mann-Whitney test (hatched line, *p<0.05, **p<0.01) to compare unpaired groups.

Analysis of CyTOF data from the B-cell/myeloid panel (CyTOF, panel 1) revealed an increase in B-cell frequencies and a concurrent decrease in myeloid cell proportions two years post-AHSCT. In contrast, T- and NK-cell proportions remained stable (**Figures 2A–D**, **Supplementary Figures 2**, **S2.1**). The ND group exhibited significantly higher T-cell frequencies and lower myeloid cell proportions compared to HC (**Figures 2A, D**).

### 3.4 Reductions in classical monocytes post-AHSCT

**Figure 3:**
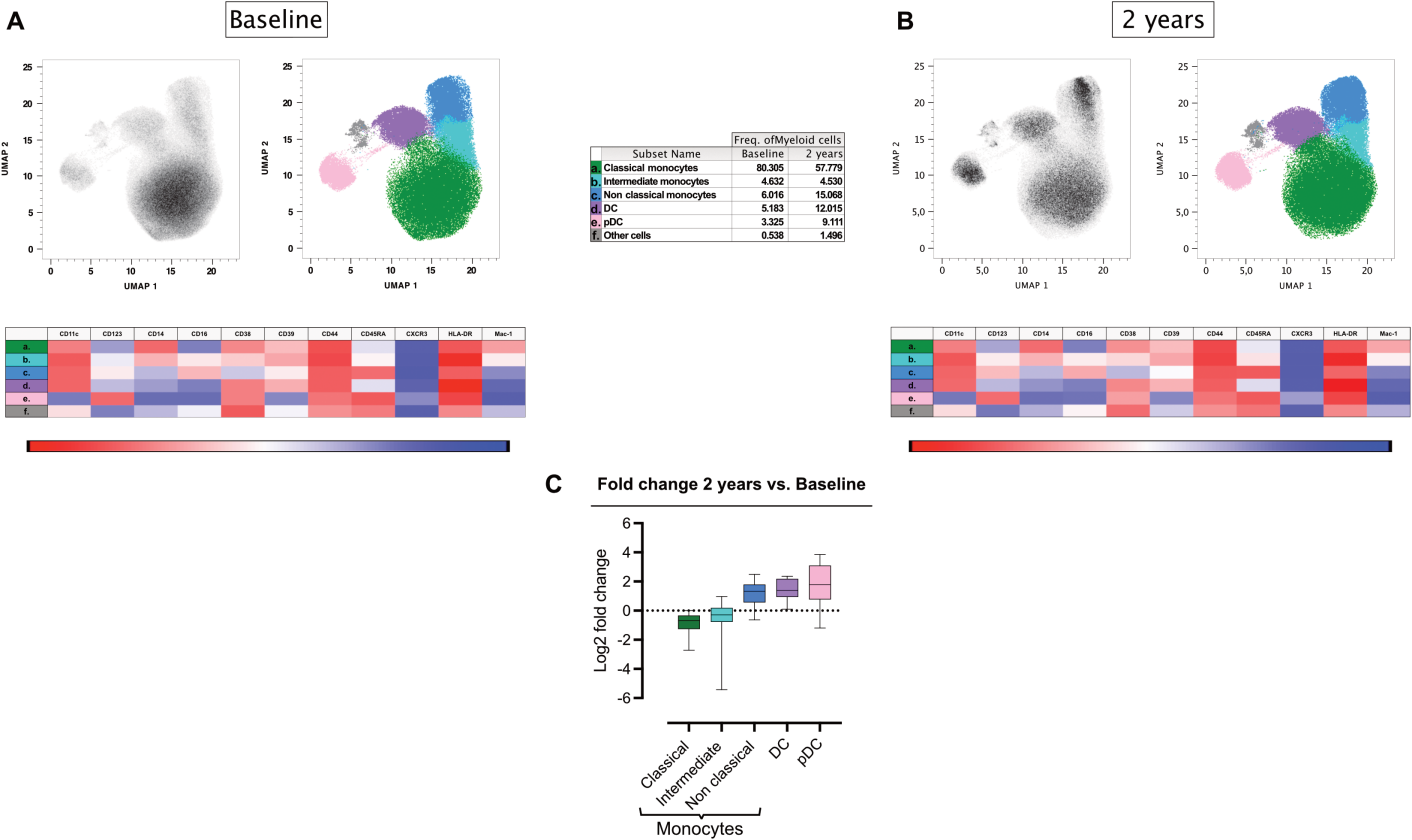
Differential frequencies of myeloid cell subsets in MS at two years post-AHSCT. Analysis of mass cytometric data (CyTOF, panel 1) of the myeloid cell population (CD45^+^CD3^-^CD19^-^CD20^-^HLA-DR^+/high^) in PBMCs from MS patients at baseline (n=12) and two years post-AHSCT (n=12). The CyTOF data were concatenated, myeloid cells were manually gated and subjected to high-dimensional reduction analysis of the myeloid cell population (CD3^-^CD19^-^CD20^-^HLA-DR^+/high^) using UMAP, followed by Phenograph clustering and phenotypic analysis of cell clusters with Cluster Explorer. Panels show the distribution of myeloid cell clusters at baseline **(A)** and two years post-AHSCT **(B)**, alongside a table summarizing the mean relative frequencies of each cell cluster at both time points. The heatmaps show expression levels of cellular markers in each cell cluster. The summary graph **(C)** depicting log_2_ fold changes in the frequencies of phenotypically distinct myeloid cell clusters at two years post-AHSCT compared to baseline across all transplanted patients. Dispersion measures are represented by the median, along with the minimum and maximum log_2_ fold change values.

A more detailed assessment of HLA-DR^+^ myeloid subsets (CD3^-^CD20^-^CD19^-^) showed a reduction in classical monocytes (CD14^+^CD16^-^) (p<0.001) and an increase in non-classical monocytes (CD14^-^CD16^+^) (p<0.01) following AHSCT (CyTOF, panel 1). Intermediate monocytes (CD14^int^CD16^low^) remained unchanged (**Figures 3A–C**, **Supplementary Figures 2**; **S2.2A-C**). Furthermore, the relative frequencies of plasmacytoid dendritic cells (pDCs) and conventional dendritic cells (DCs) were elevated two years post-AHSCT (p<0.01 and p<0.001 respectively) (**Figures 3A–C**, **Supplementary Figures 2**; **S2.2D, E**). Intriguingly, the composition of the myeloid compartment in post-AHSCT patients resembled the ND group with lower frequencies of classical monocytes and a rise in pDCs and DCs (**Supplementary Figures 2**, **S2.2A, D, E**). Two of the three patients with EIDA (one experiencing a clinical relapse and another with a new T2 lesion) exhibited the lowest classical monocyte frequencies and the highest pDC frequencies among transplanted patients (**Supplementary Figures 2**; **S2.2A, D**).

### 3.5 Higher frequencies of non-switched memory B cells at baseline in relapsed patients post-AHSCT

**Figure 4:**
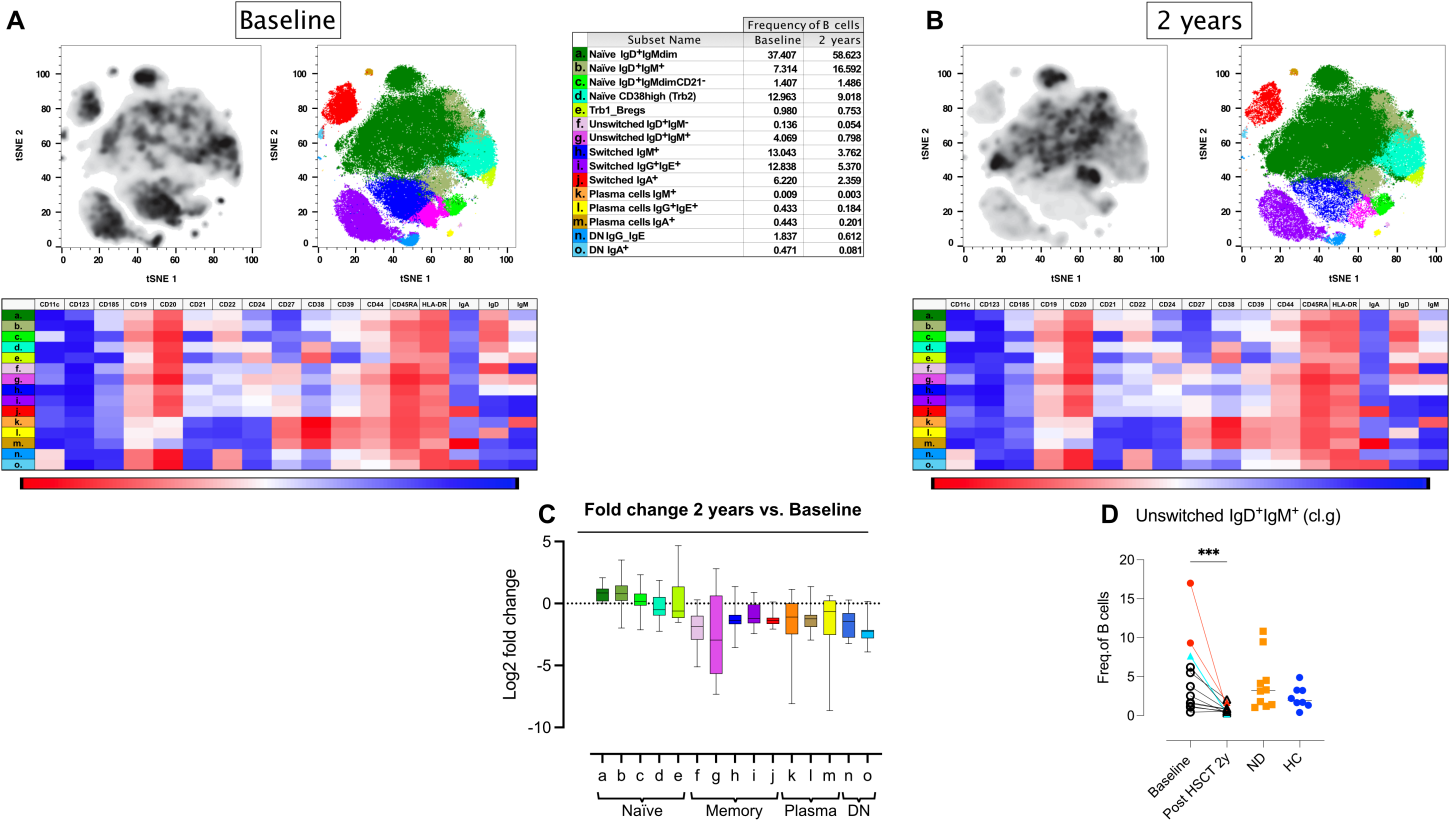
Alterations of naïve and memory B cells in MS patients following AHSCT. Analysis of mass cytometric data (CyTOF, panel 1) of the B cell population (CD45^+^CD3^-^CD19^+^CD20^+^/^-^) in PBMCs from MS patients at baseline (n=12) and two years post-AHSCT (n=12). The CyTOF data were concatenated, B cells were manually gated and subjected to high-dimensional reduction analysis of the B cell-population using t-SNE, followed by Phenograph clustering and heatmap analysis of marker expression within distinct cell clusters, visualized using Cluster Explorer. The distribution of B-cell clusters is shown at baseline **(A)** and two years post-AHSCT **(B)**, along with a table summarizing the mean relative frequencies of each cluster at both time points. The heatmaps show mean expression levels of cellular markers in each cell cluster. The summary graph **(C)** depicting the log_2_ fold changes in the frequencies of phenotypically distinct B cell clusters at two years post-AHSCT compared to baseline across all patients. Dispersion measures are represented by the median, along with the minimum and maximum log_2_ fold change values The summary graph **(D)** shows the proportions of non-switched IgD^+^IgM^+^ memory B cells (cluster g) in each patient at baseline and two years post-AHSCT. Patients with a relapse post-AHSCT are highlighted in red, while one patient with a new T2 lesion post-AHSCT is marked in turquoise. Statistical analyses were performed with the Wilcoxon matched-pair test (***p<0.001).

Given the effectiveness of B-cell-depleting therapies in MS treatment, we sought to analyze the B-cell population in greater detail. Manually gated B cells (CD19^+^ CD20^+/-^ CD3^-^CD14^-^) from the B-cell/myeloid CyTOF panel 1 were subjected to dimensional reduction analysis using t-SNE, followed by cell clustering with the PhenoGraph algorithm and phenotypic characterization of clusters using Cluster Explorer. Two years post-AHSCT, the B-cell compartment exhibited a reduction in memory B cells and plasma cells, accompanied by a shift toward naïve B cells. In contrast, the proportions of transitional B cells (both TrB1 and TrB2) remained stable, except for one patient with prior rituximab therapy, who displayed elevated TrB2 levels at baseline. Interestingly, patients with EIDA had higher baseline levels of non-switched IgD^+^IgM^+^ memory B cells, suggesting a potential predictive biomarker (**Figures 4A–D**, **Supplementary Figures 2**; **S2.3A–Q**).

### 3.6 Differential decline in central memory CD4 T cells subsets and expansion of PD-1^+^ effector memory CD4 T cells were associated with remission

Given the well-established role of T cells in the pathogenesis of multiple sclerosis (MS), we sought to characterize the phenotype of CD4^+^ and CD8^+^ T-cell populations in greater detail. CyTOF data from the T-cell panel (CyTOF, panel 2) were analyzed using a sequential workflow: first, CD4 and CD8 T cells were manually gated, followed by dimensionality reduction using t-SNE, cell subset clustering with PhenoGraph, and phenotypic characterization of clusters using Cluster Explorer. Naïve T cells were defined as CD45RA^+^CD28^+^CD27^+^, memory T cells (primarily central memory with some effector memory) as CD45RA^-^CD28^+^CD27^+^, effector memory T cells as CD45RA^-^CD28^+^CD27^-^, and T_EMRA_ cells as CD45RA^++^CD28^-^CD27^-^. Due to poor data quality, two CyTOF runs were excluded from the analysis, affecting samples from five AHSCT patients (pre- and post-treatment), five ND samples, and three HC.

**Figure 5:**
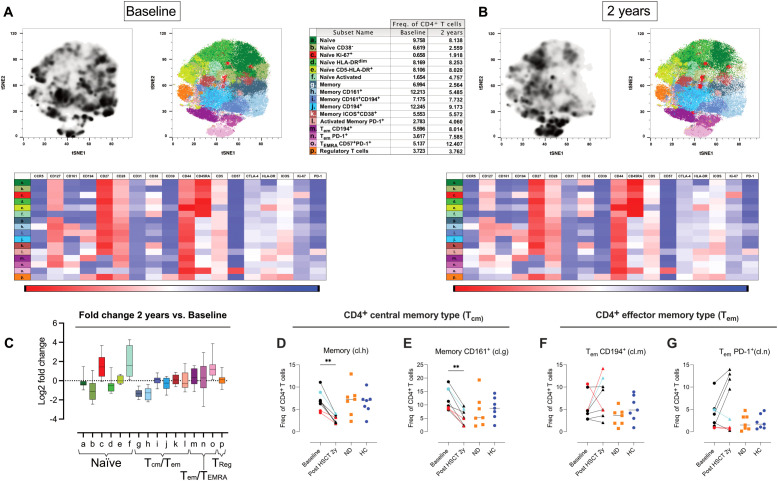
Shifts in naïve, central memory, and effector CD4 T cell phenotypes after AHSCT. Analysis of mass cytometric data (CyTOF, panel 2) of the CD4 T cell population (CD45^+^CD3^+^CD4^+^CD8^-^ CD19^-^CD20^-^CD14^-^) in PBMCs from MS patients at baseline (n=8) and two years post-AHSCT (n=8). The CyTOF data were concatenated, CD4 T cells were manually gated and subjected to high-dimensional reduction analysis of the CD4 T cell population using t-SNE, followed by Phenograph clustering and heatmap analysis of marker expression within distinct cell clusters, visualized using Cluster Explorer. The distribution of CD4 T cell clusters is shown at baseline **(A)** and two years post-AHSCT **(B)**, along with a table summarizing the mean relative frequencies of each cluster at both time points. The heatmaps show mean expression levels of cellular markers in each cell cluster. The summary graph **(C)** depicting log_2_ fold changes in the frequencies of phenotypically distinct CD4 T cell clusters at two years post-AHSCT compared to baseline across all patients. Dispersion measures are represented by the median, along with the minimum and maximum log_2_ fold change values. Relative frequencies of central memory (Tcm) CD4 T cells expressing no additional markers included in the panel (cluster h) **(D)** and CD4 Tcm T cells expressing CD161 (cluster g) **(E)**. Effector memory T cells (T_em_), which have lost CD27 expression, CD194^+^/CCR4^+^ (cluster m) **(F)**, and T_em_ enriched for PD-1^+^ cells (cluster n) **(G)**. Patients with a relapse post-AHSCT are highlighted in red, while one patient with a new T2 lesion post-AHSCT is marked in turquoise. Statistical analyses were performed with the Wilcoxon matched-pair test (**p<0.01).

Two years post-AHSCT, naïve CD4 T cells (cluster a, b. d, e), phenotypical separated by HLA-DR, CD38 and CD5 expression, exhibited a modest decline in all patients except for one patient who experienced clinical relapse during the observation period (**Figures 5A–C**, **Supplementary Figures 2**; **S2.4A, B, D, E**). In contrast, two clusters of naïve CD4 T cells significantly expanded: one enriched for cells expressing CD194/CCR4 (cluster c) and another composed of naïve-like CD4 T cells lacking CD127 (cluster f) (p<0.05 and p<0.01 respectively) (**Figures 5A–C**, **Supplementary Figures 2**; **S2.4C, F**).

Within the memory CD4 T-cell compartment, a shift from central memory (T_cm_) to effector memory (T_em_) phenotypes was observed two years post-AHSCT. At this timepoint, the frequency of T_cm_ CD4 T cells lacking additional defining markers (cluster g) and those expressing CD161, associated with IL-17 production, (cluster h) were consistently reduced across all patients (p<0.01) (**Figures 5D, E**). In contrast, memory CD4 T cells expressing CD194/CCR4 with or without CD161, as well as memory cells with an activated phenotype, remained at baseline levels (clusters i-l) (**Figures 5A–E**, **Supplementary Figures 2**; **S2.4I–L**). Two distinct clusters of T_em_ lacking CD27 were identified, indicative of a more antigen-experienced phenotype (cluster m and n). (**Figures 5F, G**). These clusters differed in their expression of CD194/CCR4 and the immune checkpoint receptor PD-1. The relative proportions of these subsets varied more between patients at two years post-AHSCT compared to baseline. Notably, two of the three patients with EIDA exhibited the highest frequencies of CD194/CCR4^+^ T_em_ (cluster m) but among the lowest frequencies of PD-1^+^ T_em_ (cluster n) (**Figures 5F, G**). In contrast, all but one patient in remission showed an increased proportion of PD-1^+^ T_em_ (cluster n) (**Figure 5G**). Additionally, the proportion of effector/T_EMRA_ CD4 T cells expressing high levels of CD57 (cluster o) was significantly increased two years post-AHSCT, though some inter-patient variability was observed at this timepoint (**Figures 5A–C**, **Supplementary Figures 2**; **S2.4O**). Regulatory T cells (T_regs_; cluster p), characterized by central memory-like properties and high expression of CD39, CD194, and ICOS, maintained frequencies similar to baseline (**Figures 5A–C**, **Supplementary Figures 2**; **S2.4P**).

### 3.7 Increased effector memory and T_EMRA_ phenotype in CD8 T cells two years post-AHSCT

CD8 T cells exhibited greater phenotypic diversity than CD4 T cells. Two years post-AHSCT, naïve CD8^+^ T-cell subsets (clusters a and b), (CyTOF panel 2) distinguished by CD31 expression, showed a slight decline in most patients, except for one individual who had a clinical relapse (**Supplementary 2**, **Supplementary Figures 2**; **S2.5A**). In contrast, significant reductions were observed in CD38^-^ naïve CD8 T cells, (cluster c, p<0.05), HLA-DR^+^, (cluster d, p<0.01) and a small population of proliferating naïve-like cells lacking CD127 (cluster f, p<0.05), with this trend consistent across all patients. (**Supplementary 2**, **Supplementary Figures 2**; **S2.5C, D, F**)

**Figure 6:**
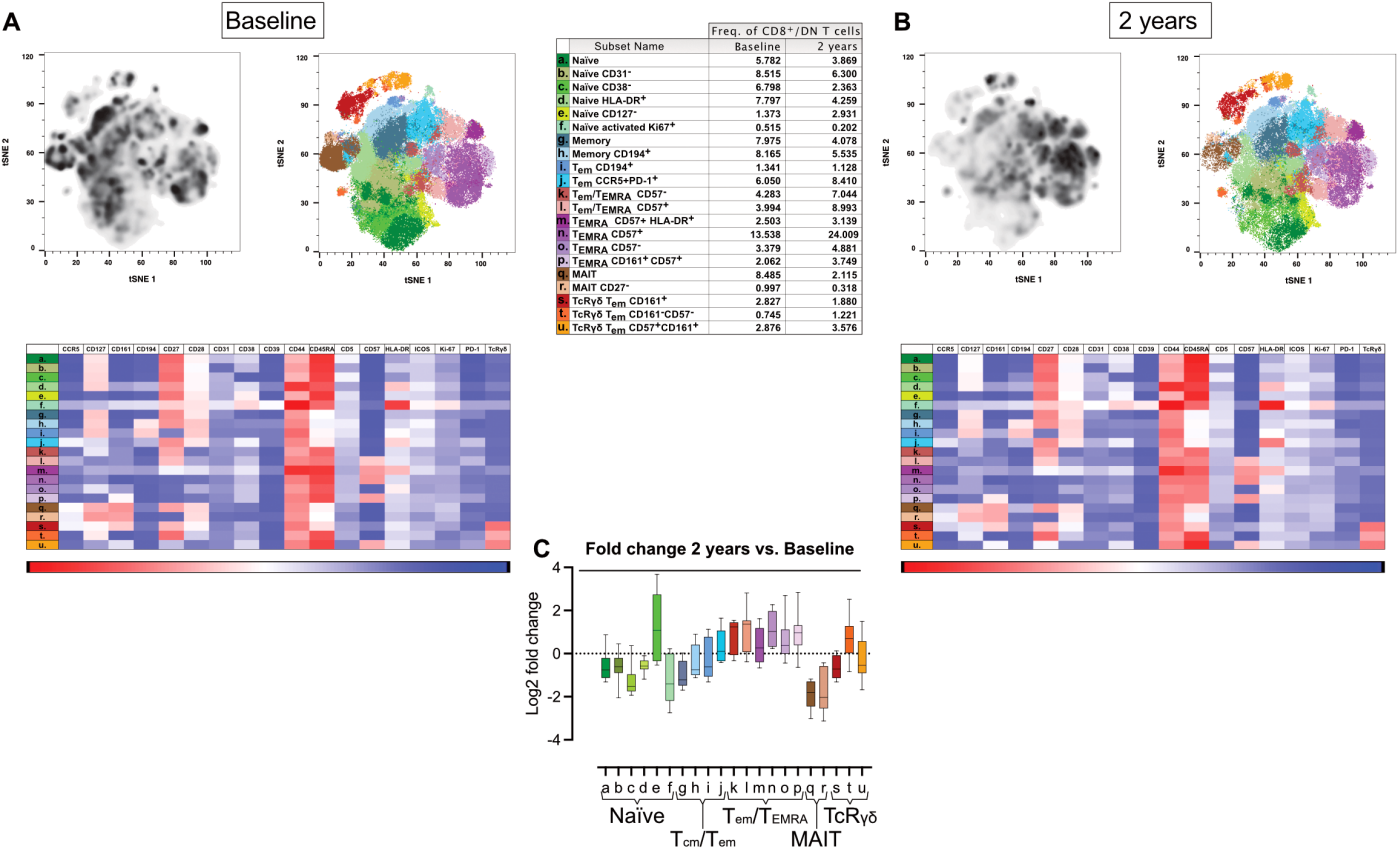
Phenotypic diversity and T_EMRA_ expansion in CD8 T cells two years post-AHSCT. Analysis of mass cytometric data (CyTOF, panel 2) of the CD8/DN T cell population (CD45^+^CD3^+^CD8^+/-^CD4^-^ CD19^-^CD20^-^CD14^-^) in PBMCs from MS patients at baseline (n=8) and two years post-AHSCT (n=8). The CyTOF data were concatenated, CD8/DN T cells were manually gated and subjected to high-dimensional reduction analysis of the CD8 T cell population using t-SNE, followed by Phenograph clustering and heatmap analysis of marker expression within distinct cell clusters, visualized using Cluster Explorer. The distribution of CD8 T cell clusters is shown at baseline **(A)** and two years post-AHSCT **(B)**, along with a table summarizing the mean relative frequencies of each cluster at both time points. The heatmaps show mean expression levels of cellular markers in each cell cluster. The summary graph **(C)** depicting the log_2_ fold changes in the frequencies of phenotypically distinct CD8 T cell clusters at two years post-AHSCT compared to baseline across all patients. Dispersion measures are represented by the median, along with the minimum and maximum log_2_ fold change values.

Meanwhile, memory CD8 T cells shifted toward a more antigen-experienced effector memory/exhausted phenotype, characterized by increased frequencies of both CD57^+^ and CD57^-^ T_EMRA_ subsets (p<0.05 and p<0.01 respectively). Notably, patients in remission displayed higher T_EMRA_ cell frequencies compared to those with EIDA (**Figures 6A–C**, **Supplementary Figures 2**; **S2.5G–P**).

Mucosal-associated invariant T (MAIT) cells, identified by CD161 and CCR5 expression, declined consistently across all patients, whereas TcRγδ T cells (clusters s-u) remained at baseline frequencies (**Figures 6A–C**, **Supplementary Figures 2**; **S2.5Q–U**).

### 3.8 Extended T cell phenotyping with flow cytometry

Building on the CyTOF data, we sought to further characterize circulating T-cell phenotypes before and after AHSCT in a larger group of AHSCT-treated patients, incorporating both earlier and later time points for reference. To this end, we designed three traditional 7-color flow cytometry panels to assess T-cell maturation, functionality (Th-phenotype), and homing properties.

These panels included markers for CD62L, CCR7 (lymph node homing) and CD45RO, absent in the mass cytometric panel, as well as CXCR3, CCR5, CCR6, and CXCR6 (tissue homing and Th-phenotype). For definition of CD4 Th-phenotype see **Supplementary Figures 1**; **S1.16**. Additionally, one panel specifically analyzed PD-1 expression in relation to CXCR3 (Th1-associated) and CCR6 (Th17-associated) subsets.

#### 3.8.1 Persistent expansion of PD-1+Th1 effector memory, decreased Th17 and expansion of CD62L- naïve CD4 T cells two years post-AHSCT

##### 3–6 Months post-AHSCT

**Figure 7:**
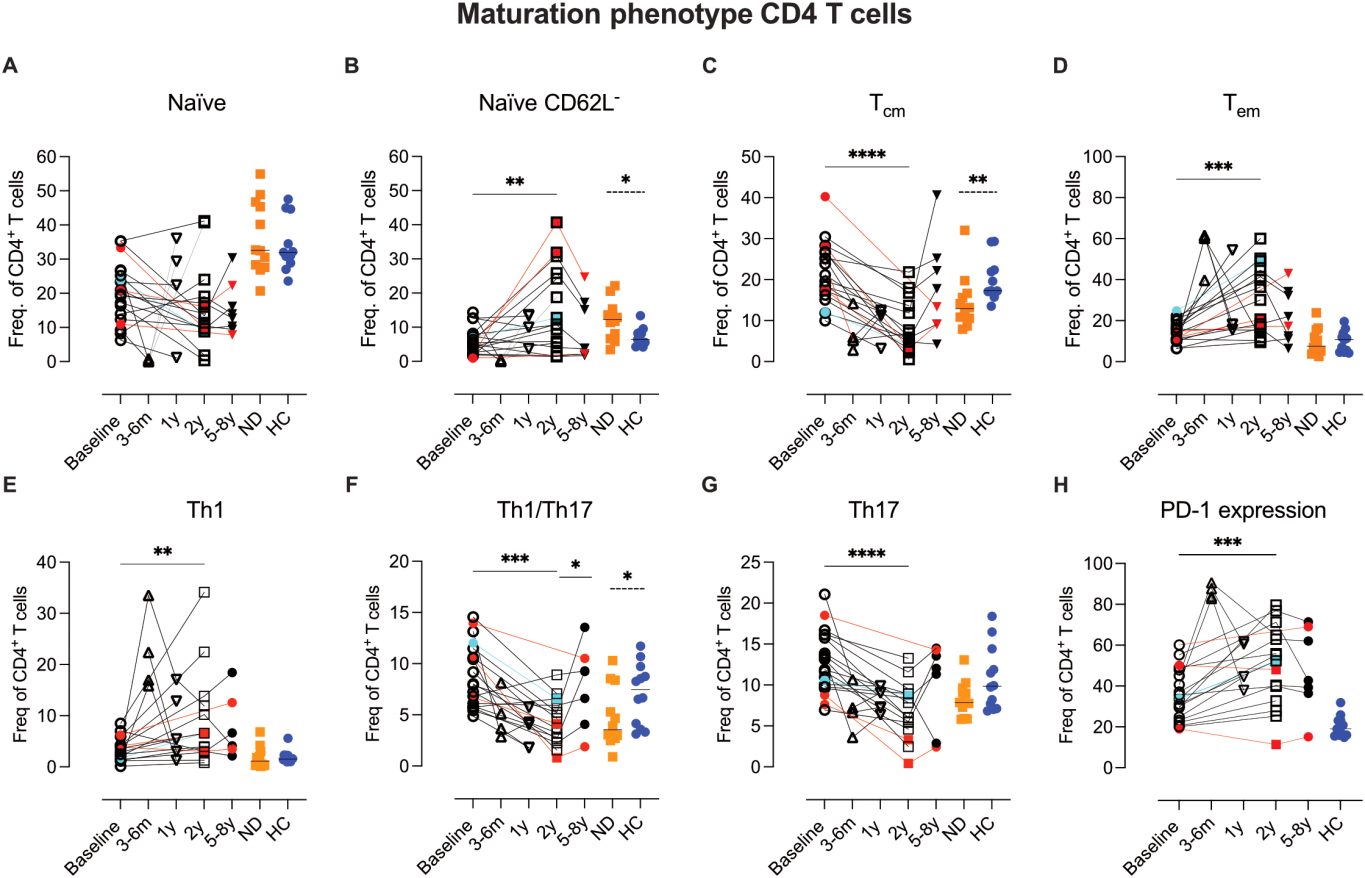
Variations in Th1/Th17 balance, PD-1 expression and naïve phenotype in CD4 T cell subsets two years post-AHSCT. Summary graphs of flow cytometric analysis depicting the relative frequencies of maturation, homing, and functional phenotypes of CD4 T cells in PBMCs from MS patients at baseline (n=20), 4–6 months (n=4), 1 year (n=4), 2 years (n=15-16), and 5–8 years (n=8) post-AHSCT, as well as in newly diagnosed MS patients (ND) (n=12) (not analyzed for PD-1) and healthy controls (HC) (n=11). The maturation profiles include naïve CD4 T cells **(A)**, naïve CD62L^-^ cells **(B)**, central memory T cells (T_cm_) **(C)**, and effector memory T cells (T_em_) **(D)**. Functional T helper phenotypes were assessed using chemokine receptor expression patterns, defining Th1 cells (CXCR3^+^CCR5^+^CCR6^-^CXCR6^-^) **(E)**, Th1/17 cells (CXCR3^+^CCR5^+^/^-^CCR6^+^CXCR6^-^) **(F)**, and Th17 cells (CXCR3^-^CCR5^+^/^-^CCR6^+^CXCR6^-^) **(G)**. Proportions of CD4 T cells expressing the immune inhibitory receptor PD-1 **(H)**. Frequencies shown in the graphs were calculated as a proportion of total CD4 T cells. Patients with a relapse post-AHSCT are highlighted in red, while one patient with a new T2 lesion post-AHSCT is marked in turquoise. Statistical analyses were performed using the Wilcoxon matched-paired test used (solid line, *p<0.05, **p<0.01, ***p<0.001, **** p<0.0001) to compare paired samples and the Mann-Whitney test (hatched line, *p<0.05 and **p<0.01) to compare unpaired groups.

**Figure 8:**
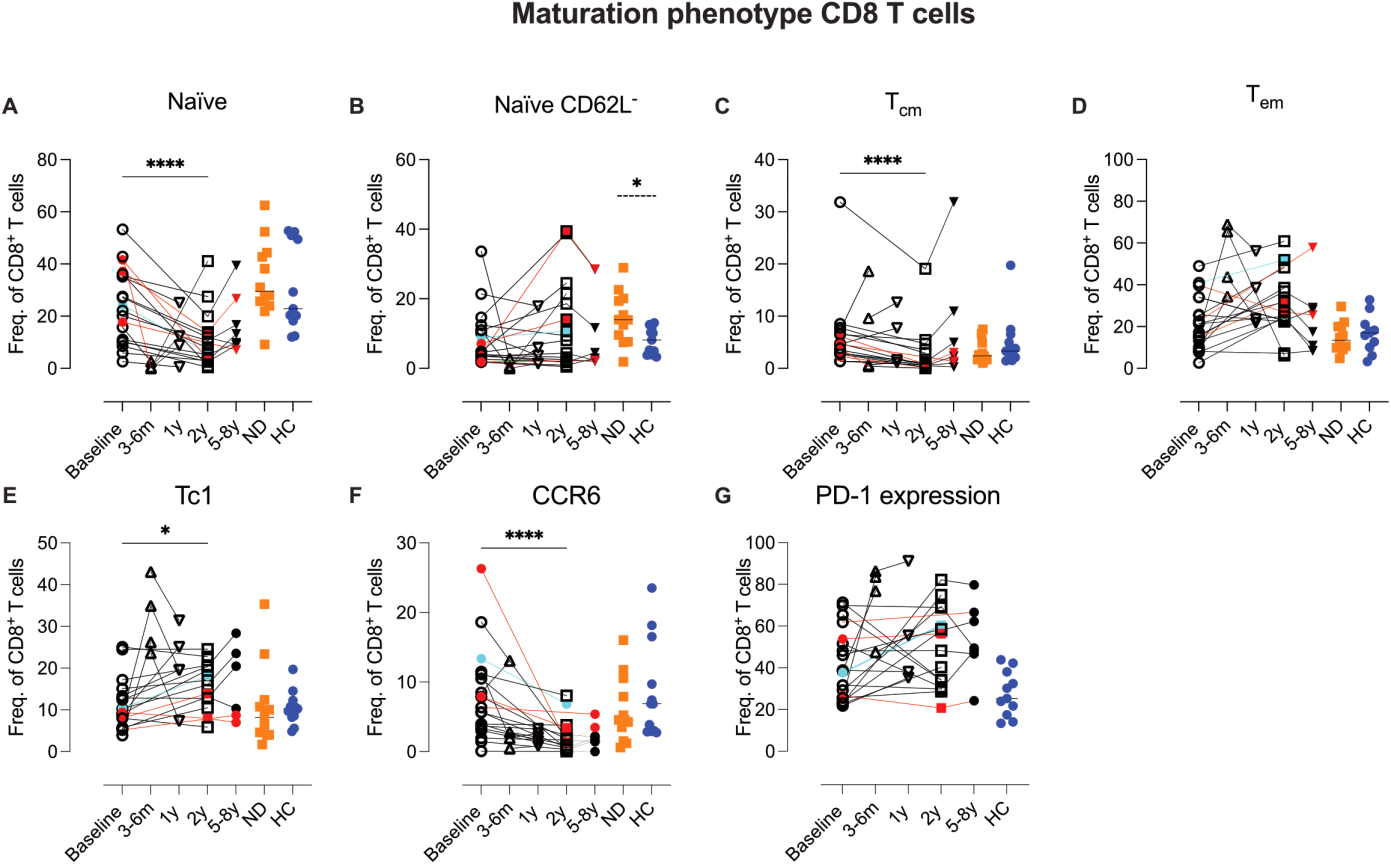
Shift toward increased Tc1 and decreased frequencies of naïve and central memory CD8 T cells post-AHSCT. Summary graphs of flow cytometric analysis depicting the relative frequencies of maturation, homing, and functional phenotypes of CD8 T cells in PBMCs from MS patients at baseline (n=20), 4–6 months (n=4), 1 year (n=4), 2 years (n=15-16), and 5–8 years (n=8) post-AHSCT, as well as in newly diagnosed MS patients (ND) (n=12) (not analyzed for PD-1) and healthy controls (HC) (n=11). The maturation profiles include naïve CD8 T cells **(A)**, naïve CD62L^-^ cells **(B)**, central memory T cells (T_cm_) **(C)**, and effector memory T cells (T_em_) **(D)**. The Tc1 phenotype of CD8 T cells was assessed based on chemokine receptor expression patterns, including CXCR3^+^CCR5^+^CCR6^-^CXCR6^-^ cells **(E)** and CCR6 expression **(F)**. Proportions of CD8 T cells expressing PD-1 **(G)**. Frequencies in the graphs were calculated as a proportion of total CD8 T cells. Patients with a relapse post-AHSCT are highlighted in red, while one patient with a new T2 lesion post-AHSCT is marked in turquoise. Statistical analyses were performed using the Wilcoxon matched paired test (solid line, *p<0.05, ****p<0.0001) to compare paired samples and the Mann-Whitney test (hatched line, *p<0.05) to compare unpaired groups.

Most CD4 and CD8 T cells exhibited an overall effector memory phenotype with high PD-1, CCR5, and CXCR3 expression, indicating a Th1/Tc1 activated/exhausted profile (**Figures 7A–H**, **8A–G**, **Supplementary Figures 2**; **S2.6-S2.9**). However, due to the small sample size no statistical analysis was performed.

##### 1 Year post-AHSCT

A trend toward partial recovery of naïve T cells was observed, with reduced PD-1 and chemokine receptor expression. However, variability persisted among CD8 T cell phenotypes (**Supplementary Figures 2**; **S2.8, S2.9**). Again, due to the small sample size no statistical analysis was performed.

##### 2 Years post-AHSCT

Two years post-AHSCT, the proportions of CD4 naïve T cells remained comparable to baseline levels (**Figure 7A**). However, a significant increase in a CD62L^-^ naïve subset was observed, particularly in patients who relapsed, suggesting activation and potential TCR engagement in these cells (p<0.01) (**Figure 7B**). In the memory compartment, T_cm_ cells were significantly reduced (p<0.001), while effector memory T_em_ cells were increased (p<0.0001) (**Figures 7C–D**). Additionally, Th1 cell frequencies were elevated (p<0.01), whereas Th1/Th17 and Th17 subsets declined (p<0.001 and p<0.0001 respectively) (**Figures 7E–G**). PD-1 expression remained significantly upregulated (p<0.001) however variability across patients was seen at this time point (**Figure 7H**).

**Figure 9:**
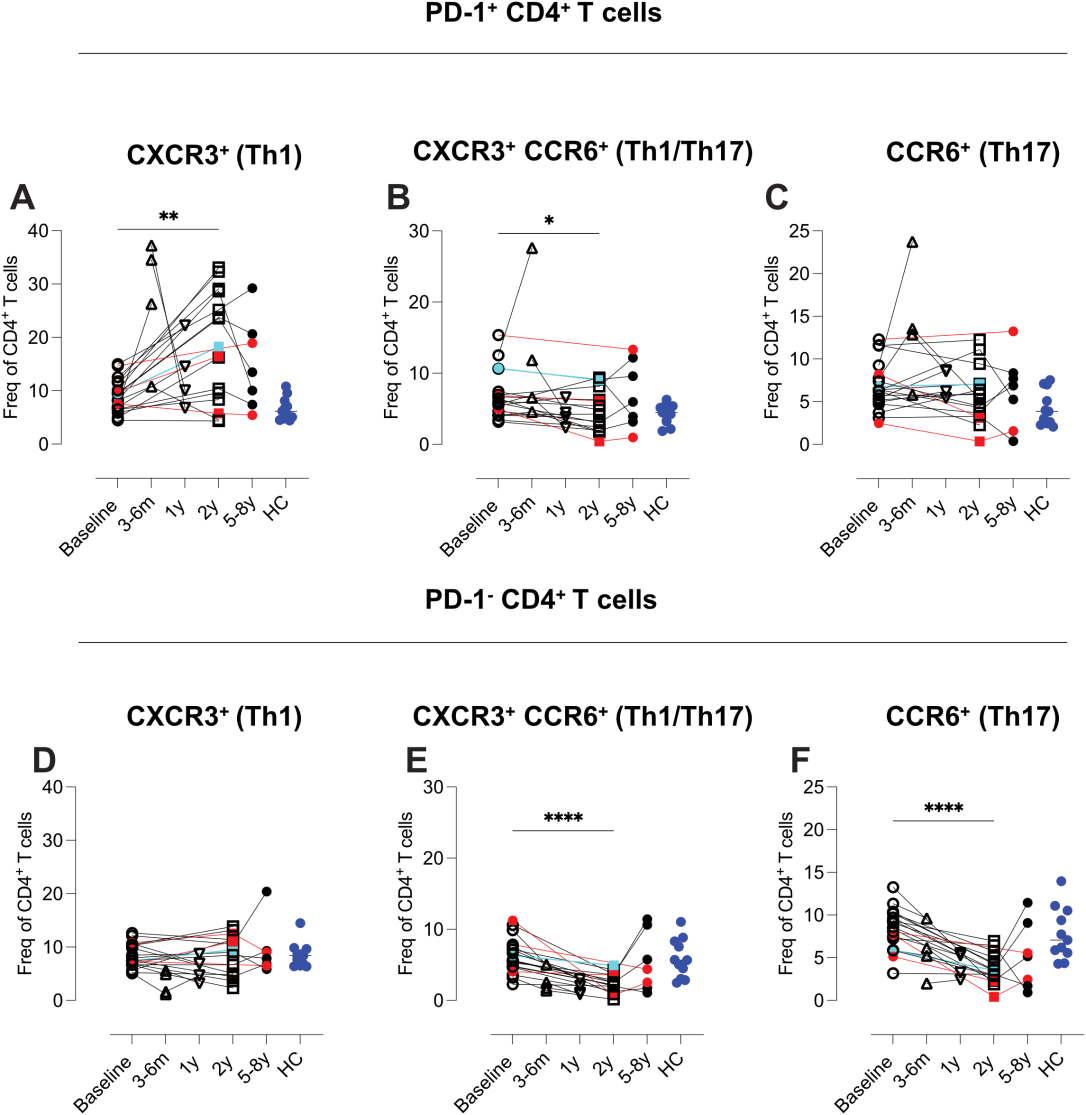
Selective expansion of PD-1^+^ Th1 CD4 T cells and reduction of PD-1^-^ Th17 subsets. Flow cytometric analysis of co- expression pattern PD-1, CXCR3 and CCR6, Summary graphs depicting CXCR3 and CCR6 expression on CD4 T cells, stratified by PD-1 expression, in PBMCs from MS patients at baseline (n=20), 4–6 months (n=4), 1 year (n=4), 2 years (n=16), and 5–8 years (n=7) post-AHSCT and healthy controls (HC) (n=11). The analyzed subsets include PD-1^+^CXCR3^+^CCR6^-^ **(A)**, PD-1^+^CXCR3^+^CCR6^+^ **(B)**, PD-1^+^CXCR3^-^CCR6^+^ **(C)**, PD-1^-^CXCR3^+^CCR6^-^ **(D)**, PD-1^-^CXCR3^+^CCR6^+^ **(E)**, and PD-1^-^CXCR3^-^CCR6^+^ **(F)**. Frequencies in the graphs were calculated as a proportion of total CD4 T cells. Patients with a relapse post-AHSCT are highlighted in red, while one patient with a new T2 lesion post-AHSCT is marked in turquoise. Statistical analyses were performed with the Wilcoxon matched-paired test (*p<0.05, **p<0.01, ****p<0.0001).

Further analysis revealed a preferential enrichment of Th1 cells within the PD-1^+^ CD4 T-cell subset (p<0.01), while the decline in CD4 T cells enriched for Th1/Th17 (p<0.001) and Th17 (p<0.0001) was primarily observed among PD-1^-^ cells (**Figures 9A–F**, **Supplementary Figures 2**; **S2.10**).

Among CD45RA^+^RO^-^ CD8 T cells, encompassing both naïve and antigen-experienced T_EMRA_ cells, a significant reduction in naïve cells was observed (p<0.0001), while CD62L^-^ naïve CD8 T cells and T_EMRA_ cells returned to baseline levels (**Figures 8A, B**, **Supplementary Figures 2**; **;S2.7E, D**). The maturation phenotype of memory CD8 T cells paralleled that of CD4 T cells, with a significant reduction in T_cm_ cells (p<0.0001) and an increase in T_em_ cells in most patients (**Figures 8C, D**). Tc1 CD8 cells showed a slight increase (p<0.05), while CCR6^+^ CD8 T cells decreased significantly (p<0.0001), likely due to a reduction in CCR6^+^ MAIT cells (**Figures 8E, F**). In contrast, PD-1 expression in CD8 T cells exhibited variability across patients and did not reach statistical significance (**Figure 8G**).

##### 5–8 Years post-AHSCT

At later timepoints the overall phenotype of CD4 and CD8 T cells appeared to normalize, with slight increases in Th1/Th17 subsets and a trend toward reduced CD62L^-^ naïve T cells (**Figures 7**, **8**). Due to the small sample size, no statistical analysis was performed.

### 3.9 Reduction in classical MAIT cells and sustained frequencies of TcRVα7.2^+^ immature/non MAIT post-AHSCT

In the CyTOF analysis, we observed a reduction in MAIT cells two years post-AHSCT (identified using CD161 and CCR5 as lineage markers). To further characterize these populations, we employed a seven-color flow cytometry panel incorporating the invariant T-cell receptor TcR Vα7.2, along with CD161, IL-18R, and CXCR6.

**Figure 10:**
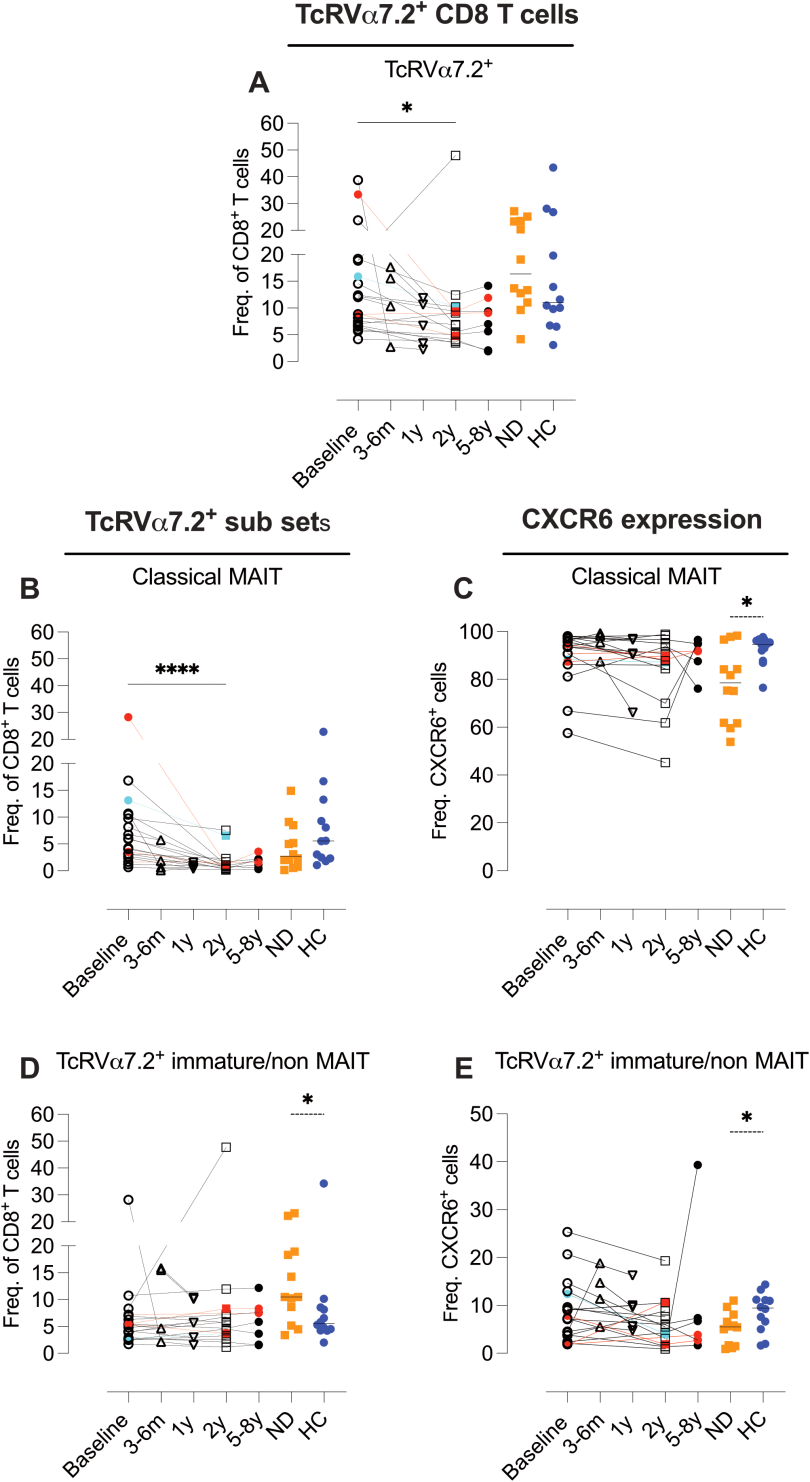
Post-AHSCT alterations in TcRVα7.2^+^ T Cells, decreased classical MAIT cells with preserved immature subsets. Flow cytometric analysis of the proportions of CD8 T cells expressing the invariant T cell receptor TcR Vα7.2 **(A)**, classical MAIT CD8 T cells defined as TcR Vα7.2^+^IL-18R^+^CD161^++^ **(B)**, and CXCR6 expression within the classical MAIT cell subset **(C)**. Immature/non-MAIT CD8 T cells, defined as TcR Vα7.2^+^IL-18R^+^/^-^CD161^+^/^-^, **(D)**, frequencies of CXCR6 expressing Immature/non-MAIT CD8 T cells **(E)**. Flow cytometric analysis was performed on PBMCs from MS patients at baseline (n=20), 4–6 months (n=5), 1 year (n=5), 2 years (n=16), and 5–8 years (n=7) post-AHSCT, as well as from newly diagnosed MS patients (ND) (n=12) and healthy controls (HC) (n=11). Frequencies presented in **(A, B, D)** were calculated as a proportion of all CD8 T cells, while frequencies in **(C, E)** were calculated relative to the respective TcR Vα7.2^+^ cell subset. Patients with a relapse post-AHSCT are highlighted in red, while one patient with a new T2 lesion post-AHSCT is marked in turquoise. Statistical analyses were performed using the Wilcoxon matched-pair test (solid line, *p<0.05, ****p<0.0001) to compare paired samples and the Mann-Whitney test (hatched line, *p<0.05) to compare unpaired groups.

Two years post-AHSCT, the overall proportion of TcR Vα7.2^+^ CD8 T cells exhibited a modest but significant decline (p<0.05) (**Figure 10A**). Further stratification into classical MAIT cells (TcR Vα7.2^+^CD161^+^IL-18R^+^) and immature/non-MAIT cells (TcR Vα7.2^+^CD161^-^/^+^IL-18R^-^/^+^) revealed a pronounced reduction in classical MAIT cells (p<0.0001), whereas the frequencies of immature/non-MAIT cells remained stable across all time points (**Figures 10B, D**, **Supplementary Figures 2**; **S2.12**).

Classical MAIT cells exhibited high CXCR6 expression, while immature/non-MAIT cells displayed minimal CXCR6 expression (**Figures 10C, E**, **Supplementary Figures 2**; **S2.12E**). Within the double-negative (DN) T-cell subset, the proportions of classical and immature MAIT cells closely mirrored those observed in the CD8 T-cell compartment. In contrast, these subsets remained consistently low and unchanged in the CD4 T-cell population at all time points (**Supplementary Figures 2**; **S2.11, S2.12**).

### 3.10 Enhanced HLA-DR and CCR5 expression in regulatory T cells post-AHSCT

The CyTOF analysis of CD4 T cells revealed stable frequencies of T_regs_ at baseline and two years post-AHSCT. To further characterize their phenotype, we performed flow cytometric analyses of FoxP3, Helios, CD62L, CCR5, and HLA-DR.

Consistent with CyTOF data, the overall proportion of FoxP3^+^ CD4 T cells remained unchanged two years post-AHSCT (**Supplementary Figures 2**; **S2.13A–C**). However, within the FoxP3^+^Helios^+^ subset, commonly referred to as natural Tregs (nTregs) or thymic-derived Tregs (tTregs), a slight but significant reduction in relative frequency was observed (p<0.05). In contrast, FoxP3^+^Helios^-^ Tregs (inducible Tregs, iTregs) maintained frequencies comparable to baseline (**Supplementary Figures 2**; **S2.13D–G**).

Both Treg subsets exhibited an enhanced activation phenotype, as indicated by reduced CD62L expression (p<0.01) and an increase in CCR5^+^ and HLA-DR^+^ cells, particularly within the FoxP3^+^Helios^+^ subset (p<0.01 and p<0.001, respectively) (**Supplementary Figures 2**; **S2.14A–H**).

## 4 Discussion

This study provides a comprehensive immunological characterization of patients with RRMS undergoing AHSCT. By leveraging mass cytometry and flow cytometry, we identified profound and durable shifts in immune cell subsets and functional phenotypic profiles, that likely contribute to the long-term efficacy of AHSCT in MS. Our findings offer new insights into how immune reconstitution may support disease remission, highlighting key alterations in B cells, myeloid cells, and T cells.

### 4.1 B cell alterations following AHSCT and implications for MS

While AHSCT is known to reset the immune system and reduce disease activity, most previous studies have focused on T-cell reconstitution, leaving the B-cell compartment relatively underexplored. Our findings align with prior studies demonstrating that B cells are efficiently depleted following AHSCT, yet recover rapidly and exceed normal levels within one year post-transplantation (9, 13). The predominance of naïve B cells post-AHSCT, which remains stable for at least two years, mirrors the natural development of memory B cells in childhood, where memory B-cell frequencies gradually stabilize by the age of five (14).

The profound depletion of memory B cells and plasmablasts, coupled with the expansion of naïve B cells, supports the notion that AHSCT effectively resets the B-cell compartment, reducing their capacity for antigen presentation and autoantibody production. Notably, patients with EIDA displayed a higher proportion of non-switched IgD^+^IgM^+^ memory B cells pre-AHSCT, suggesting a role for this subset in MS pathophysiology and highlighting its potential as a predictive biomarker for treatment response, though this needs confirmation in larger studies.

Unswitched IgD^+^IgM^+^ memory B cells, characterized by the expression of CD27 along with IgM and IgD, represent a key subset of the memory B-cell lineage. These cells play a fundamental role in immune surveillance and response, particularly by serving as a first line of defense against pathogens (15). Strong evidence supports the existence of two distinct subsets of IgD^+^IgM^+^CD27^+^ B cells: one generated independently of germinal centers and predominating in early life, and another with key post-germinal center memory B-cell characteristics that dominate in adulthood. These IgD^+^IgM^+^CD27^+^ B cells can differentiate into IgM-secreting plasma cells, providing highly efficient complement fixation, but they also have the unique ability to re-enter germinal center reactions upon re-exposure to the same or a related antigen. This allows for adaptation of their B-cell receptor specificity to altered antigens through new rounds of somatic hypermutation and selection (15).

In general, increased frequencies of memory B cells have been associated with an MRI phenotype with high neurodegeneration, defined by increased numbers of contrast-enhancing lesions and non-enhancing black holes on T1-weighted images, and reduced brain parenchymal fraction (16). Anti-CD20 monoclonal antibodies are very potent B-cell depleting agents and effectively deplete memory B cells (17) and have been shown to prevent new T2 lesions and clinical relapses (18, 19). In contrast, tabalumab, an anti-BAFF monoclonal targeting BAFF, mainly affecting transitional and mature B cells while sparing memory B cells, had no clinical effect in RRMS patients (20) Instead, treatment with tabalumab led to an increase in circulating memory B cells, further underscoring the importance of memory B-cell depletion for therapeutic efficacy (21).

The role of IgD^+^IgM^+^CD27^+^ B cells in the pathophysiology of MS is less clear, but they have increasingly been implicated in MS pathogenesis. Studies have demonstrated that these cells are capable of supporting ectopic lymphoid structures within the CNS. These structures serve as niches for ongoing inflammation and autoantibody production, contributing to chronic disease activity (22, 23). Moreover, unswitched memory B cells play a role in antigen presentation, engaging with autoreactive T cells and amplifying the autoimmune response (23). Their ability to produce pro-inflammatory cytokines, such as IL-6 and GM-CSF, further underscores their contribution to pathogenic inflammation in MS. Conversely, their production of the regulatory cytokine IL-10 appears limited in disease contexts, reflecting a dysregulation of their normal immune-modulating functions (22, 23). Such observations suggest that unswitched memory B cells may preserve autoreactive T cell clones that are resistant to AHSCT. This hypothesis is further supported by the finding that rituximab administration in close proximity to AHSCT is highly protective for relapses post-AHSCT (24).

### 4.2 Myeloid cell shifts and potential effects on neuroinflammation

Myeloid cells, particularly monocytes and macrophages, are known to infiltrate the CNS in MS and contribute to neuroinflammation, demyelination, and lesion formation (25, 26). In our study we observed a significant reduction in classical monocytes post-transplant, while non-classical monocytes increased. This shift could be important, as classical monocytes are major producers of pro-inflammatory cytokines such as TNF-α, IL-6, and IL-1β, which drive MS progression (27). Conversely, non-classical monocytes exhibit a patrolling function and possess regulatory properties, limiting excessive inflammation. This may represent a shift toward immune regulation rather than inflammation, potentially contributing to long-term remission in MS.

We also observed an increase in pDC post-AHSCT. Classically, these have been associated with antiviral responses and type I interferon production (28). This may be related to the shift from a Th17 to a Th1 type response, but could also reflect a response to viral replication post-AHSCT in the immunocompromised host. Future research focusing on functional assessments of these altered myeloid populations will be critical to understanding their roles in maintaining immune tolerance and preventing MS relapse.

### 4.3 T-cell reconstitution and immune balance post-AHSCT

T cells play a central role in multiple sclerosis (MS) pathogenesis, with both CD4^+^ and CD8^+^ subsets contributing to neuroinflammation and disease progression. Historically, MS has been considered a predominantly T-cell-driven autoimmune disorder, supported by findings of T-cell infiltration into CNS lesions and the strong genetic association with HLA-DRB1*15:01 (29). While early studies emphasized a Th1-driven immune pathology, more recent evidence suggests that disease progression is also influenced by Th1/Th17-mediated responses (30, 31). Given the critical role of T cells in MS, understanding their reconstitution post-AHSCT is essential for elucidating mechanisms of immune reset and sustained remission.

Our results reaffirm the critical role of immune ablation and subsequent reconstitution in achieving durable remission. The observed reduction in CD4 T-cell counts at six months, one year, and two years post-AHSCT aligns with previous findings (5), underscoring the transient depletion of lymphocyte subsets as an expected outcome of high-dose immunosuppressive conditioning. In contrast, CD8 T-cell counts remained relatively stable. The shifts in the CD4/CD8 ratio, persisting in many patients at least for two years, mirror the long-lasting effect of AHSCT. Importantly, patients exhibiting EIDA post-AHSCT retained normal CD4 counts and ratios, suggesting that the CD4/CD8 ratio is a potential biomarker for incomplete immune reset.

### 4.4 Atypical naïve CD4 T cells and their role in MS

Recovery of naïve CD4 T cells following AHSCT is known to depend on thymic function, with reconstitution typically beginning around one year post-transplant. At the two-year mark, we observed inter-patient variability in naïve CD4 T-cell frequencies, however those with EIDA were among the patients with the highest proportions, suggesting that this is associated with enhanced thymic output. Further analysis of the CD45RA^+^CD45RO^-^ subset revealed an increase in naïve CD4^+^ T cells lacking CD62L post AHSCT. Although most patients exhibited a slight increase in these atypical naïve cells, the highest frequencies were observed in those who relapsed. Interestingly, newly diagnosed MS patients also displayed a significant elevation of naïve CD4^+^CD62L^-^ T cells compared to healthy controls, suggesting that this subset may be relevant to early disease processes.

Naïve and T_cm_ CD4 T cells typically express CD62L and CCR7, which facilitate their recirculation through lymphoid tissues. Upon antigen engagement, CD62L downregulation enables effector differentiation and tissue migration. However, CD62L downregulation can also occur independently of TCR signaling, driven by cytokines, ATP, glucocorticoids, and metalloprotease activity (32). Atypical CD62L^-^ naïve CD4 T cells have been described in rheumatoid arthritis (RA), where they correlate with disease flares and exhibit IL-1/IL-6/TNF-driven inflammatory signatures (33). A similar subset has been linked to relapses in a rat model of MS (34), suggesting a role in autoimmune pathology. These cells may serve as a pool of pre-activated, bystander-responsive CD4 T cells, capable of amplifying CNS inflammation when recruited to affected tissues.

The increased proportions of CD62L^-^ naïve CD4 T cells post-AHSCT may reflect an inflammatory milieu favoring cytokine-driven activation. Alternatively, their presence in both relapsed and remission patients suggest a regulatory function in preventing excessive T-cell activation. Future studies should examine their TCR repertoire to determine whether they contribute to disease relapse or immune regulation post-AHSCT.

### 4.5 Th17 cells, CD4 Subsets, and immune modulation

Our flow cytometric analysis of CD4 T cells post-AHSCT revealed considerable inter-patient variability in immune reconstitution. However, certain shared features emerged two years post-transplant, including a consistent reduction in T_cm_ CD4 T cells, polyfunctional Th1/Th17 (CCR6^+^CXCR3^+^) cells, and Th17 (CCR6^+^CXCR3^-^) cells across all transplanted patients. These changes were accompanied by a variable increase in T_em_ CD4 T cells and a Th1-skewed phenotype (CXCR3^+^CCR5^+^CCR6^-^), findings that align with previous studies (5).

Using CyTOF analysis, where CD161 and CCR4 served as Th17 markers, we could further delineate these shifts. The observed reduction in T_cm_ CD4 T cells was predominantly restricted to two subsets: one lacking additional defining markers included in the CyTOF panel and another enriched for CD161^+^CCR4^-^ Th17 cells. In contrast, the frequencies of classical Th17 T_cm_ CD4 T cells co-expressing CD161 and CCR4 (CD194) or CCR4 alone (Th0/Th2) remained stable. Notably, the Th1/Th17 and Th17 subsets that decreased were predominantly PD-1^-^, whereas their PD-1^+^ counterparts were preserved. These findings suggest that the reduction in Th17 cells post-AHSCT is limited to a specific subset of Th17 T_cm_ cells. However, given that this decline occurred in all patients, regardless of clinical outcome, additional immunological mechanisms likely contribute to long-term disease remission.

In contrast to the more uniform contraction of T_cm_ subsets, the T_em_ CD4 T-cell compartment exhibited greater inter-patient variability, particularly in the Th1 subtype and regarding PD-1 expression. Notably, clusters enriched for PD-1^+^ T_em_ CD4 T cells differed between patients with and without clinical relapse. All but one patient in remission exhibited elevated frequencies of PD-1^+^ T_em_, whereas those who relapsed showed frequencies comparable to baseline. A similar, albeit less pronounced, trend was observed in CD4 T-cell clusters containing PD-1^+^CD57^+^ T_EMRA_ cells. Conversely, two patients with EIDA, including one who experienced a clinical relapse at the two-year time point, exhibited the highest frequencies of Th17-skewed T_em_ cells (CCR4^+^CD161^+^).

The role of T_regs_ in the pathology of MS is not completely understood and the interplay between immune regulatory and immune enhancing elements are complex. Genetic variants in CTLA-4 and CD25, along with altered functions and levels of T_regs_ in the circulation of MS patients have been reported (35, 36). Emerging evidence underscores the importance of brain-resident T_regs_ and their functional interactions with pathogenic effector T cells and other immune cells, suggesting a potential role in modulating neuroinflammation (37). Previous studies of AHSCT for MS have described an early transient rise in T_reg_ frequencies followed by return to baseline levels within 1 year post-AHSCT. The origin of these T_regs_ is presumed to be cells that escaped ablation or were re-infused with the graft (36, 38–40). At later time points, once thymic output is restored, the T_reg_ compartment would also be expected to reflect ongoing immune renewal (41).

In the present study, the overall proportions of CD4 with a T_reg_ associated phenotype were similar to baseline at two years post-AHSCT, but a slight decrease in the frequencies of a subset of natural T_regs_ (FoxP3^+^Helios^+^) was observed two years post-AHSCT. However, the phenotype of these T_regs_ cells showed an altered phenotype with decreased expression of CD62L, increased CCR5 and HLA-DR which resembles an activated effector memory type with possible CNS-migration properties.

### 4.6 CD8 T cell recovery and variability across patients

Although much of MS research has focused on CD4 T cells, emerging evidence suggests that CD8 T cells play a more significant role than previously appreciated. Histopathological analyses of post-mortem MS brain tissue have revealed a higher abundance of CD8 T cells compared to CD4 T cells. Additionally, studies in animal models have identified both myelin-reactive CD8 T cells and a potential regulatory function for this subset (42, 43). Given the distinct biological roles of CD4 and CD8 T cells, one plausible interpretation is that CD4 T cells initiate and drive the autoimmune process, whereas cytotoxic CD8 T cells act as effectors, executing tissue damage by targeting glial cells.

In the present study, we confirmed previous findings of a relatively rapid reconstitution of CD8 T cells in circulation post-AHSCT (5). The phenotypic profile of the CD8 compartment largely mirrored that of CD4 T cells, with a predominance of memory T cells at early time points post-transplant and a gradual recovery of naïve CD8 T cells over time.

At two years post-AHSCT, a shift from a naïve/T_cm_-dominant to a T_em_-skewed CD8 T-cell population remained evident, with significantly reduced proportions of naïve and T_cm_ CD8 T cells. However, substantial inter-patient variability was observed in chemokine receptor expression, PD-1 receptor expression, and distinct T_em_/T_EMRA_ CD8 T-cell CyTOF clusters.

Previous studies have reported an expansion of atypical terminally differentiated CD28^-^ CD57^+^ CD8 T cells post-AHSCT, which has been linked to immunosuppression (8, 44). Interestingly, in our cohort, patients with EIDA exhibited high proportions of both CD57^+^ and CD57^-^ CD8 cells lacking CD28 but co-expressing CD27. In contrast patients in remission showed increased proportions of highly differentiated CD57^+^ CD28^-^ CD27^-^ T_EMRA_-like cells, mirroring the corresponding antigen-experienced phenotype among the CD4 T-cells. This suggests a possible role for immune senescence in AHSCT-induced long-term tolerance.

### 4.7 MAIT cell dynamics

MAIT cells represent a unique subset of T cells characterized by the expression of the semi-invariant TCR TRAV1-2 (Vα7.2) paired with a limited set of TCRβ chains, typically TRBV6 or TRBV20. MAIT cells are primarily found in mucosal tissues, liver, and blood, where they respond to microbial vitamin B metabolites presented by MR1, triggering cytokine release and cytotoxic activity (45). Classical MAIT cells co-express CD161 and IL-18R, but a subset of Vα7.2^+^ T cells lack these markers, raising questions about whether these cells represent precursors to MAIT cells, a resting/naïve MAIT state, or an entirely distinct cell type. In MS, MAIT cells exhibit altered functionality and frequency, with their presence in MS lesions suggesting a potential role in MS pathology (46, 47).

Several studies have reported a persistent decline in MAIT cells following AHSCT in MS (38, 48, 49). In the present study, we observed a significant reduction in CD8 and double-negative (DN) MAIT cells at all time points up to two years post-transplant, with a slight recovery at later time points. In contrast, TcR Vα7.2^+^ T cells lacking classical MAIT markers (CD161^-^/IL-18R^-^) exhibited little variation from baseline. Whether these cells represent precursors to classical MAIT cells remains debated, and a more precise approach to defining their phenotype would involve the use of an MR1 tetramer loaded with an appropriate antigen. Additionally, given the frequent administration of antibiotics post-AHSCT, which alters the gut microbiota, it is possible that the reduction in classical MAIT cells reflects a decreased availability of bacterial-derived antigens necessary for their maintenance.

### 4.8 Limitations and future considerations

While our study provides valuable insights into immune reconstitution following AHSCT, several limitations should be acknowledged. The relatively small cohort size and observational design limit the generalizability of our findings, particularly regarding the subgroup experiencing post-AHSCT relapses. Consequently, these results should be considered exploratory and validated in larger cohorts. Moreover, our analyses were limited to circulating immune cell phenotypes, potentially influenced by external pathogens and immune events unrelated to MS.

Due to resource constraints and methodological scope, we did not include functional assays or B-cell receptor sequencing, which could provide deeper mechanistic insights. Specifically, detailed phenotypic and functional characterization of the unswitched IgD^+^IgM^+^ memory B-cell subset, including activation or exhaustion markers, somatic hypermutation status, and antibody secretion, represents an important direction for future research.

Future studies could also leverage single-cell sequencing and proteomics analyses of blood and cerebrospinal fluid (CSF) to further dissect molecular mechanisms driving immune reconstitution and relapse susceptibility.

## 5 Conclusion

Our findings underscore the potential of AHSCT to profoundly reset the immune system in RRMS, selectively depleting pathogenic Th1/Th17 CD4 T cells and expanding regulatory or exhausted PD-1^+^ subsets in CD4 and CD8 T cells. The observed expansion of atypical CD62L^-^ naïve CD4 T cells post-AHSCT may represent a novel biomarker reflecting immune activation or regulatory processes.

Future studies should investigate these atypical naïve CD4 T cells, PD-1^+^ T-cell subsets, CD4/CD8 ratios, and unswitched IgD^+^IgM^+^ memory B cells to determine their utility as predictive biomarkers for treatment response and relapse. Further mechanistic studies incorporating functional analyses would also enhance understanding of immune dynamics post-AHSCT.

In conclusion, this study reinforces AHSCT as a transformative therapy capable of inducing sustained immunological changes in MS, providing a foundation for future biomarker discovery, therapeutic monitoring, and improved patient stratification.

## Data Availability

The original contributions presented in the study are included in the article/**Supplementary Material**. Further inquiries can be directed to the corresponding author.

## Supplementary material

Supplementary Table S1 Patient Characteristics, Treatments, and Post-AHSCT Outcomes. Demographic, clinical data and blood sampling time points for CyTOF and flow cytometry analysis of AHSCT treated multiple sclerosis (MS) patients. Abbreviations: EDSS: Expanded Disability Status Scale scores. AUB: Aubagio, DMF: Dimethyl fumarate, FLM : Fingolimod, GLA: Glatiramer acetate, IFN: Interferon beta, IVIG: Intravenous immunoglobulin, MTX: Mitoxantrone NTZ: Natalizumab , RTX: Rituximab. Summary statistics (medians and ranges) are provided at the bottom.

Supplementary Table S2 Antibodies for Flow Cytometric analysis. The table summarizing antibodies and viability stains used for flow cytometric phenotypic analysis of T cells (panel 1-5). n/a; not applicable.
