# Supplementary material for: Longitudinal immune profiling following autologous hematopoietic stem cell transplantation in multiple sclerosis: insights into immune reconstitution and disease modulation
Source: Front Immunol. 2025 Jun 30;16:1601223. doi: 10.3389/fimmu.2025.1601223 (PMC12258046; doi:10.3389/fimmu.2025.1601223)
Supplement: Supplementary file 3 [file DataSheet1.pdf]

Figure S1.1

A

Antibodies used for CD45^{+} leukocyte analysis (panel 1)

Tag	Ab	Clone	Company
209Bi	CD11b/Mac-1	ICRF44	Fluidigm
147Sm	CD11c	Bu15	Fluidigm
151Eu	CD123	6H6	Biolegend
165Ho	CD127	A019D5	Fluidigm
160Gd	CD14	M5E2	Biolegend
144Nd	CD16	3G8	Biolegend
142Nd	CD19	HIB19	Fluidigm
170Er	CD20	2H7	Biolegend
152Sm	CD21	BL13	Fluidigm
155Gd	CD22	HIB22	Biolegend
161Dy	CD24	ML5	Biolegend
167Er	CD27	L128	Fluidigm
168Er	CD38	HIT2	Biolegend
173Yb	CD39	A1	BioLegend
154Sm	CD3e	UCHT1	Fluidigm
164Dy	CD44	BJ18	BioLegend
89Y	CD45	HI30	Fluidigm
169Tm	CD45RA	HI100	Fluidigm
143Nd	CD5	UCHT2	Biolegend
115In	CD57	HCD57	BioLegend
157Gd	CXCR3	G025H7	BioLegend
174Yb	CD185/CXCR5	51505	R&D
163Dy	HLA-DR	L243	BioLegend
159Tb	IgA	G18-1	BD
172Yb	IgD	IA6-2	Biolegend
146Nd	IgM	MHM-88	Biolegend

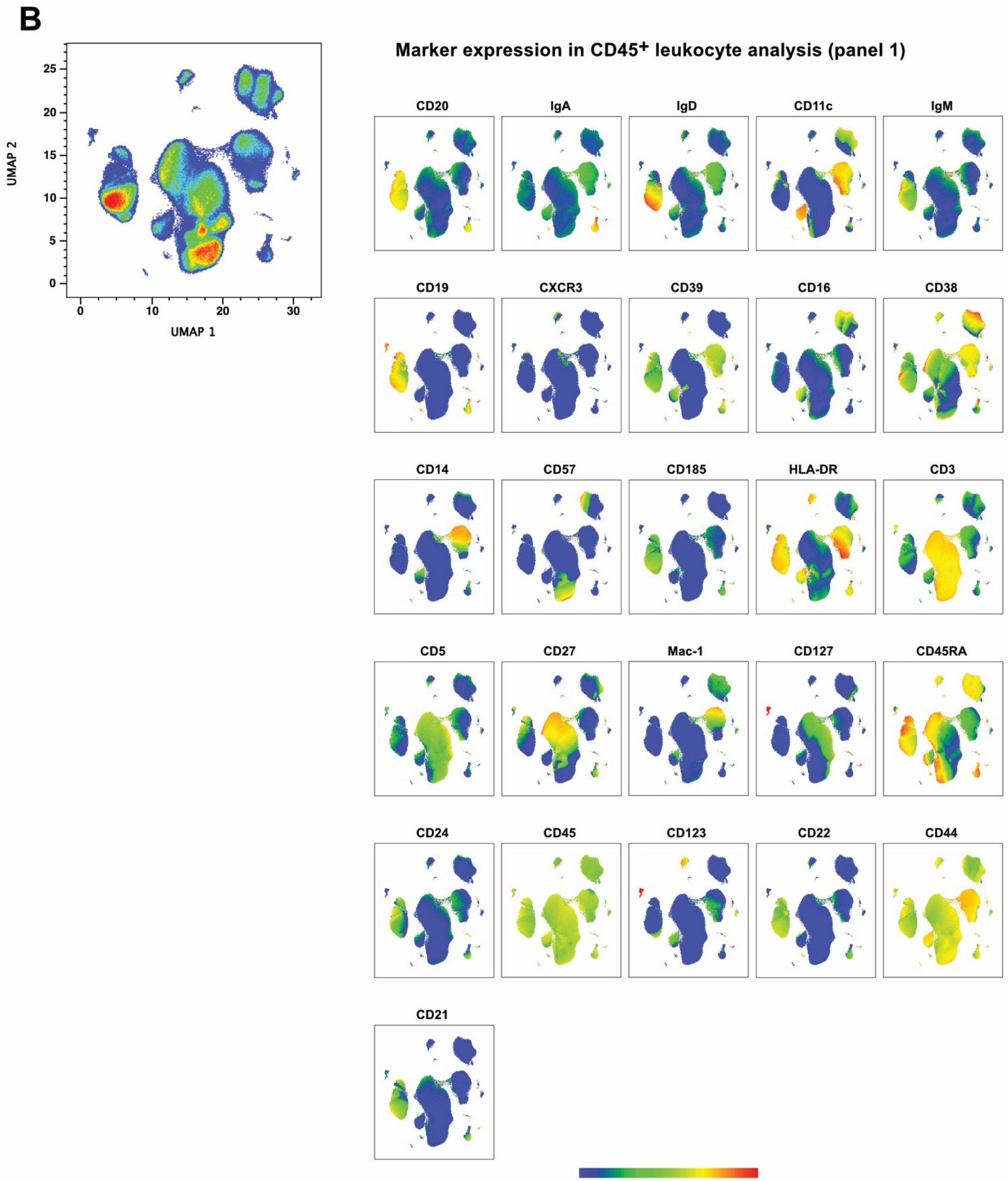

Figure S1.1: Table of antibodies used in CyTOF panel 1 for phenotypic analysis of the major cell types of CD45+ leukocytes and analysis of B cell and myeloid sub sets (**A**). Overview, by UMAP mapping, of marker expression of the antibodies in panel 1 (**B**).

Figure S1.2

A

Antibodies used for CD3 T cell analysis (panel 2)

Tag	Ab	Clone	Company
159Tb	CCR5	J418F1	Biolegend
165Ho	CD127	A019D5	Fluidigm
161Dy	CD161	HP-3G10	BioLegend
142Nd	CD19	HIB19	Fluidigm
155Gd	CD194/CCR4	205410	R&D
167Er	CD27	L128	Fluidigm
160Gd	CD28	CD28.2	BioLegend
148Nd	CD31	WM59	BioLegend
151Eu	CD38	HIT2	BioLegend
173Yb	CD39	A1	BioLegend
154Sm	CD3e	UCHT1	Fluidigm
145Nd	CD4	RPA-T4	Biolegend
164Dy	CD44	BJ18	BioLegend
89Y	CD45	HI30	Fluidigm
169Tm	CD45RA	HI100	Fluidigm
143Nd	CD5	UCHT2	Biolegend
115In	CD57	HCD57	BioLegend
146Nd	CD8a	SK1	Biolegend
170Er	CTLA-4	14D3	eBio
152Sm	TcRγδ	5A6.E9	Fischer S
163Dy	HLA-DR	L243	BioLegend
168Er	ICOS	C398.4A	Fluidigm
162Dy	Ki-67	B56	BioLegend
172Yb	PD-1	EH12.2H7	BioLegend

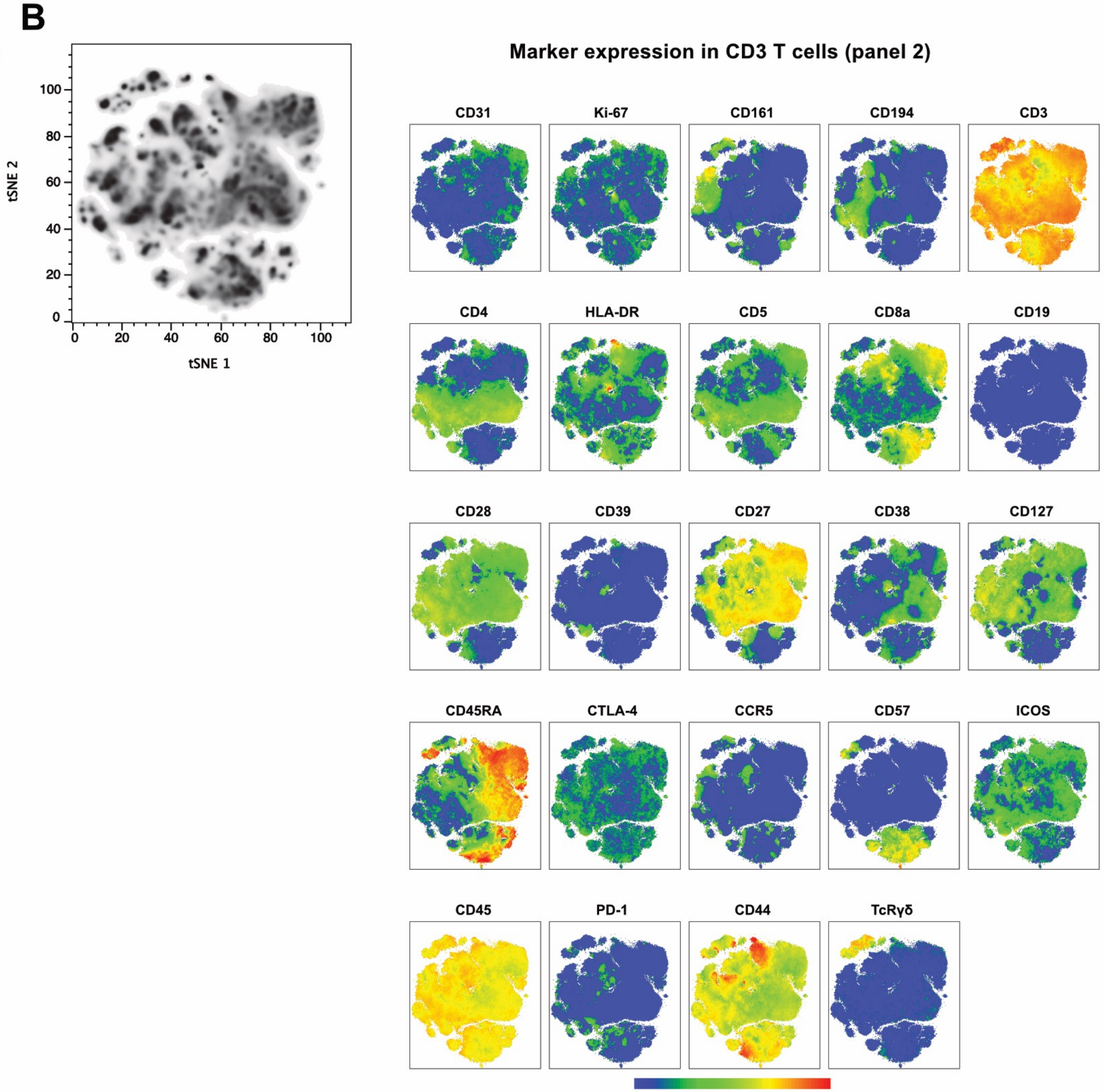

Figure S1.2: Table of antibodies used in CyTOF panel 2 for phenotypic analysis of T cells (**A**). Overview, by UMAP mapping, of marker expression of the antibodies in panel 2 (**B**).

Figure S1.3

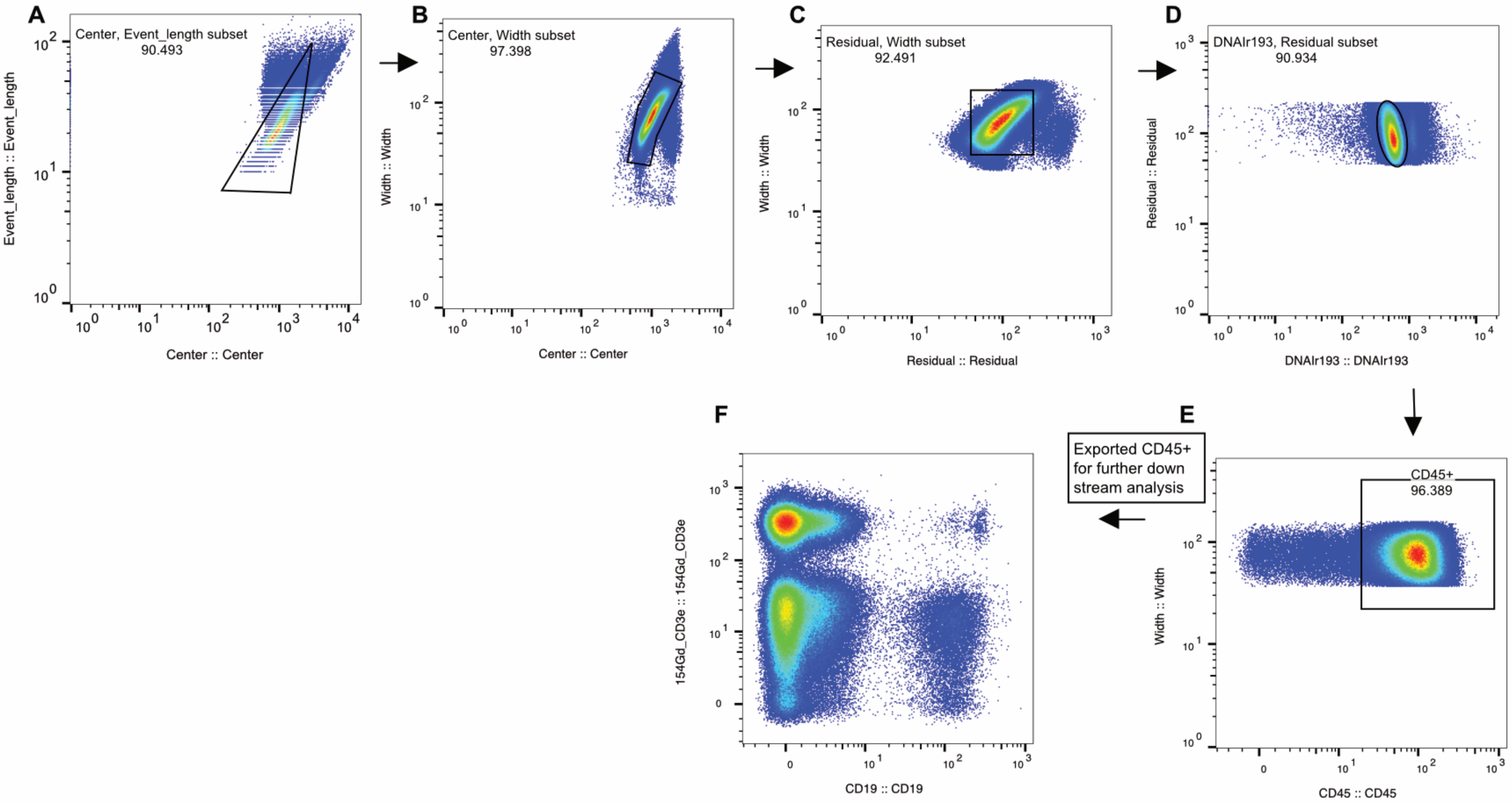

Figure S1.3: The plots show initial clean up gating of CyTOF data for both panel 1 and 2. Arrows indicate gating flow direction (**A**-F).

Figure S1.4

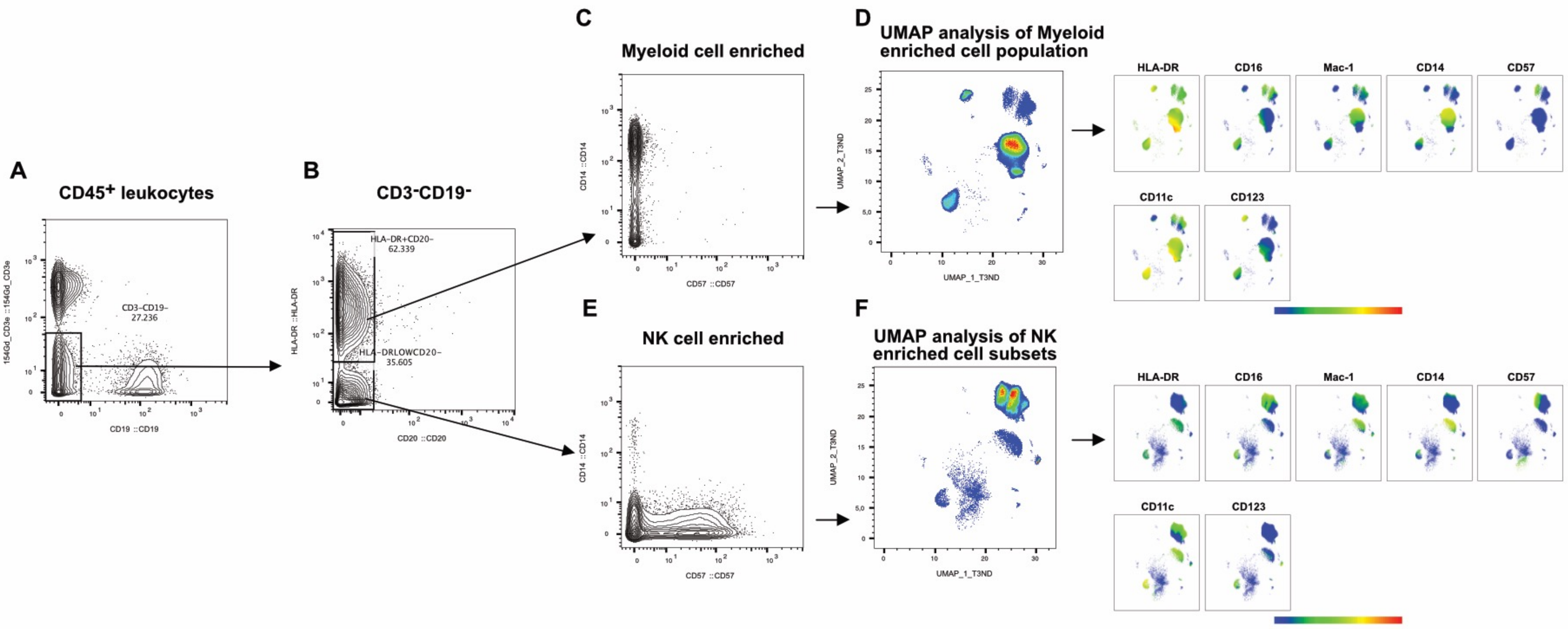

Figure S1.4: Gating strategy for Myeloid and NK cells in panel 1. Gate on CD3⁻CD19⁻ CD45⁺ cells (**A**), CD3⁻CD19⁻ CD45⁺split into HLA-DR⁺CD20⁻ (Myeloid enriched) and HLA-DR⁻ CD20⁻ (NK cell enriched) (**B**), CD14 and CD57 expression and UMAP overview of marker expression in Myeloid enriched cells (**C,D**) and NK cell enriched (**E,F**) Arrows indicate gating flow direction.

Figure S1.5

A

Antibodies used for myeloid cell analysis (panel 1)

Tag	Ab	Clone	Company
209Bi	CD11b/Mac-1	ICRF44	Fluidigm
147Sm	CD11c	Bu15	Fluidigm
151Eu	CD123	6H6	Biolegend
160Gd	CD14	M5E2	Biolegend
144Nd	CD16	3G8	Biolegend
168Er	CD38	HIT2	Biolegend
173Yb	CD39	A1	BioLegend
164Dy	CD44	BJ18	BioLegend
169Tm	CD45RA	HI100	Fluidigm
157Gd	CXCR3	G025H7	BioLegend
163Dy	HLA-DR	L243	BioLegend

B

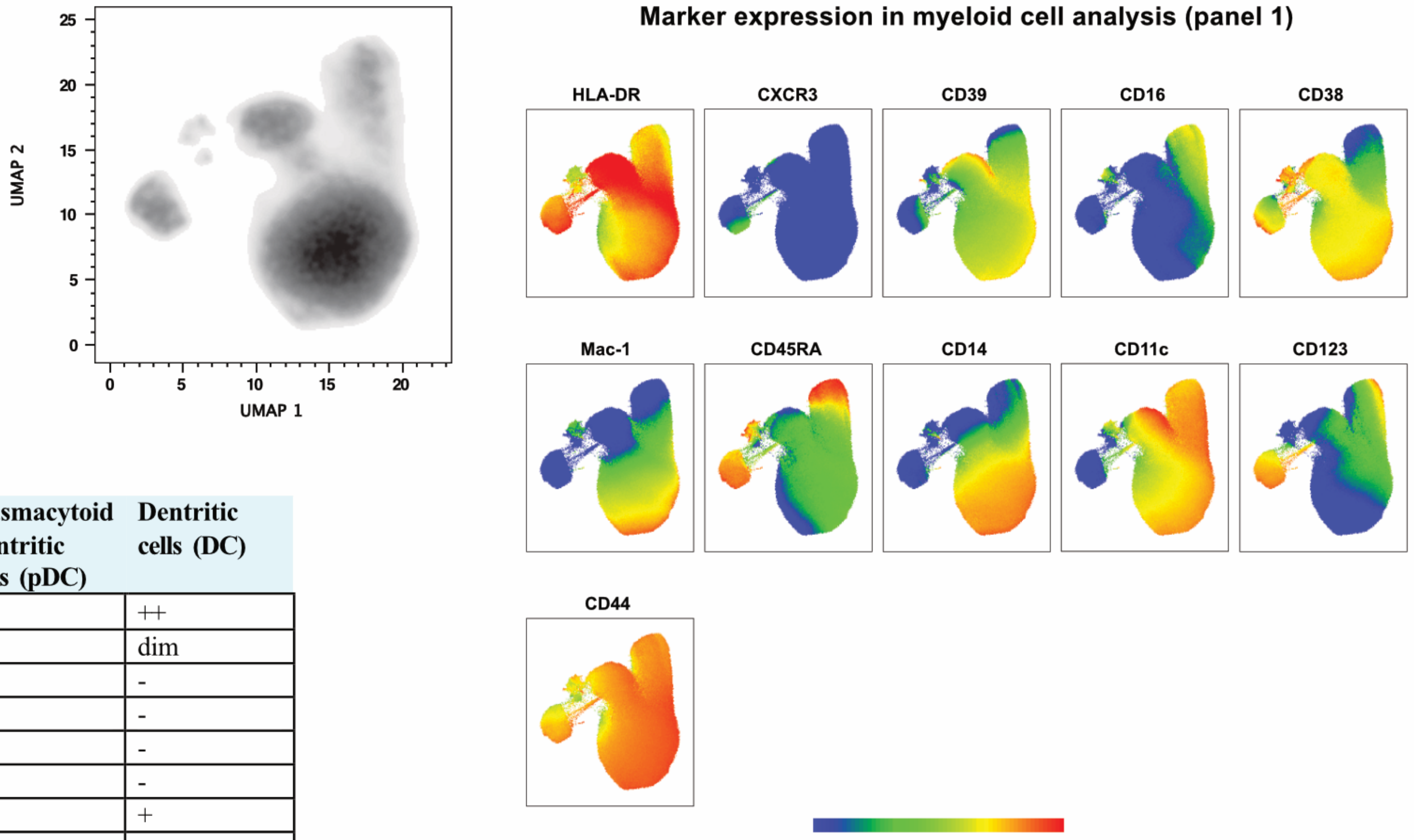

C

	Classical Monocyte	Intermediate Monocytes	Non-classical monocytes	Plasmacytoid Dentritic cells (pDC)	Dentritic cells (DC)
**HLA-DR**	++	++	++	++	++
**CD11c**	++	++	+	-	dim
**CD14**	++	+	-	-	-
**CD16**	-	dim	+	-	-
**Mac-1**	+	dim	-	-	-
**CD123**	-	low/-	dim	+	-
**CD44**	+	+	+	+	+
**CD45RA**	low/-	dim	+	+	+
**CD39**	+	+	low	-	dim/-
**CD38**	+	+	-	+	+
**CXCR3**	-	-	¨	+	-

Figure S1.5: Table of antibodies used in CyTOF panel 1 for phenotypic analysis of Myeloid cell sub sets(**A**). Overview, by UMAP mapping, of marker expression of the antibodies in panel 1 (**B**). Definition of myeloid cell types (**C**)

Figure S1.6

A

Antibodies used for B cell analysis (panel 1)

Tag	Ab	Clone	Company*
147Sm	CD11c	Bu15	Fluidigm
151Eu	CD123	6H6	Biolegend
142Nd	CD19	HIB19	Fluidigm
170Er	CD20	2H7	Biolegend
152Sm	CD21	BL13	Fluidigm
155Gd	CD22	HIB22	Biolegend
161Dy	CD24	ML5	Biolegend
167Er	CD27	L128	Fluidigm
168Er	CD38	HIT2	Biolegend
173Yb	CD39	A1	BioLegend
164Dy	CD44	BJ18	BioLegend
169Tm	CD45RA	HI100	Fluidigm
143Nd	CD5	UCHT2	Biolegend
174Yb	CXCR5/CD185	51505	R&D
163Dy	HLA-DR	L243	BioLegend
159Tb	IgA	G18-1	BD
172Yb	IgD	IA6-2	Biolegend
146Nd	IgM	MHM-88	Biolegend

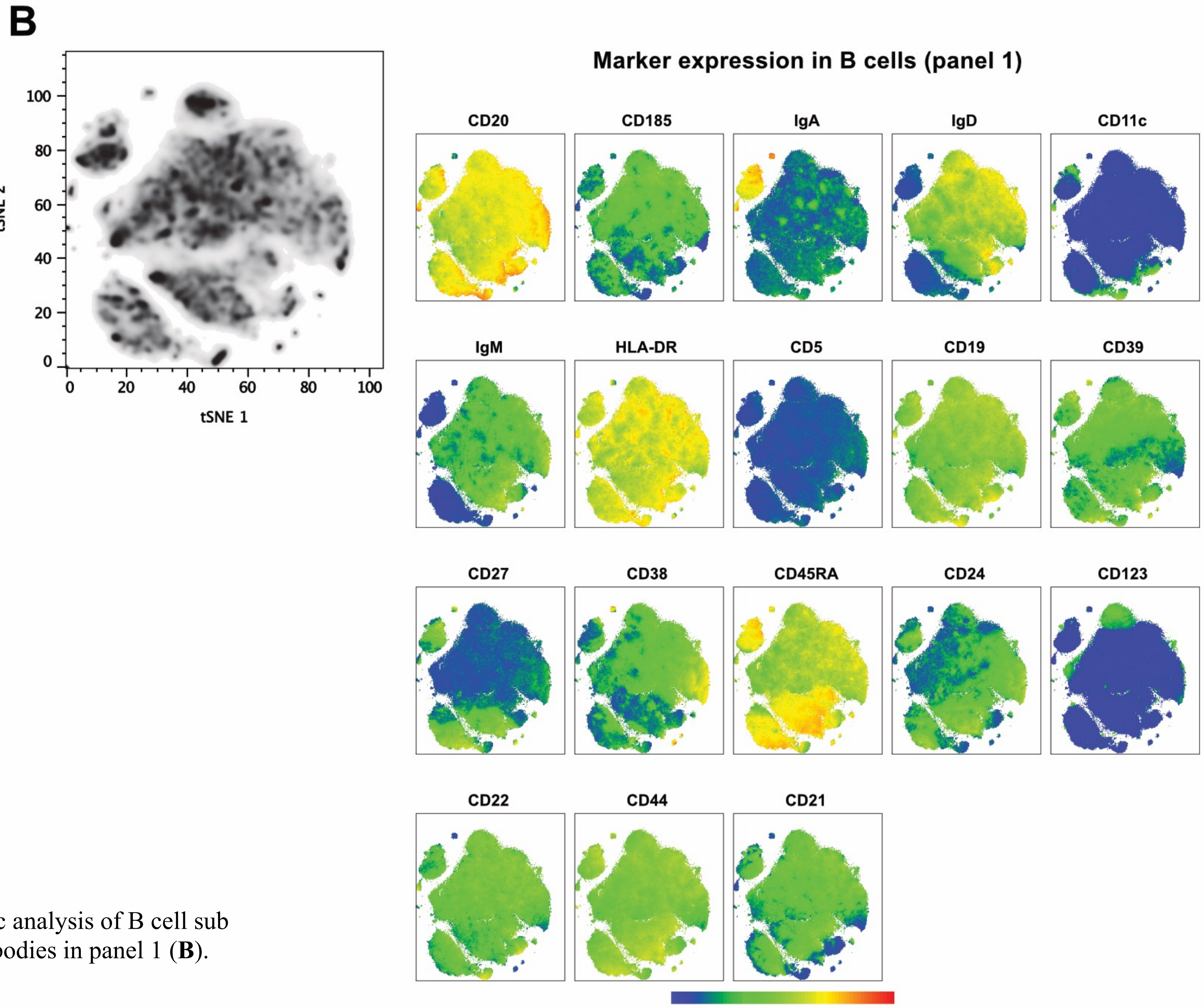

Figure S1.6: Table of antibodies used in CyTOF panel 1 for phenotypic analysis of B cell sub sets(**A**). Overview, by tSNE mapping, of marker expression of the antibodies in panel 1 (**B**).

Figure S1.7

A

Antibodies used for CD4 T cell analysis (panel 2)

Tag	Ab	Clone	Company
159Tb	CCR5	J418F1	Biolegend
165Ho	CD127	A019D5	Fluidigm
161Dy	CD161	HP-3G10	BioLegend
155Gd	CD194/CCR4	205410	R&D
167Er	CD27	L128	Fluidigm
160Gd	CD28	CD28.2	BioLegend
148Nd	CD31	WM59	BioLegend
151Eu	CD38	HIT2	BioLegend
173Yb	CD39	A1	BioLegend
164Dy	CD44	BJ18	BioLegend
169TmA	CD45RA	HI100	Fluidigm
143Nd	CD5	UCHT2	Biolegend
115In	CD57	HCD57	BioLegend
170Er	CTLA-4	14D3	eBio
163Dy	HLA-DR	L243	BioLegend
168Er	ICOS	C398.4A	Fluidigm
162Dy	Ki-67	B56	BioLegend
172Yb	PD-1	EH12.2H7	BioLegend

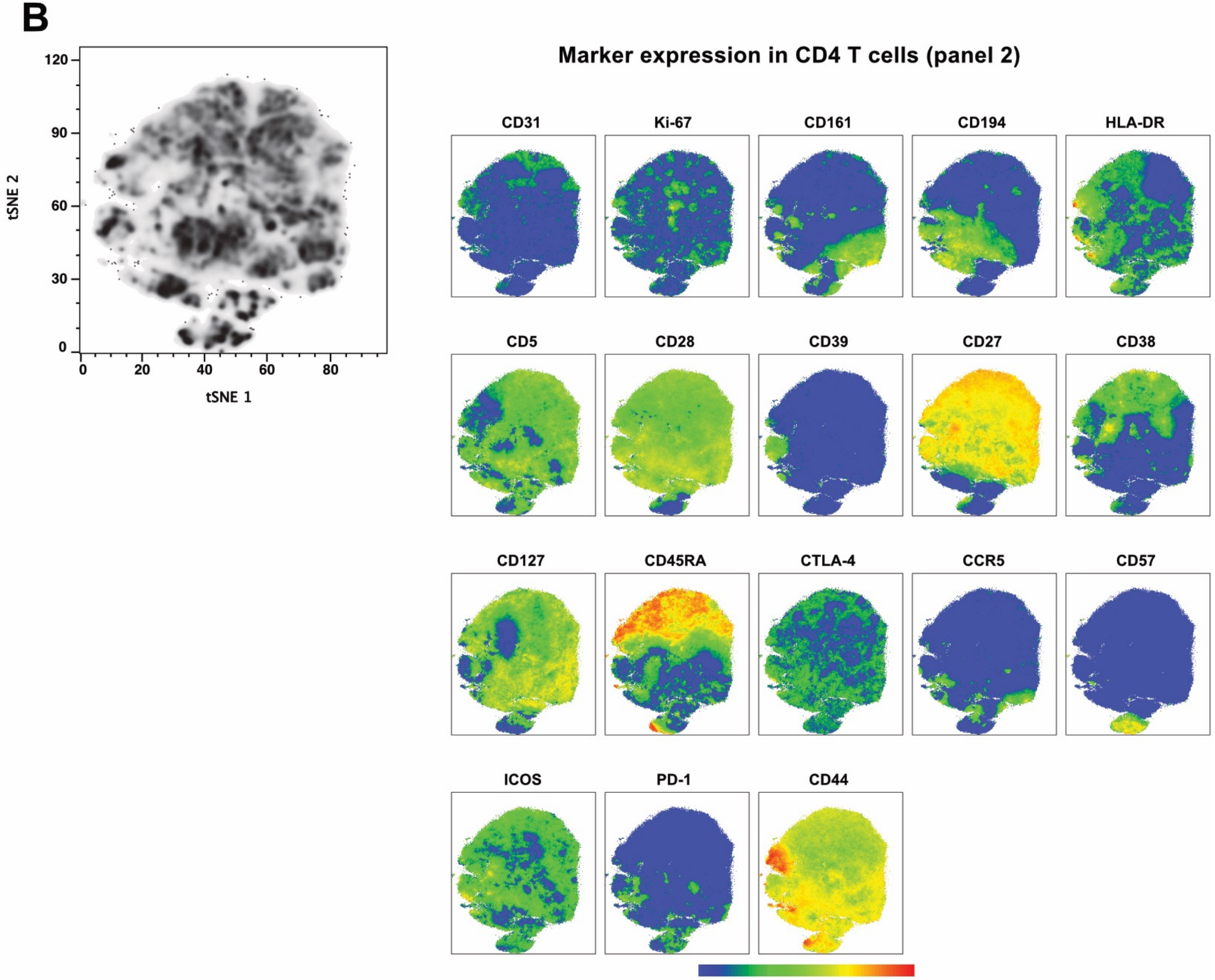

Figure S1.7: Table of antibodies used in CyTOF panel 2 for phenotypic analysis of CD4 T cell sub sets(**A**). Overview, by tSNE mapping, of marker expression of the antibodies in panel 2 (**B**).

Figure S1.8

A

Antibodies used for CD8 T cell analysis (panel 2)

Tag	Ab	Clone	Company
159Tb	CCR5	J418F1	Biolegend
165Ho	CD127	A019D5	Fluidigm
161Dy	CD161	HP-3G10	BioLegend
155Gd	CD194/CCR4	205410	R&D
167Er	CD27	L128	Fluidigm
160Gd	CD28	CD28.2	BioLegend
148Nd	CD31	WM59	BioLegend
151Eu	CD38	HIT2	BioLegend
173Yb	CD39	A1	BioLegend
164Dy	CD44	BJ18	BioLegend
169Tm	CD45RA	HI100	Fluidigm
143Nd	CD5	UCHT2	Biolegend
115In	CD57	HCD57	BioLegend
163Dy	HLA-DR	L243	BioLegend
168Er	ICOS	C398.4A	Fluidigm
162Dy	Ki-67	B56	BioLegend
172Yb	PD-1	EH12.2H7	BioLegend
152Sm	TcRγδ	5A6.E9	Fischer S

B

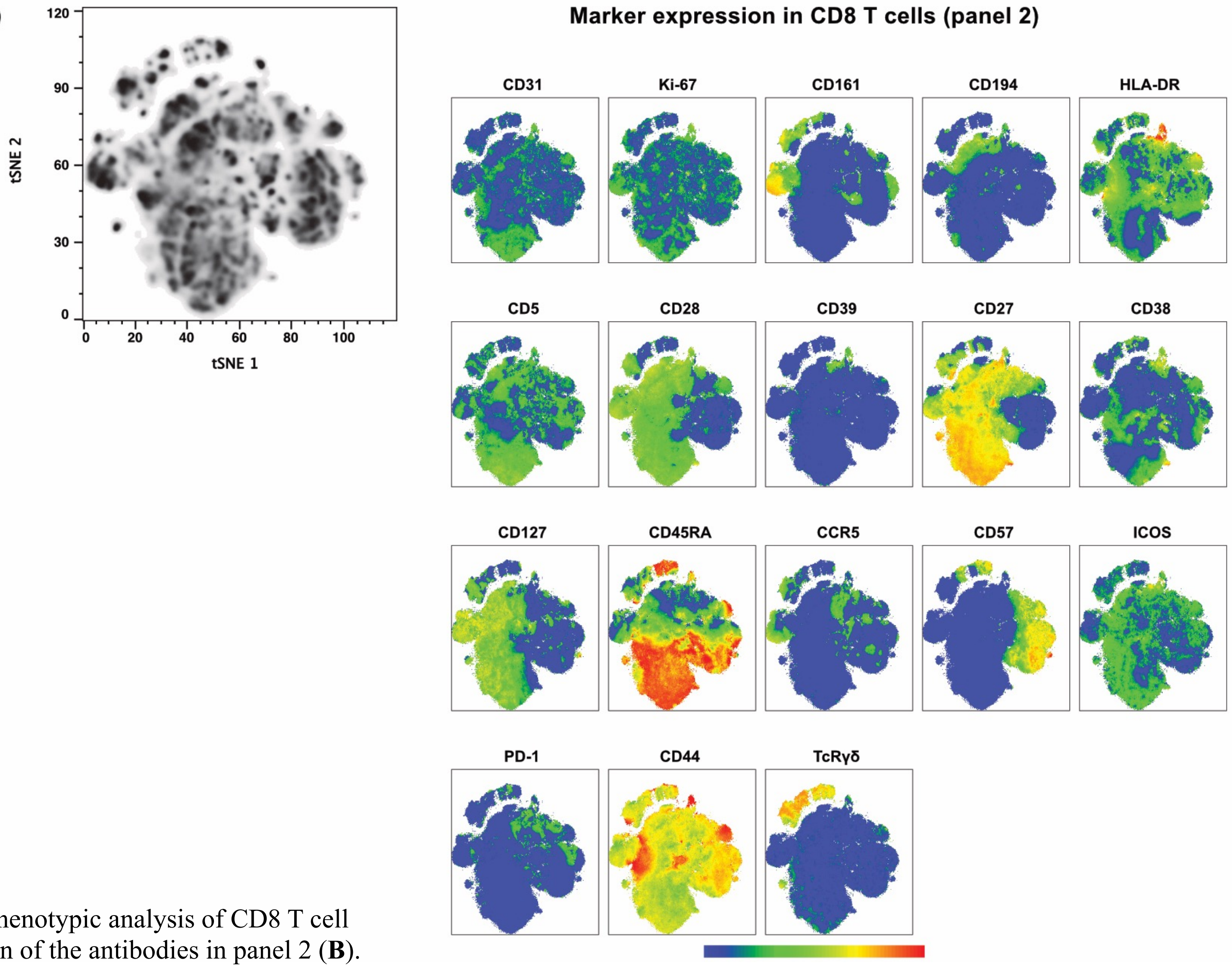

Figure S1.8: Table of antibodies used in CyTOF panel 2 for phenotypic analysis of CD8 T cell sub sets(**A**). Overview, by tSNE mapping, of marker expression of the antibodies in panel 2 (**B**).

Figure S1.9

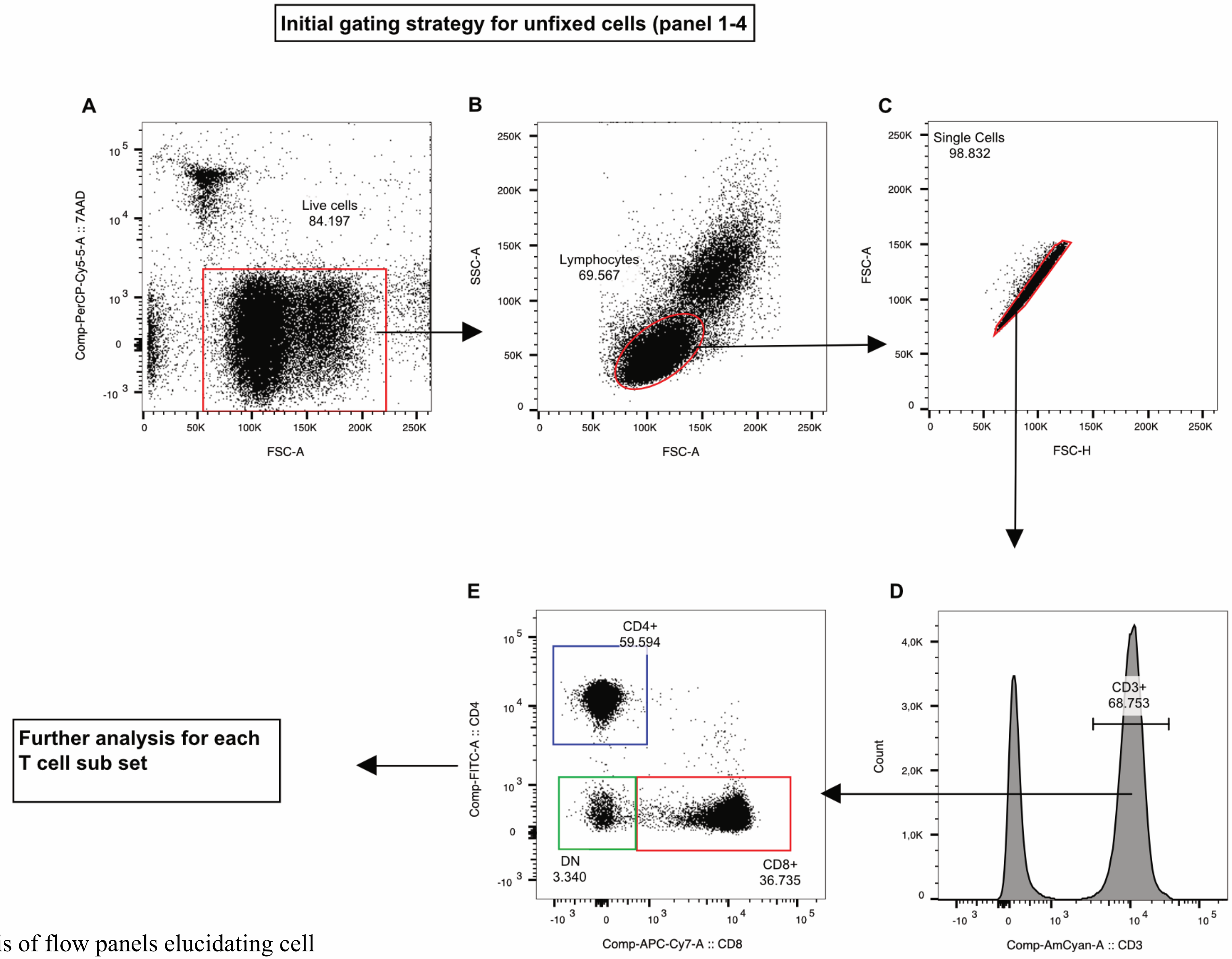

Figure S1.9: Initial gating strategy for flow cytometric analysis of flow panels elucidating cell surface expressed antigens (panel 1-4). Arrows indicate gating flow direction.

Figure S1.10

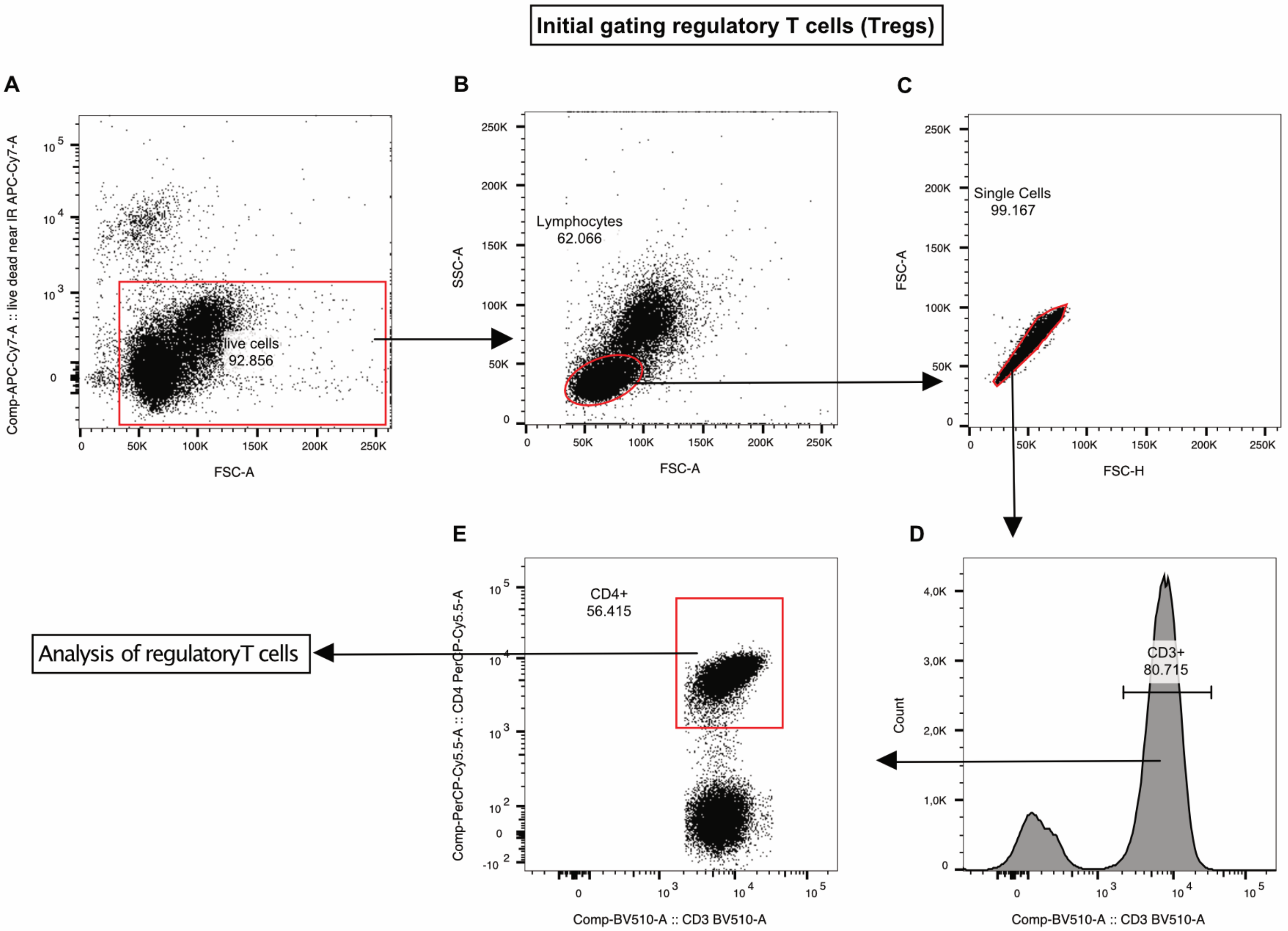

Figure S1.10: Initial gating strategy of flow cytometric analysis of fixated and permeabilized cells elucidating the nuclear expressed antigens FoxP3 and Helios (panel 5). Arrows indicate gating flow direction.

Figure S1.11

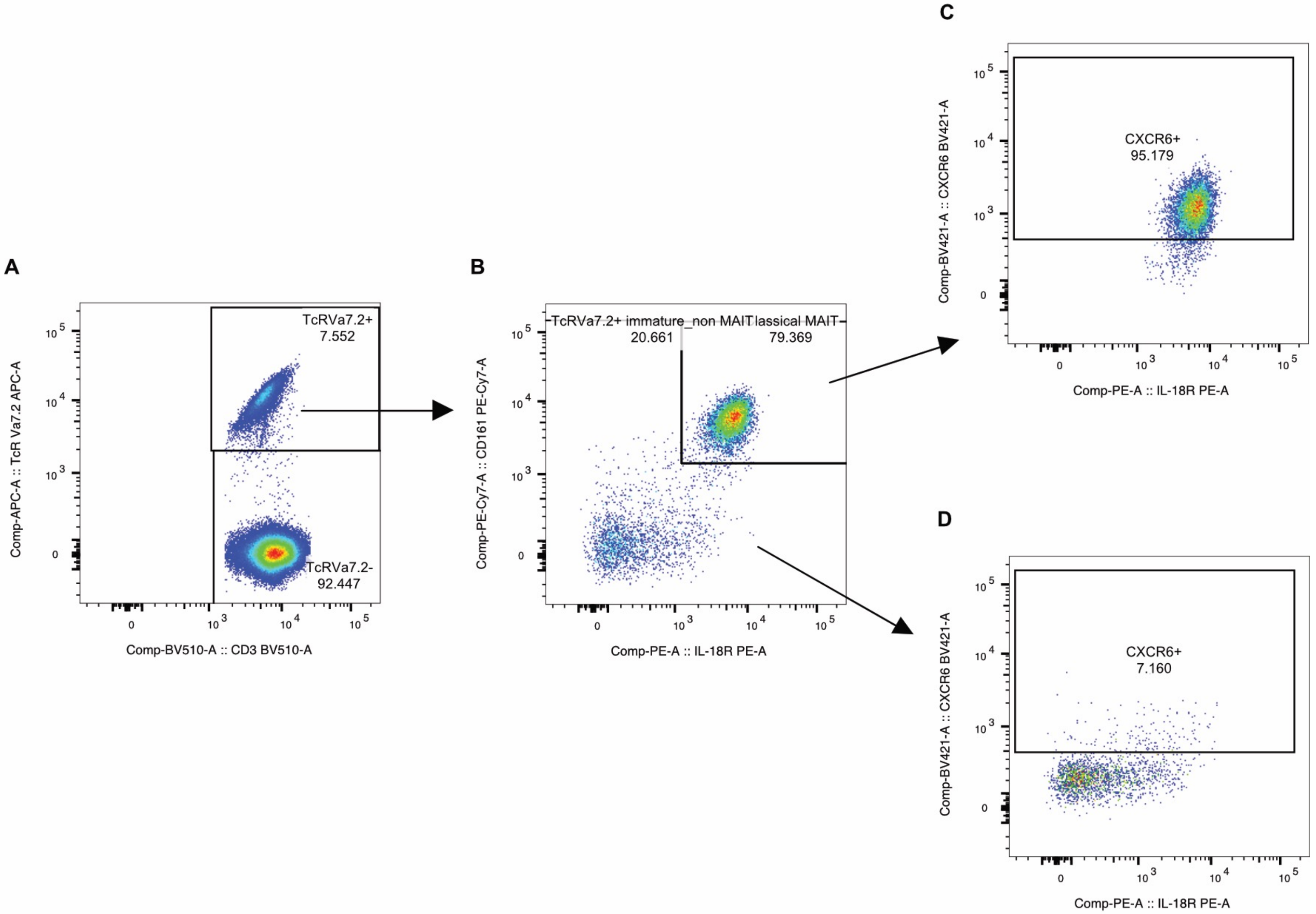

Figure S1.11: Gating strategy flow for MAIT cells. The dot plots show MAIT cells gaited on live $CD3^{+}$ T lymphocytes but the same gaiting approach was applied on T cell subsets i.e.CD8, CD4 and double negative (DN). Arrows indicate gating flow direction.

Figure S1.12

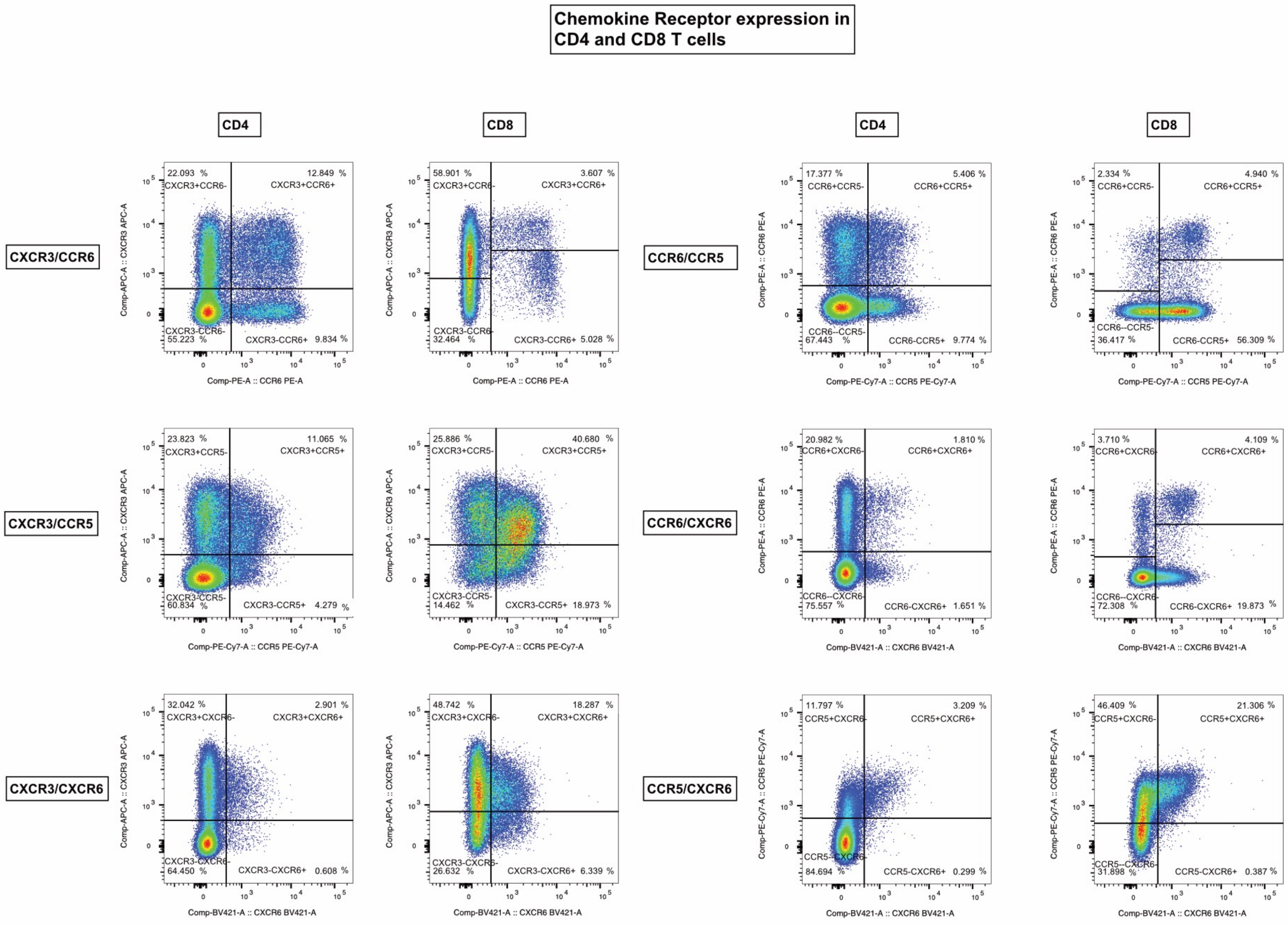

Figure S1.12: The dot plots show the staining patterns and gating approach of the chemokine receptors CXCR3, CCR6, CCR5 and CXCR6, in all possible combinations on CD4 and CD8 T cells.

Figure S1.13

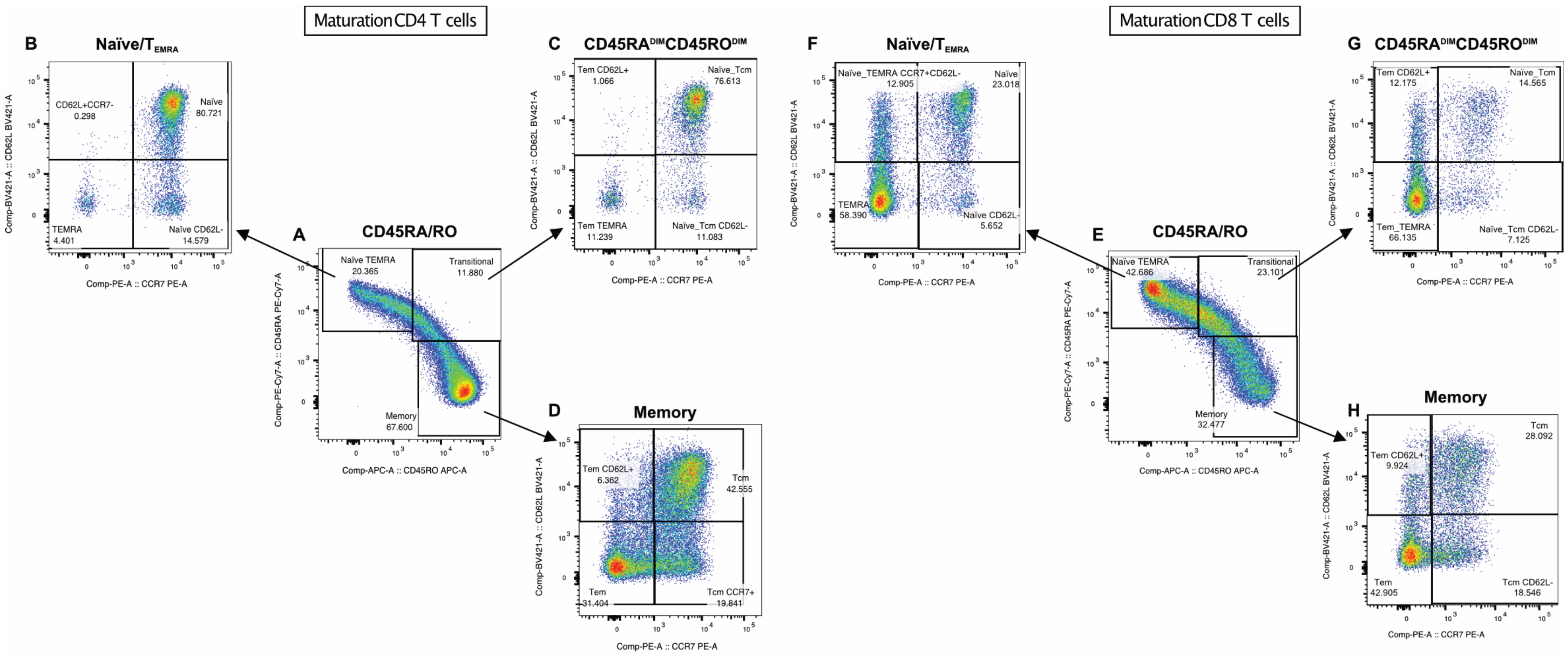

Figure S1.13 : Gating strategy of maturation phenotype in CD4 T cells using CD45RA, CD45RO, CD62L and CCR7. CD4$^+$CD45RA/CD45RO expression pattern. (**A**) CD4$^+$Naïve/T$_{EMRA}$ sub sets of CD45RA$^+$CD45RO$^-$ (**B**), CD4$^+$ Naïve/T$_{cm}$, T$_{em}$ sub sets of CD45RA^{dim}CD45RO^{dim} (**C**) CD4$^+$ memory T cells sub sets of CD45RA$^-$CD45RO$^+$ (**D**). CD8$^+$CD45RA/CD45RO expression pattern. (**E**) CD8$^+$Naïve/T$_{EMRA}$ sub sets of CD45RA$^+$CD45RO$^-$ (**F**), CD8$^+$ Naïve/T$_{cm}$, T$_{em}$ sub sets of CD45RA^{dim}CD45RO^{dim} (**G**) CD4$^+$ memory T cells sub sets of CD45RA$^-$CD45RO$^+$ (**H**). Arrows indicate gating flow direction.

Figure S1.14

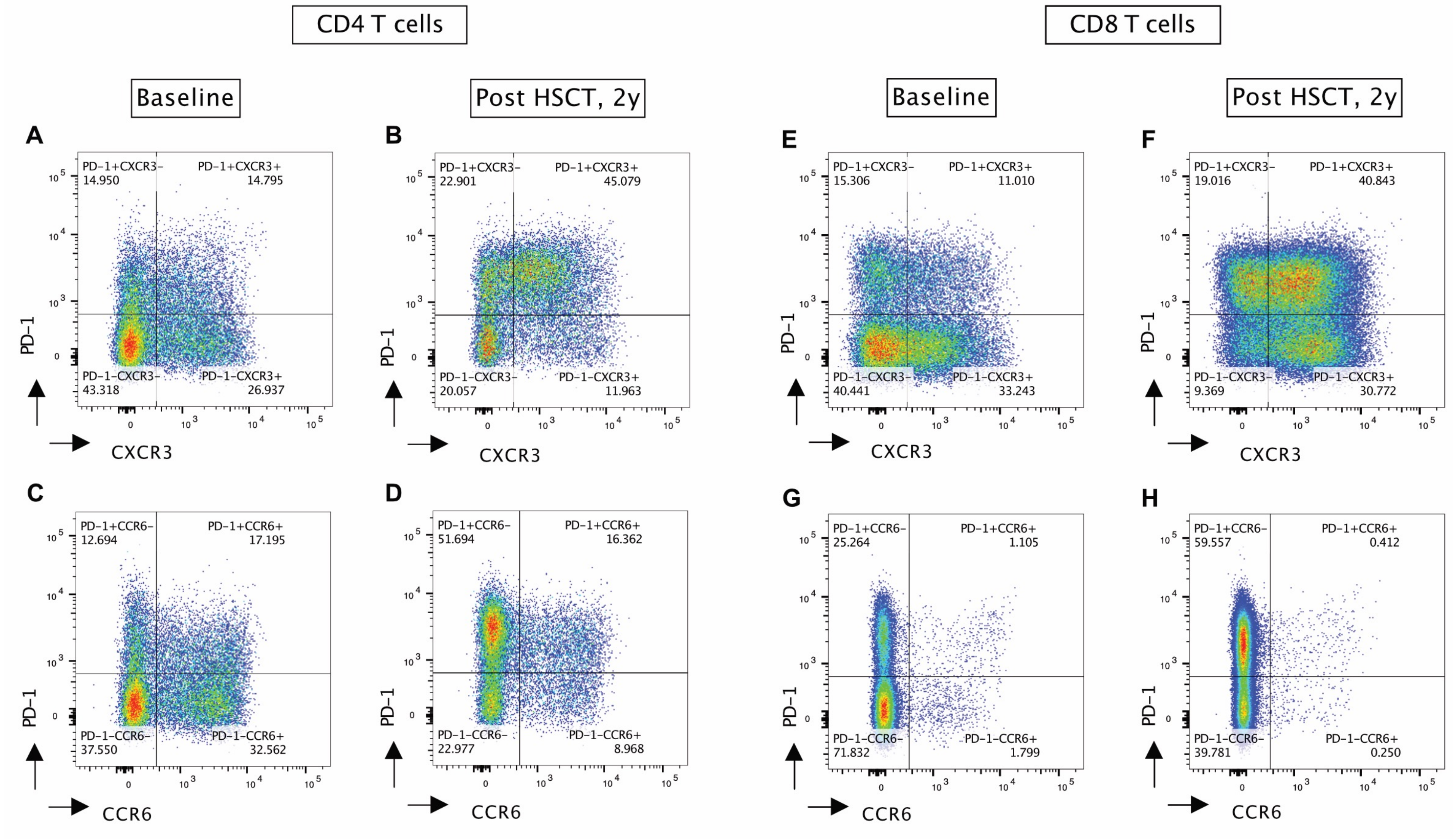

Figure S1.14: Co-expression of PD-1 and CXCR3 and PD-1 and CCR6 at baseline and two years post ASCT

Figure S1.15

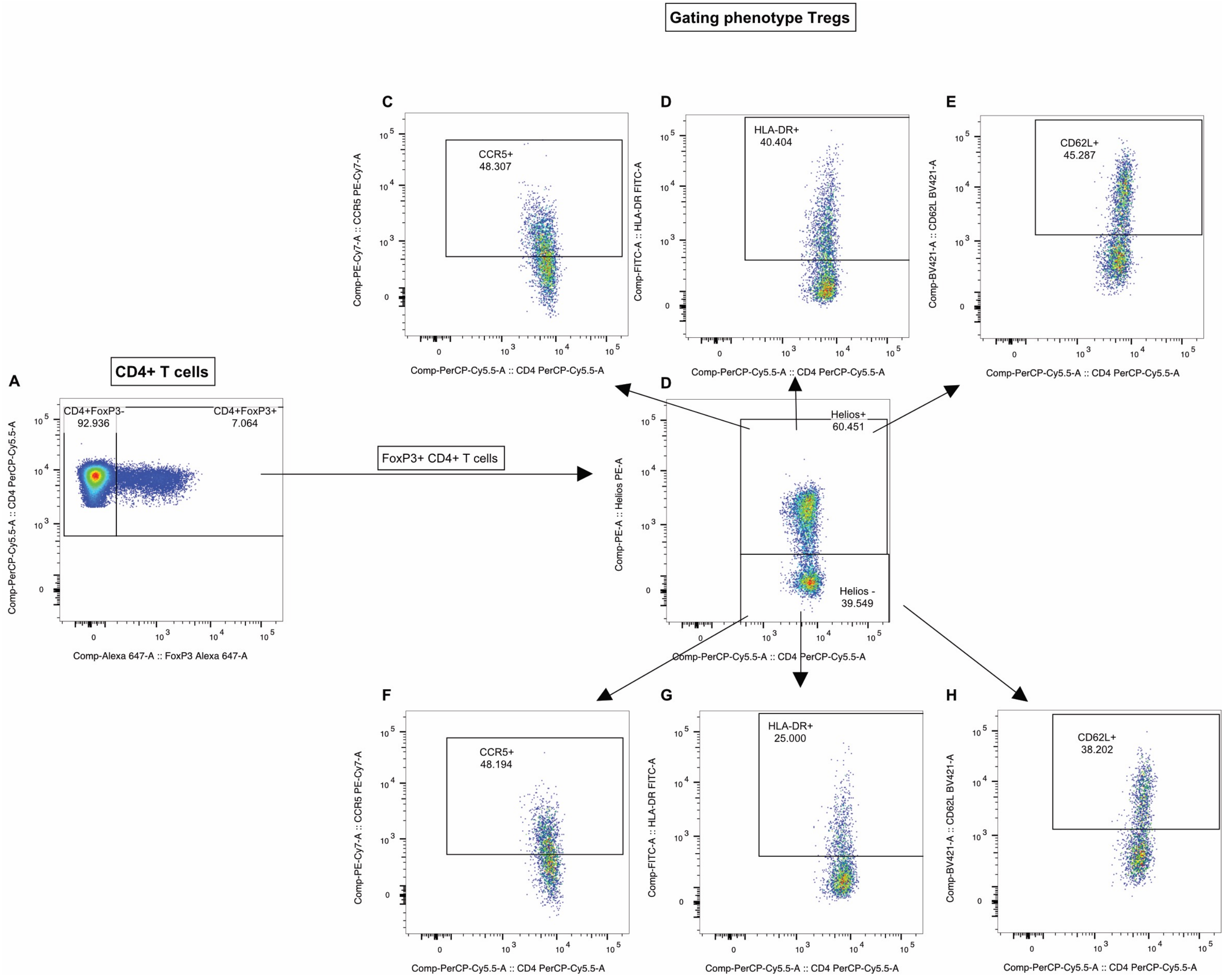

Figure S1.15: Representative plots of gating strategies of flow cytometric phenotypic analysis of FoxP3^{+} regulatory T cells (Treg). (**A**) FoxP3 expression in CD4^{+} T cells. (**B**) FoxP3^{+} CD4^{+} T cells divided into Helios^{+} and Helios^{-} FoxP3^{+} CD4^{+} T cells (nTreg and iTreg respectively). Expression on nTreg (Helios^{+} FoxP3^{+}) of (**C**) CCR5, (**D**) HLA-DR and (**E**) CD62L. Expression on iTreg (Helios^{-} FoxP3^{+}) of (**F**) CCR5, (**G**) HLA-DR and (**H**) CD62L. Arrows indicate gating flow direction.

Figure S1.16

T Helper Subset	Surface Markers	Transcription Factors	Typical cytokines Produced	Primary Functions	Tissue Homing & Disease
**Th1**	**CXCR3⁺** **CCR5⁺** IL12R⁺ **CCR6⁻** ***CD161⁻***	T-bet	IFN-γ, TNF-α, IL-2	Intracellular pathogen defense, macrophage-activation	**Homing:** Inflamed tissues (CNS, joints, gut) **Diseases:**MS, RA, Crohn's, Type 1 Diabetes
**Th1/Th17 (Th17.1)**	**CXCR3⁺** **CCR6⁺** ***CD161⁺/⁻*** ***CCR4⁻/dim***	T-bet, RORγt	IFN-γ, IL-17A, GM-CSF	Hybrid inflammatory T cell, highly pathogenic in autoimmunity	**Homing:** CNS, joints, inflam tissues **Diseases:**MS, RA, Crohn's
**Th17**	**CCR6⁺** ***CD161⁺*** ***CCR4⁺/⁻*** IL23R **CXCR3⁻**	RORγt, STAT3	IL-17A, IL-17F, IL-22, IL-21	Mucosal immunity, neutrophil-recruitmen	**Homing:** Skin, gut, CNS **Diseases:**MS, RA, Crohn,'s, Psoriasis
**Th2**	***CCR4⁺*** CRTH2⁺ CCR8⁺ CCR3⁺ ***CD161⁻***	GATA3	IL-4, IL-5, IL-13	Defense against parasites, allergic responses, eosinophil recruitmen	**Homing:** Mucosal tissues (lungs, skin) **Diseases:**Asthm Allergies, Atopic Dermatitis

Figure S1.16: Definitions of CD4 T helper subsets**.**
