# Supplementary material for: Longitudinal immune profiling following autologous hematopoietic stem cell transplantation in multiple sclerosis: insights into immune reconstitution and disease modulation
Source: Front Immunol. 2025 Jun 30;16:1601223. doi: 10.3389/fimmu.2025.1601223 (PMC12258046; doi:10.3389/fimmu.2025.1601223)
Supplement: Supplementary file 4 [file DataSheet2.pdf]

Figure S2.1

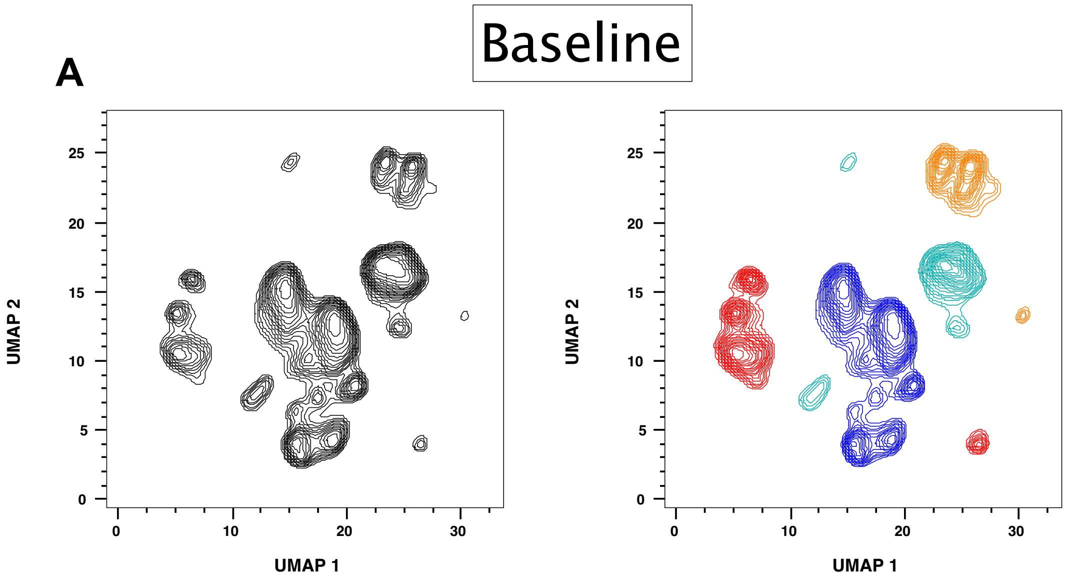

	Subset Name	Freq. of $CD45^{+}$PBMCs
	T cells	55.182
	B cells	10.295
	Myeloid cells	25.450
	NK cells	8.917

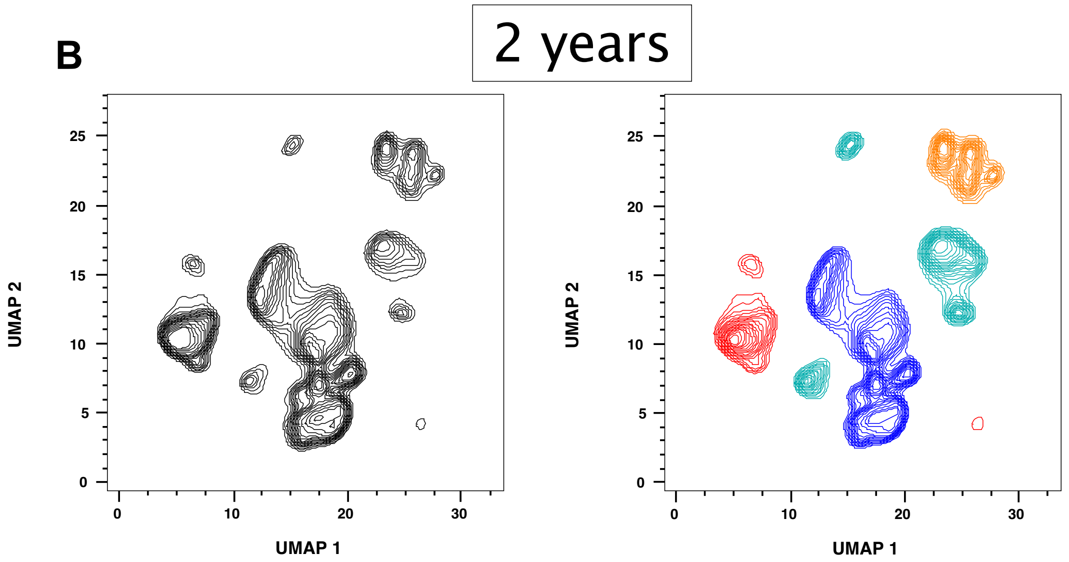

	Subset Name	Freq. of $CD45^{+}$PBMCs
	T cells	58.895
	B cells	20.473
	Myeloid cells	10.629
	NK cells	9.875

Figure S2.1: UMAP visualization of CytoF data showing an overview of the general cell type composition of $CD45^{+}$ leukocytes in PBMC from MS patients enrolled for HSCT, (**A**) at baseline (n=12) (**B**) and 2 years post HSCT (n=12) Definition of T cells, $CD3^{+}CD19^{-}CD14^{-}$, B cells $CD3^{-}CD19^{+}$ $CD20^{+/-}$, NK cells $CD3^{-}CD19^{-}CD14^{-}$ HLA-$^{DR-/dim}$ and Myeloid cells $CD3^{-}CD19^{-}CD20^{-}$HLA-$DR^{+/high}$.

Figure S2.2

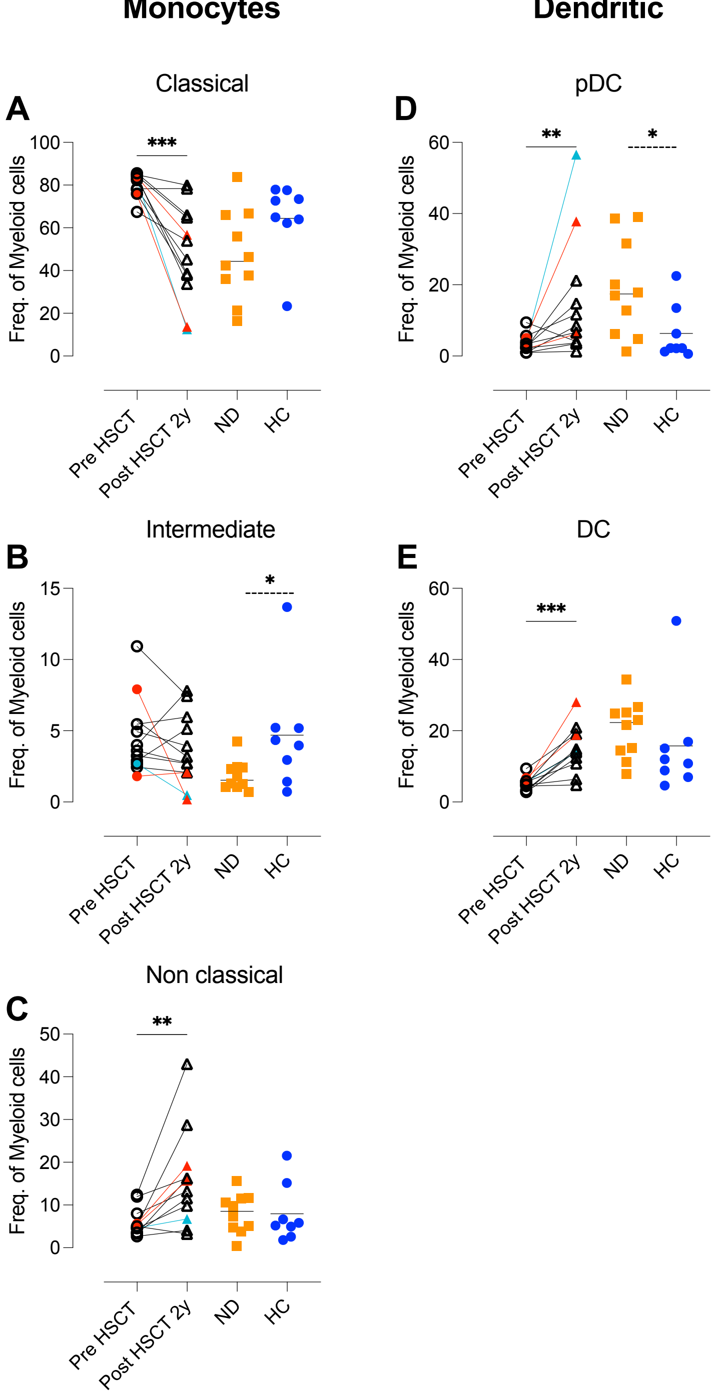

Figure S2.2: Cytometric (CyTOF) data of Myeloid cell subsets in PBMCs from MS patients pre (n=12) and two years post HSCT (n=12), newly diagnosed MS patients (ND) (n=10) and healthy controls (HC) (n=8).

The summary graphs depicting proportions of cells in cell cluster defined, by high dimensional reduction analysis (UMAP) followed by PhenoGraph clustering and cell cluster analysis by Cluster Explorer, as (**A**) Classical Monocytes (**B**) Intermediate Monocytes (**C**) Non-Classical Monocytes (**D**) Intermediate Monocytes (**E**) Plasmacytoid Dendritic cells pDC and (**F**). Frequencies are calculated out of $CD45^{+}CD3^{-}CD19^{-}CD20^{-}HLA\text{-}DR^{++}$ defined as Myeloid cells. Patients with post-HSCT relapse are marked in red, while the patient with a new T2 event on MRI (but in remission) is marked in turquoise. Statistical analyses: Wilcoxon matched-pair test (solid line, $**p<0.01$, $***p<0.001$) for paired samples and Mann-Whitney test (hatched line, $*p<0.05$, *) for unpaired groups.

Figure S2.3

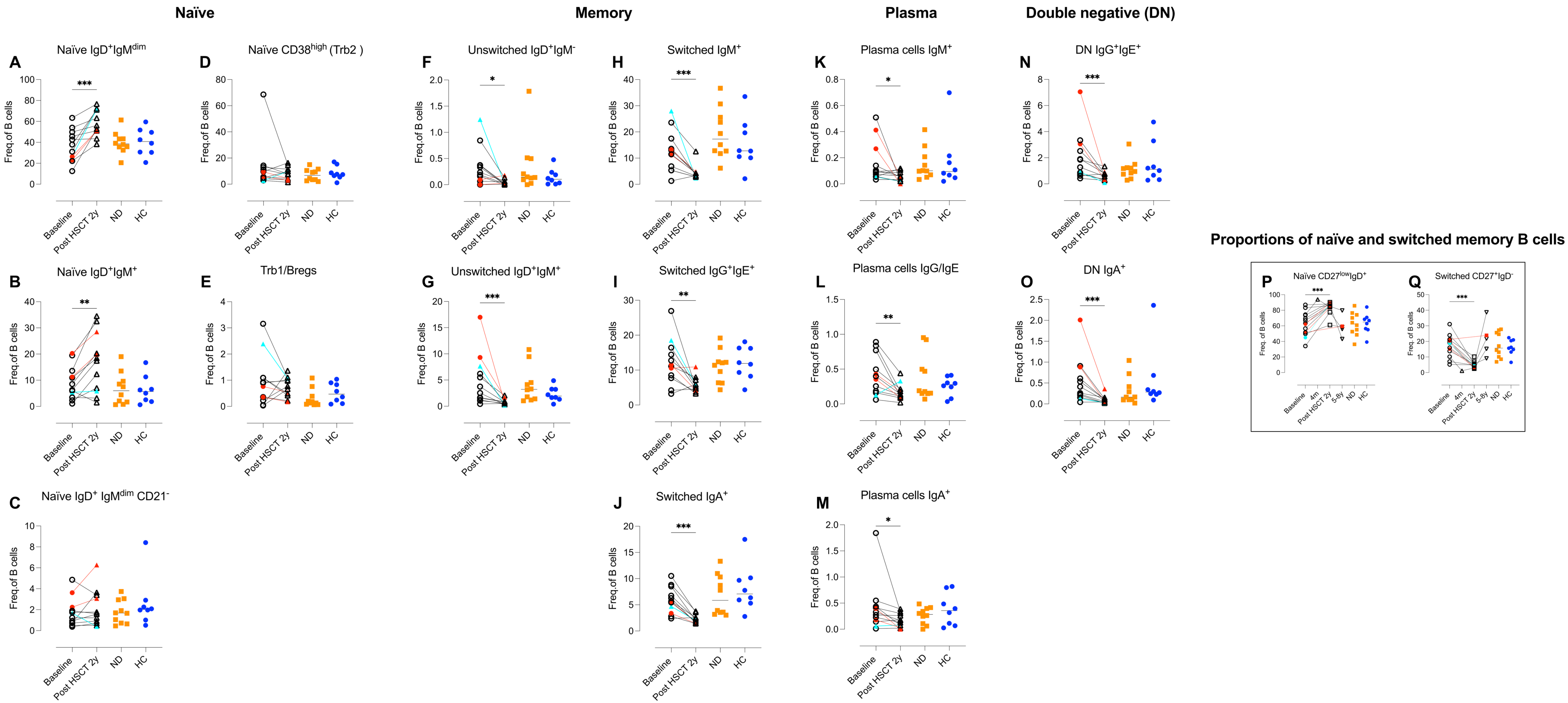

Figure S2.3: Summary graphs depicting the frequencies of B cells in each phenotypic distinct cell cluster (clusters A-O). Data is presented for each individual based on tSNE, PhenoGraph, and Cluster Explorer analysis of CyTOF data from **panel 1. Study subjects:** MS patients pre (n=12) and two years post HSCT (n=12), newly diagnosed MS patients (ND) (n=10), and healthy controls (HC) (n=8).Manually gated frequencies of Naïve ($CD3^{-}CD19^{+}CD27^{low}IgD^{+}$) (**P**) and Memory ($CD3^{-}$ $CD19^{+}CD27^{+}IgD^{-}$) (**Q**) B cells. Data presented is combined analysis of CyTOF data (panel 1) and additional timepoints post HSCT analyzed by Flow cytometry. Baseline (n=14), 4m (n=1), 2y (n=13) and 5-8y (n-5) as well as newly diagnosed (ND) (n=10) and healthy subjects (HC) (n=8) Patients with relapse post HSCT is marked with red and the patient with new T2 event at MRI but in clinical remission is marked with turquoise. **Statistical analysis**: Wilcoxon matched-pairs test (*p<0,05, **p<0,01, ***p<0,001)

Figure S2.4

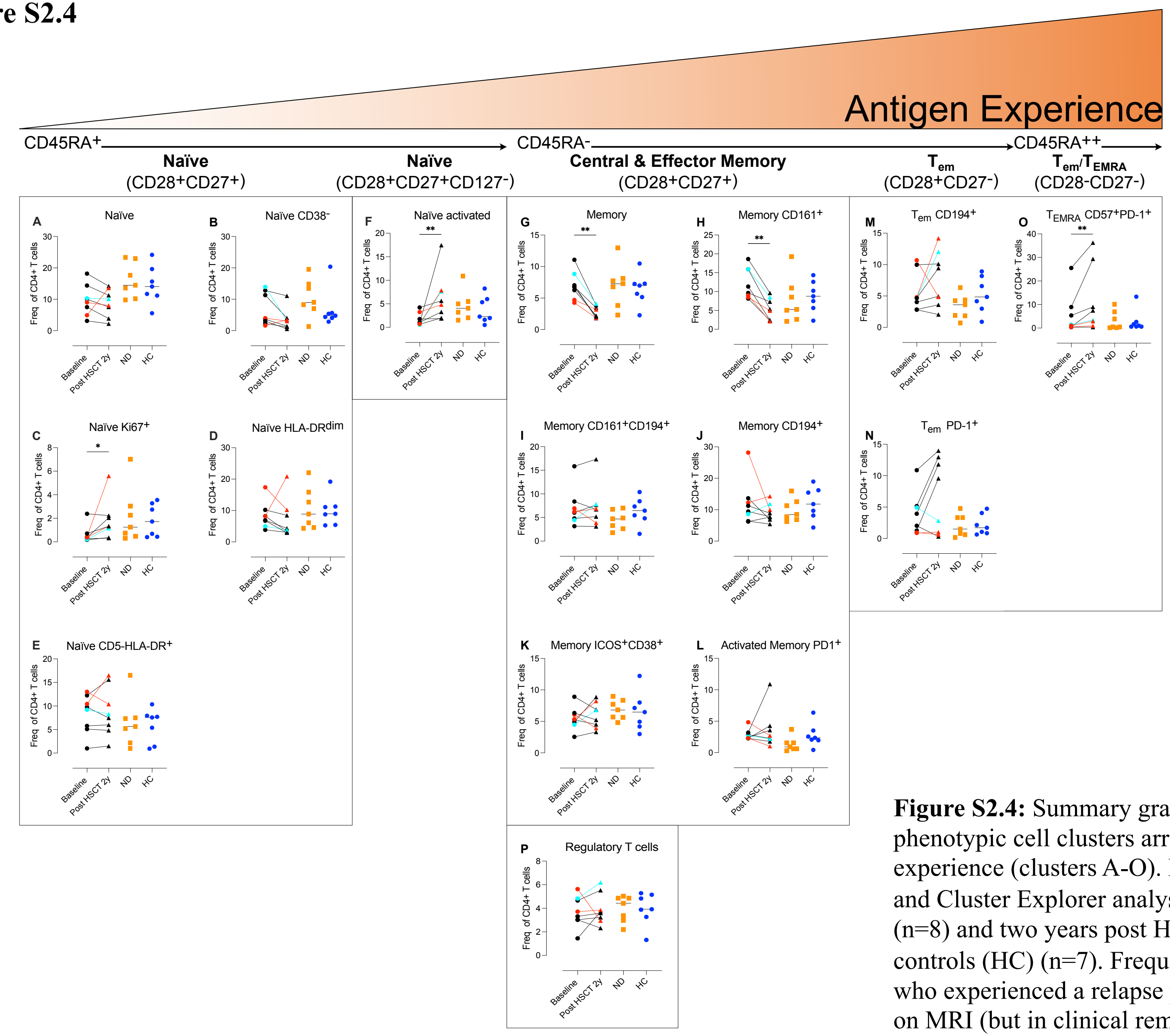

Figure S2.4: Summary graphs depicting the frequencies of CD4 T cells across distinct phenotypic cell clusters arranged from left to right in order of increasing maturation antigen experience (clusters A-O). Data is presented for each individual based on tSNE, PhenoGraph, and Cluster Explorer analysis of CyTOF data from **panel 2.** **Study** subjects: MS patients pre (n=8) and two years post HSCT (n=8), newly diagnosed MS patients (ND) (n=7), and healthy controls (HC) (n=7). Frequencies are expressed as a proportion of total CD4^{+} T cells. Patients who experienced a relapse post-HSCT are marked in **red**, while the patient with a new T2 event on MRI (but in clinical remission) is marked in **turquoise**. **Statistical analysis**: Wilcoxon matched-pairs test (*p<0,05. **p<0,01)

Figure S2.5

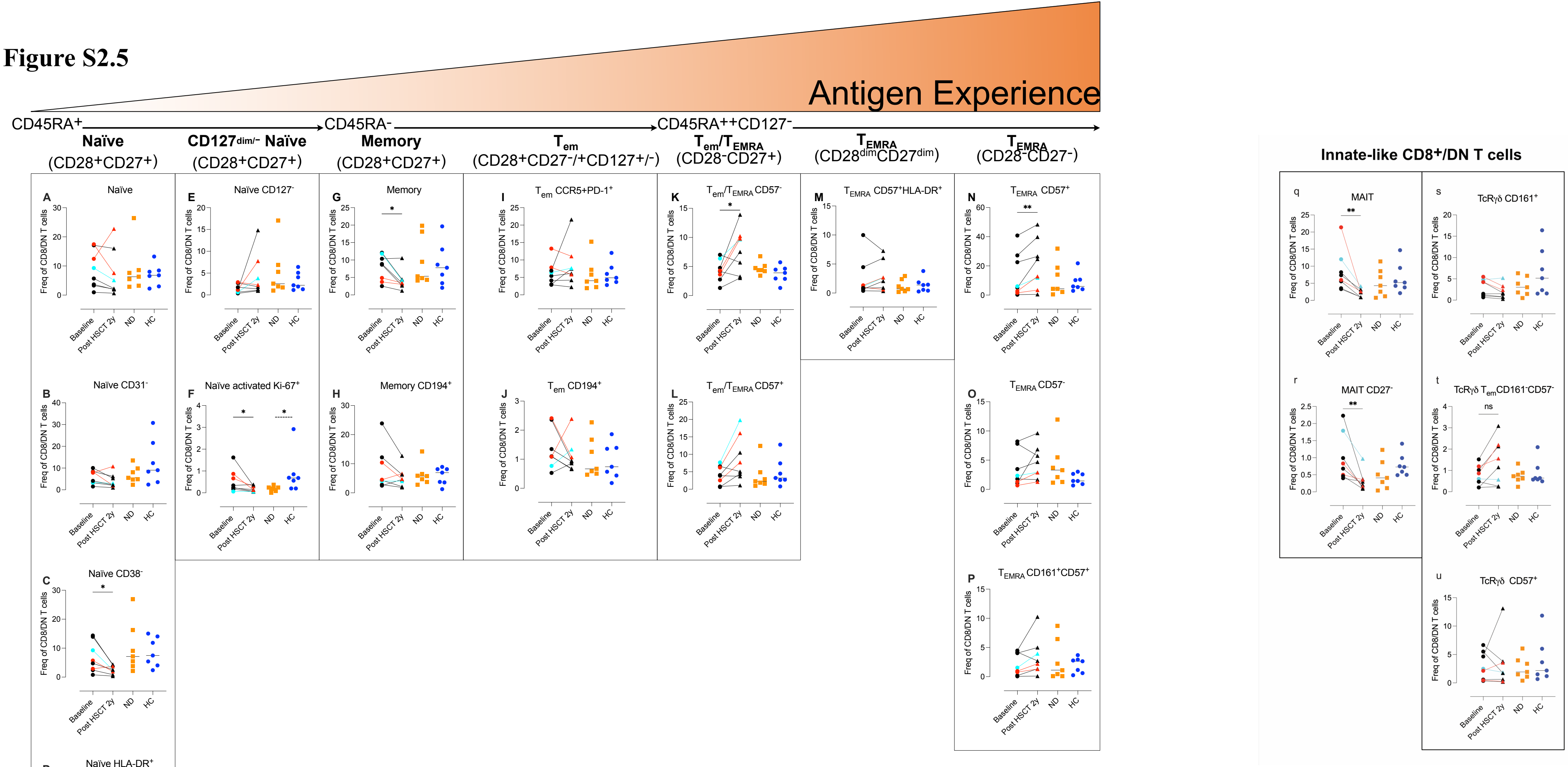

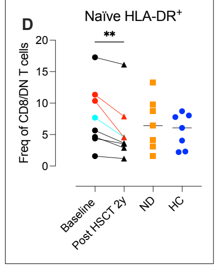

Figure S2.5: Summary graphs illustrating the frequencies of $CD8^+$ T cells, in each individual, across distinct phenotypic cell clusters, arranged from left to right in order of increasing maturation and antigen experience (clusters A–P), MAIT cells (clusters Q–R), and TcRγδ^+ T cells (clusters S-U). Data is presented for each individual based on tSNE, PhenoGraph, and Cluster Explorer analysis of CyTOF data from **panel 2.** **Study** subjects: MS patients pre (n=8) and two years post HSCT (n=8), newly diagnosed MS patients (ND) (n=7), and healthy controls (HC) (n=7). Frequencies are expressed as a proportion of total $CD8^+$ T cells. Patients who experienced a relapse post-HSCT are marked in **red**, while the patient with a new T2 event on MRI (but in clinical remission) is marked in **turquoise**. **Statistical analysis**: Wilcoxon matched-pairs test (*$p < 0.05$, **$p < 0.01$).

Figure S2.6

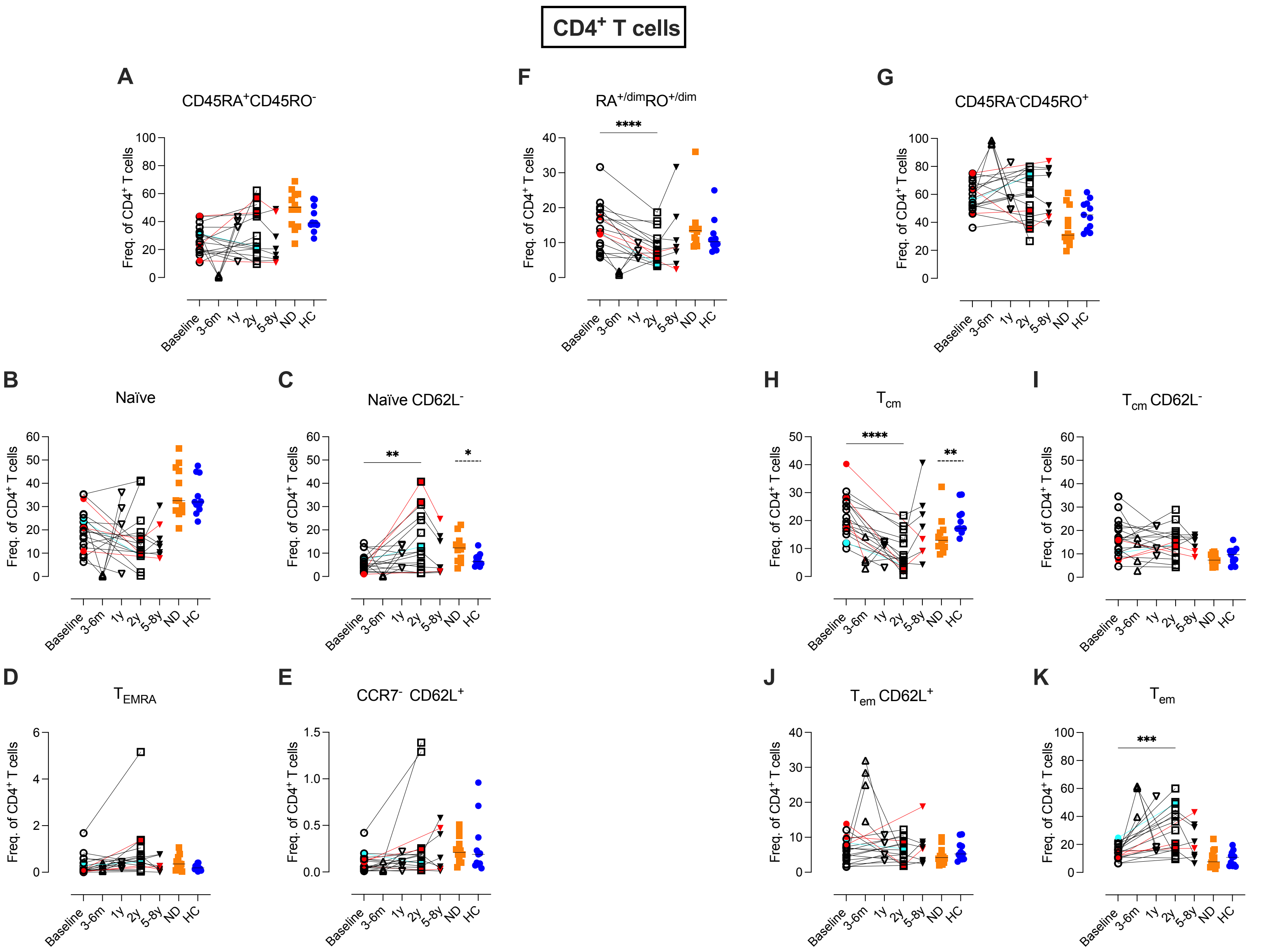

Figure S2.6: Flow cytometric analysis of maturation phenotype of CD4 T cells using CD45RA, CD45RO, CCR7 and CD62L as discriminating markers. (**A**) $CD45RA^{+}CD45RO^{-}$ (Naïve/T_{EMRA}), (**B**) Naïve ($CD45RA^{+}CD45RO^{-}CCR7^{+}CD62L^{+}$), (**C**) Naïve $CD62L^{-}$ ($CD45RA^{+}CD45RO^{-}CCR7^{+}CD62L^{-}$), (**D**) T_{EMRA} ($CD45RA^{+}CD45RO^{-}CCR7^{-}CD62L^{-}$), (**E**) $CCR7^{-}CD62L^{+}$ ($CD45RA^{+}CD45RO^{-}CCR7^{-}CD62L^{+}$), (**F**) $CD45RA^{+/dim}CD45RO^{+/dim}$ (transitional), (**G**) $CD45RA^{-}CD45RO^{+}$ (memory), (**H**) Central memory (T_{cm}) ($CD45RA^{-}CD45RO^{+}CCR7^{+}CD62L^{+}$), (**I**) T_{cm} $CD62L^{-}$ ($CD45RA^{-}CD45RO^{+}CCR7^{+}CD62L^{-}$), (**J**) Effector memory (T_{em}) ($CD45RA^{-}CD45RO^{+}CCR7^{-}CD62L^{-}$) and (**K**) T_{em} $CD62L^{+}$ ($CD45RA^{-}CD45RO^{+}CCR7^{-}CD62L^{+}$). Relative frequencies are expressed as a proportion of total $CD4^{+}$ T cells. Study subjects: MS patients at baseline (n=20), 4-6m (n=4), 1y (n=4), 2y (n=15), 5-8y (n=8) post HSCT, newly diagnosed MS patients (ND) (n=12) and healthy controls (HC) (n=11). Patients with relapse post HSCT is marked with red and the patient with new T2 event at 2 y but in clinical remission at that time point is marked with turquoise. Statistical analysis: Paired samples, Wilcoxon matched-paired test used (solid line, *p<0.05, **p<0.01, ***p<0.001, **** p<0.0001) for paired samples and Mann-Whitney test (hatched line, *p<0.05 and **p<0.01) for unpaired groups.

Figure S2.7

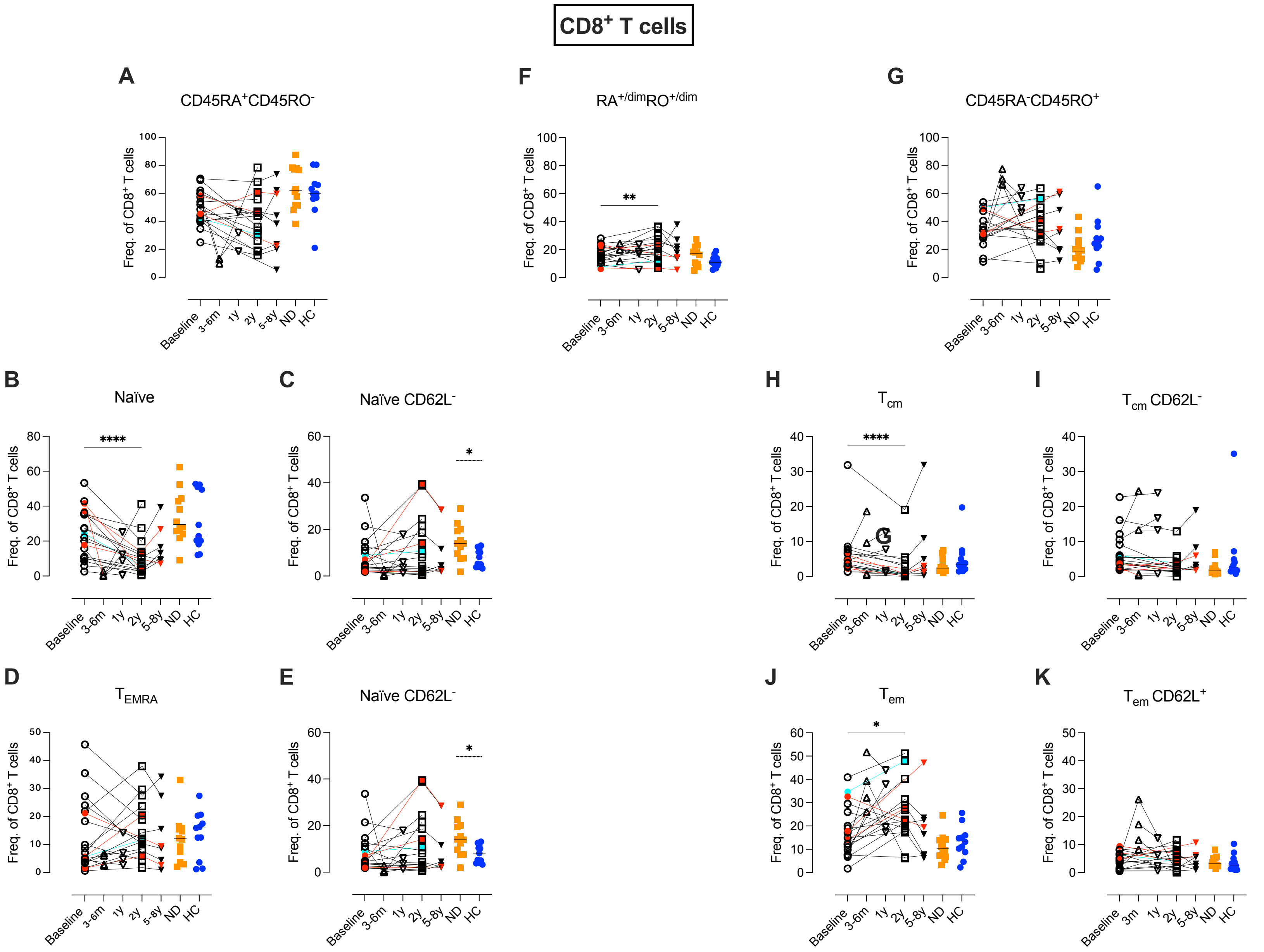

Figure S2.7: Flow cytometric analysis of maturation phenotype of CD8 T cells using CD45RA, CD45RO, CCR7 and CD62L as discriminating markers. (**A**) $CD45RA^{+}CD45RO^{-}$ (Naïve/T_{EMRA}), (**B**) Naïve ($CD45RA^{+}CD45RO^{-}CCR7^{+}CD62L^{+}$), (**C**) Naïve $CD62L^{-}$ ($CD45RA^{+}CD45RO^{-}CCR7^{+}CD62L^{-}$), (**D**) T_{EMRA} ($CD45RA^{+}CD45RO^{-}CCR7^{-}CD62L^{-}$), (**E**) $CCR7^{-}CD62L^{+}$ ($CD45RA^{+}CD45RO^{-}CCR7^{-}CD62L^{+}$), (**F**) $CD45RA^{+/dim}CD45RO^{+/dim}$ (transitional), (**G**) $CD45RA^{-}CD45RO^{+}$ (memory), (**H**) Central memory (T_{cm}) ($CD45RA^{-}CD45RO^{+}CCR7^{+}CD62L^{+}$), (**I**) T_{cm} $CD62L^{-}$ ($CD45RA^{-}CD45RO^{+}CCR7^{+}CD62L^{-}$), (**J**) Effector memory (T_{em}) ($CD45RA^{-}CD45RO^{+}CCR7^{-}CD62L^{-}$) and (**K**) T_{em} $CD62L^{+}$ ($CD45RA^{-}CD45RO^{+}CCR7^{-}CD62L^{+}$). Relative frequencies are expressed as a proportion of total $CD8^{+}$ T cells. Study subjects: MS patients at baseline (n=20), 4-6m (n=4), 1y (n=4), 2y (n=15), 5-8y (n=8) post HSCT, newly diagnosed MS patients (ND) (n=12) and healthy controls (HC) (n=11). Patients with relapse post HSCT is marked with red and the patient with new T2 event at 2 y but in clinical remission at that time point is marked with turquoise. Statistical analysis: Paired samples, Wilcoxon matched-paired test used (solid line, *p<0.05, **p<0.01, ***p<0.001, **** p<0.0001) for paired samples and Mann-Whitney test (hatched line, *p<0.05 and **p<0.01) for unpaired groups.

Figure S2.8

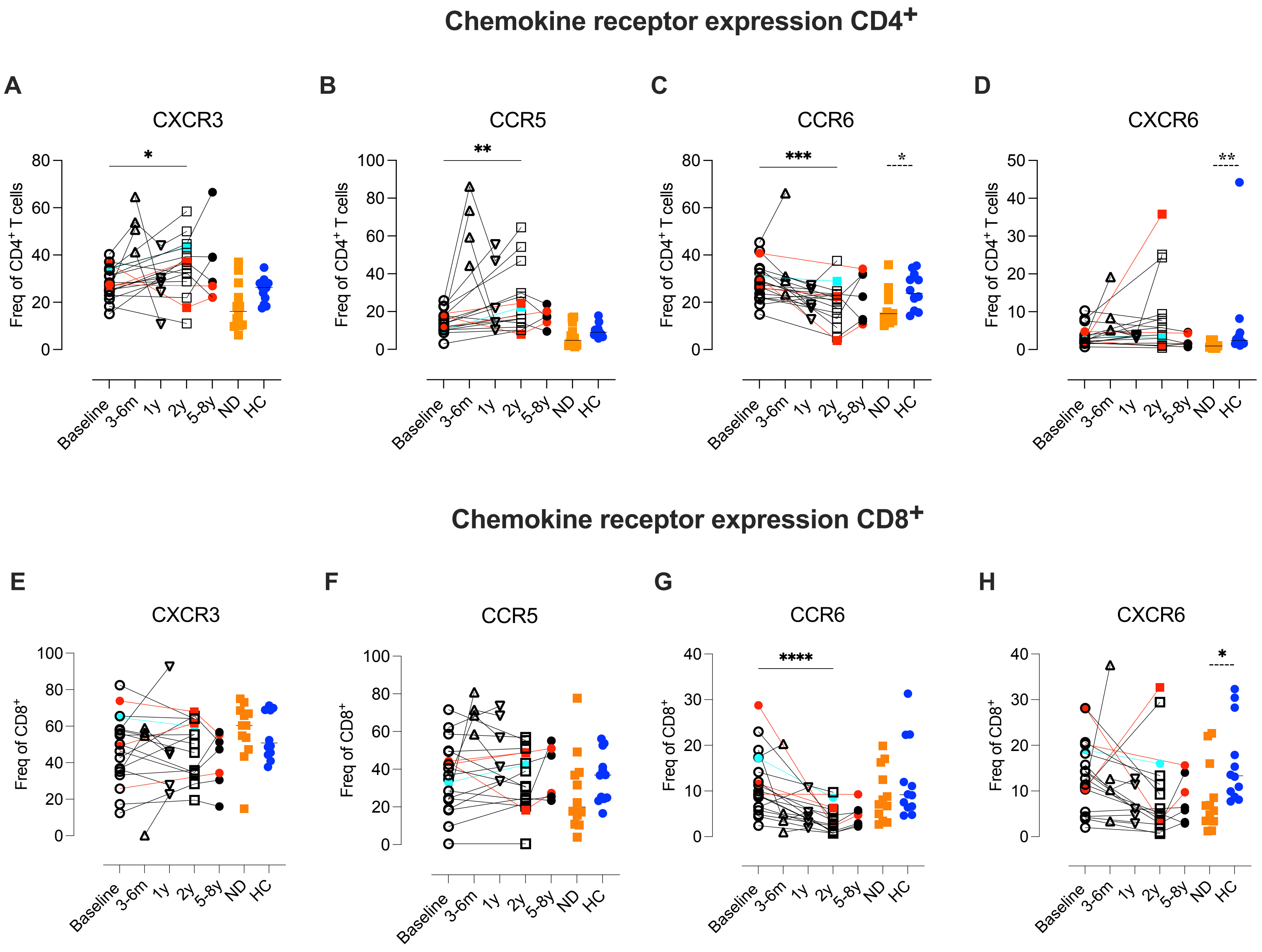

Figure S2.8: Summary graphs show flow cytometric analysis of the proportions of CD4$^+$ T cells (**A-D**) and CD8$^+$ T cells (**E-H**) positive for the expression of chemokine receptors CXCR3, CCR5, CCR6 and CXCR6. The frequencies shown are calculated out of total CD4$^+$ T cells and CD8$^+$ T cells respectively. Study subjects: MS patients at baseline (n=20), 4-6m (n=4), 1y (n=4), 2y (n=16), 5-8y (n=8) post HSCT, newly diagnosed MS patients (ND) (n=12) and healthy controls (HC) (n=11). Patients with relapse post HSCT is marked with red and the patient with new T2 event at 2 y but in clinical remission at that time point is marked with turquoise. Statistical analysis: Paired samples, Wilcoxon matched-paired test used (solid line, *p<0.05, **p<0.01, ***p<0.001, **** p<0.0001) for paired samples and Mann-Whitney test (hatched line, *p<0.05) for unpaired groups.

Figure S2.9

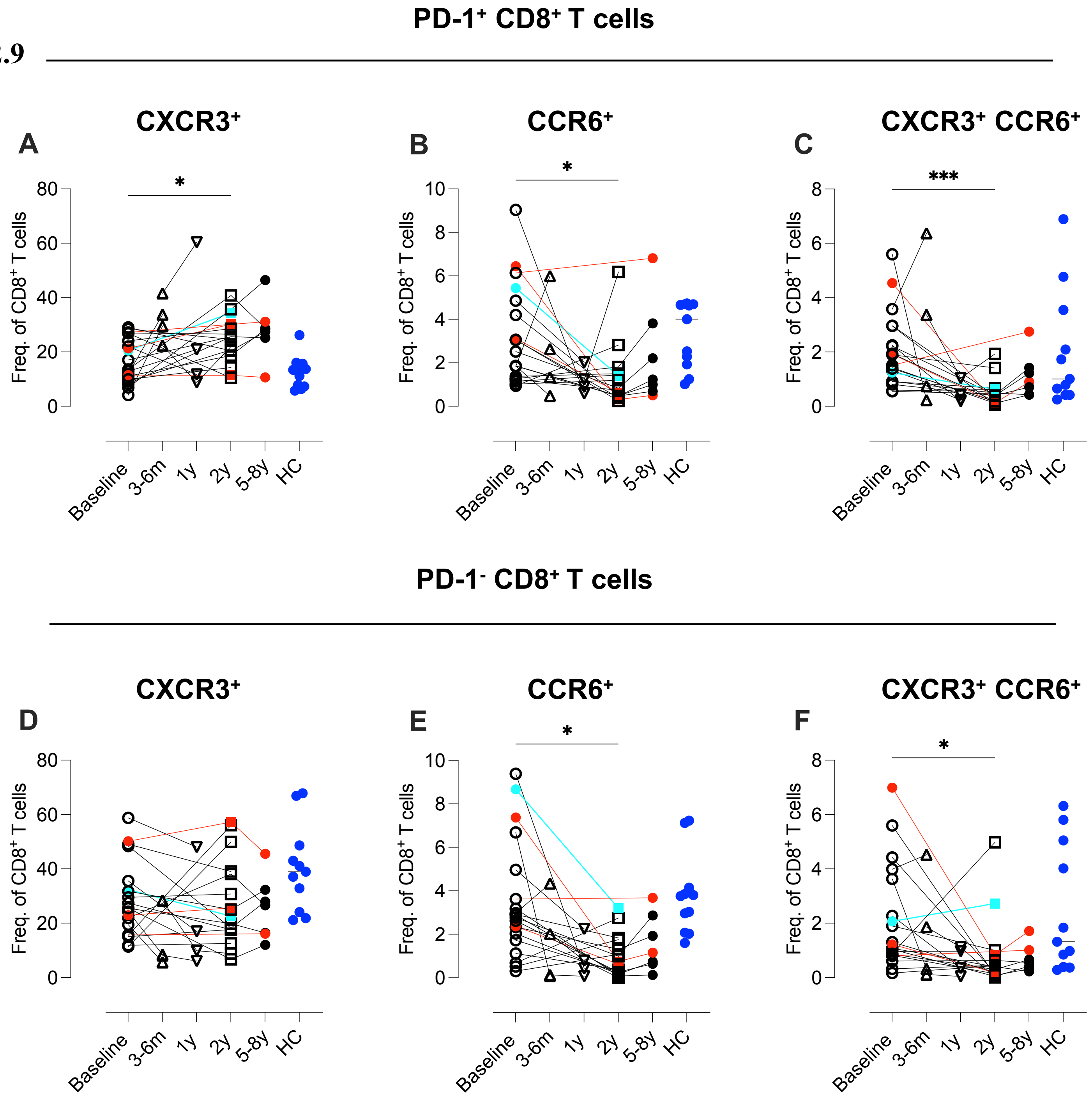

Figure S2.9: Flow cytometric analysis of co-expression of CXCR3, CCR6 and PD-1 on CD8 T cells. (**A**) PD-1^{+} CXCR3^{+} (CXCR3+CCR6^{-}), (**B**) PD-1^{+} CCR6 (CXCR3^{-} CCR6^{+}), (**C**) PD-1^{+} CXCR3^{+} CCR6^{+}) and (**D**) PD-1^{-} CXCR3^{+} (CXCR3+CCR6^{-}), (**E**) PD-1^{-} CCR6 (CXCR3^{-} CCR6^{+}), (**F**) PD-1^{-} CXCR3^{+} CCR6^{+}). The relative frequencies shown are calculated out of total CD8^{+} T cells. Study subjects: MS patients at baseline (n=20), 4-6m (n=4), 1y (n=4), 2y (n=16), 5-8y (n=7) post HSCT) and healthy controls (HC) (n=11). Patients with relapse post HSCT is marked with red and the patient with new T2 event at 2 y but in clinical remission at that time point is marked with turquoise. Statistical analysis: Paired samples, Wilcoxon matched-paired test used (solid line, *p<0.05, ***p<0.001).

Figure S2.10

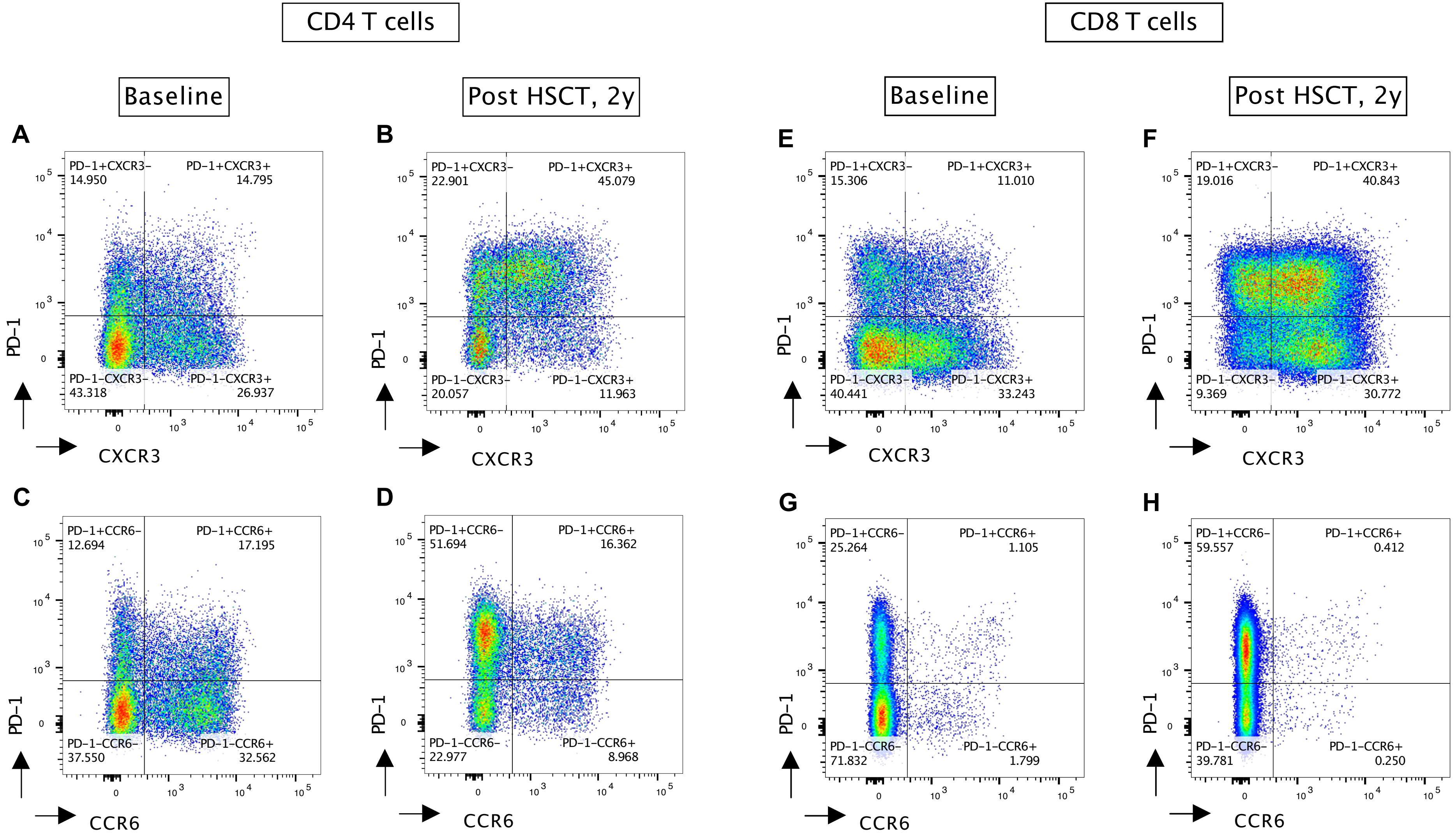

Figure S2.10: Representative dot plots of flow cytometric analysis of co-expression of PD-1/CXCR3 and PD-1/CCR6, at baseline and 2 years post AHSCT, in CD4 T cells (**A-D**) and in CD8 T cells (**E-H**). Cells depicted are from the same patient and gated on live $CD3^{+}$ T lymphocytes.

Figure S2.11

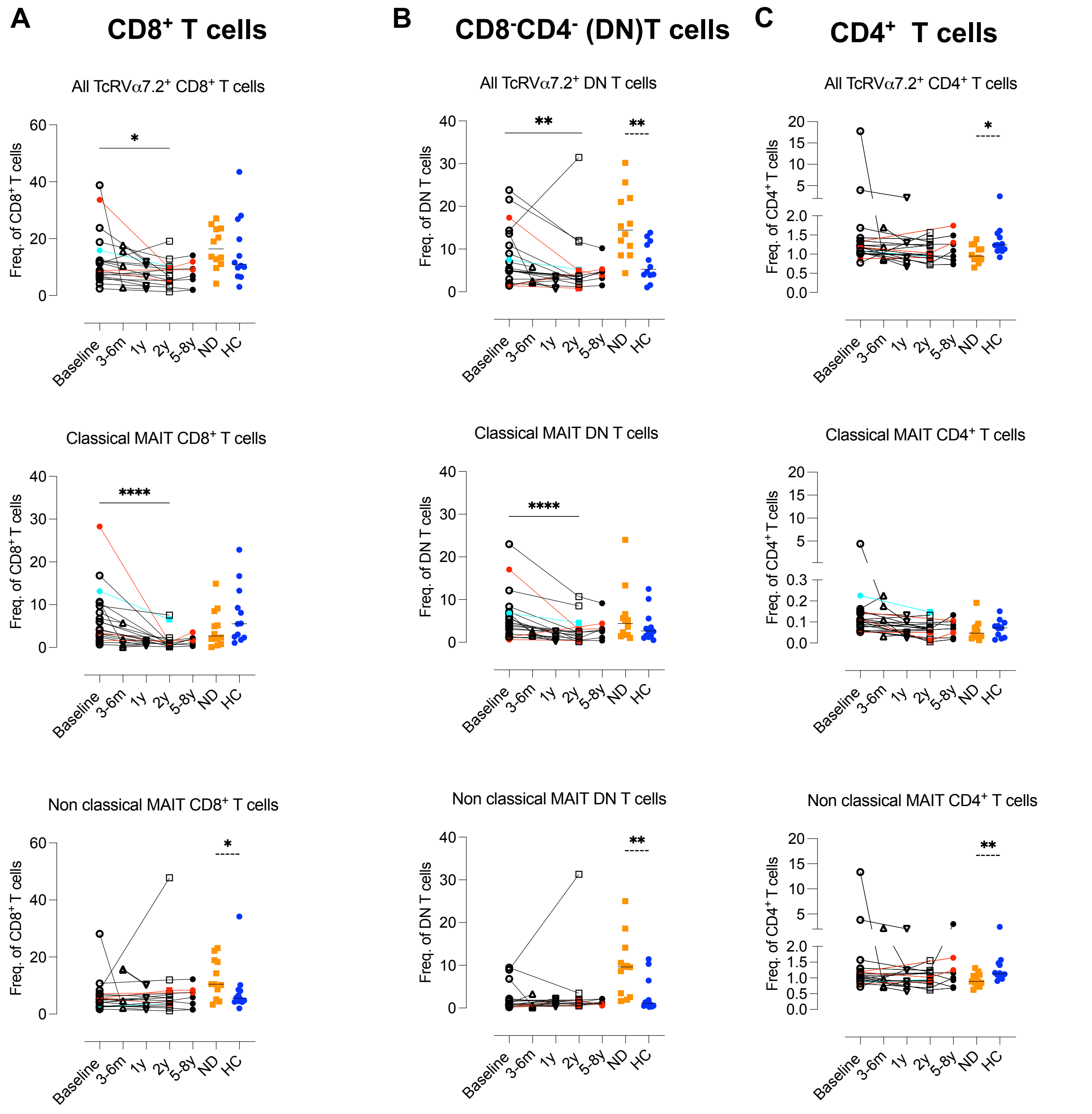

Figure S2.11: Summary graphs of flow cytometric analysis of all TcRα7.2 positive, $CD8^{+}$, DN and $CD4^{+}$ T cells or stratified for CD161 and IL-18R expression in those sub sets. Classical MAIT (TcRα7.2^{+} co-expressing CD161 and IL18R) and immature/non MAIT (TcRα7.2^{+} $CD161^{-/dim}$/ $IL18R^{-/dim}$ or $CD161^{-}$/ IL18R $^{+}$, $CD161^{+}$/ IL18R) in (**A**) CD8, (**B**) $CD8^{-}CD4^{-}$ (DN) and (**C**) $CD4^{+}$ T cells. The frequencies shown are calculated out of total $CD8^{+}$, DN and $CD4^{+}$ T cells respectively. Study subjects: MS patients at baseline (n=20), 4-6m (n=4), 1y (n=4), 2y (n=16), 5-8y (n=8) post HSCT, newly diagnosed MS patients (ND) (n=12) and healthy controls (HC) (n=11). Patients with relapse post HSCT is marked with red and the patient with new T2 event at 2 y but in clinical remission at that time point is marked with turquoise. Statistical analysis: Paired samples, Wilcoxon matched-paired test used (solid line, *p<0.05, **p<0.01, **** p<0.0001) for paired samples and Mann-Whitney test (hatched line, *p<0.05, **p<0.01) for unpaired groups.

Figure S2.12

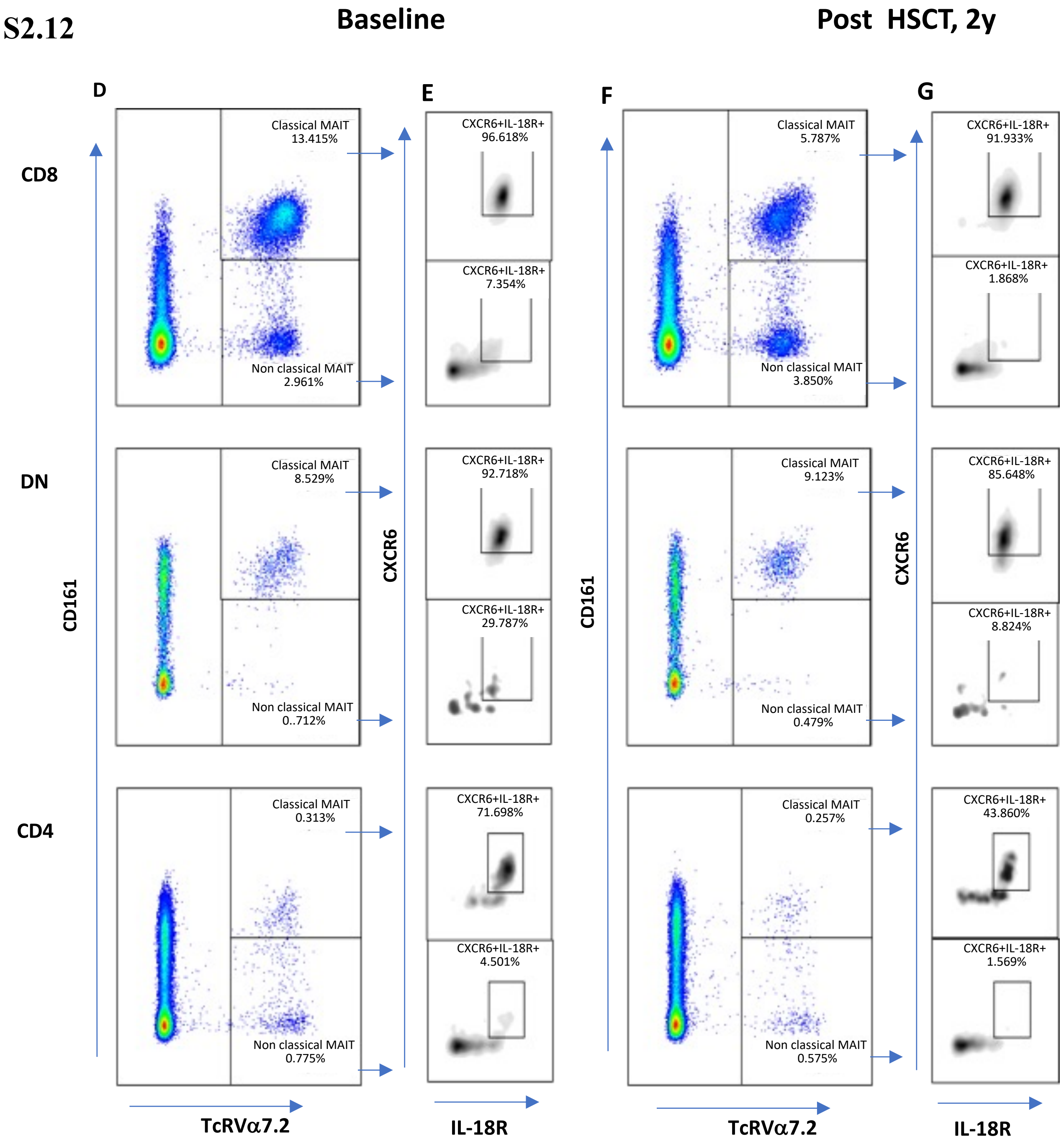

Figure S2.12: Representative dot plots of flow cytometric staining of TcRα7.2 and CD161 in $CD8^{+}$, DN and $CD4^{+}$ T cells, at baseline and 2y post ASCT, defining classical MAIT cells and immature/non MAIT cells as well as expression pattern of IL-18R and CXCR6 in these cell sub sets (**D-G**) Arrows indicate gating flow direction.

Figure S2.13

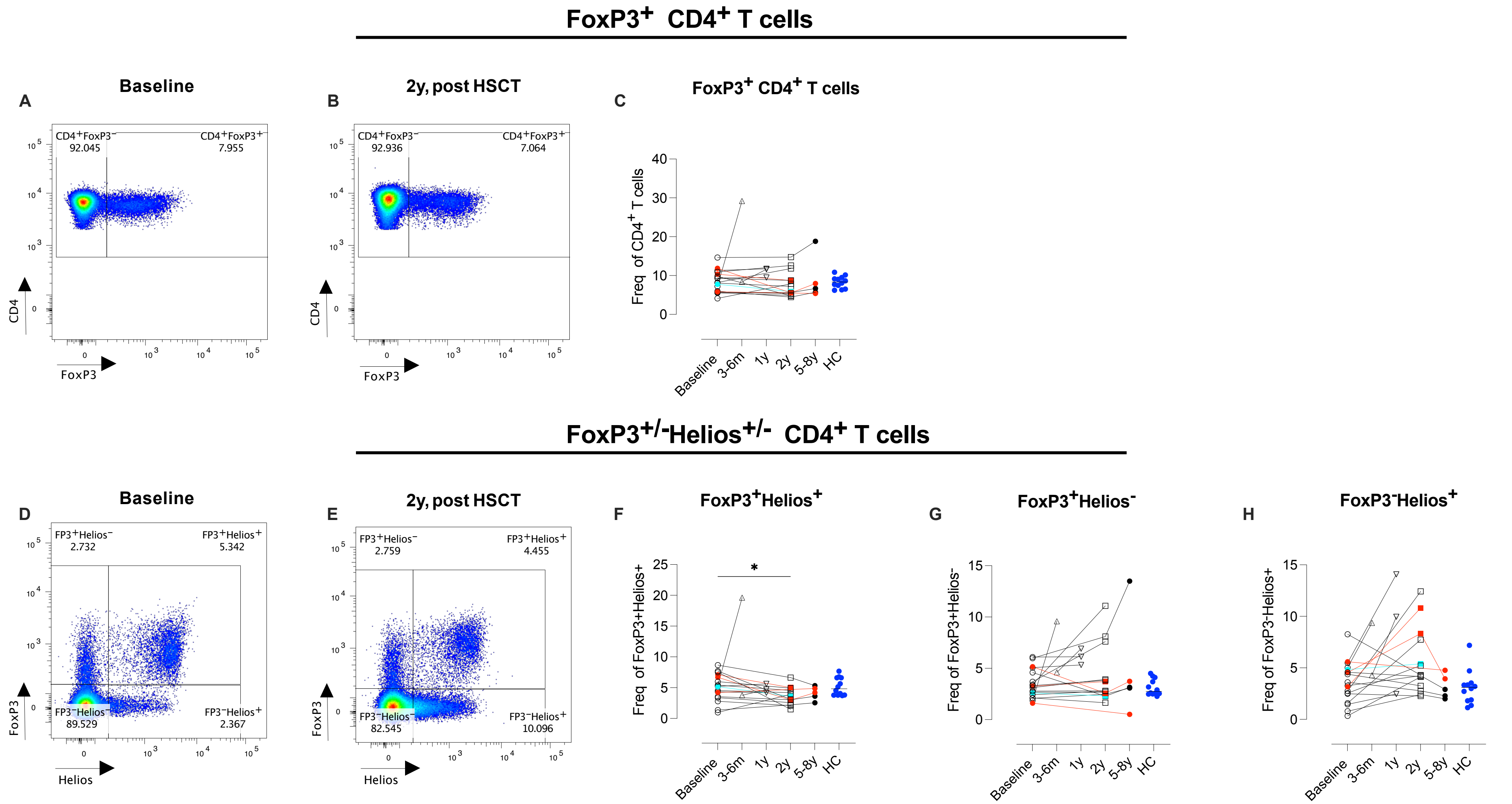

Figure S2.13: Representative dot plots and summary graphs of flow cytometric analysis of $CD4^+$ $FoxP3^+$ regulatory T cells (Tregs) in PBMCs, at baseline (n=16), 3-6m (n=2), 1y (n=3), 2y (n=13) and 5-8y post AHSCT (n=5) as well as in healthy subjects (HC) (n=12). Dot plots of $FoxP3^+$ staining of live $CD4^+$ T cells at Baseline (**A**) and 2 years post HSCT (**B**). Summary graph depicting relative frequencies of $FoxP3^+$ $CD4^+$ T cells at baseline and post AHSCT at different timepoints (**C**). Dot plots depicting staining patterns of Helios and FoxP3 in live $CD3^+CD4^+$ lymphocytes at baseline (**D**) and 2 years post AHSCT (**E**). Summary graphs showing proportions of $CD4^+$ T cells co-expressing FoxP3 and Helios (tTregs) (**F**), $CD4^+$ $FoxP3^+$ T cells negative for Helios (iTregs) (**G**) and non regulatory $CD4^+$ T cells negative for $FoxP3^+$ but positive for Helios (**H**).

Patients with relapse post HSCT is marked with red and the patient with new T2 event at 2 y but in clinical remission at that time point is marked with turquoise. Statistical analysis: Paired samples, Wilcoxon matched-paired test used (solid line, $*p<0.05$)

Figure S2.14

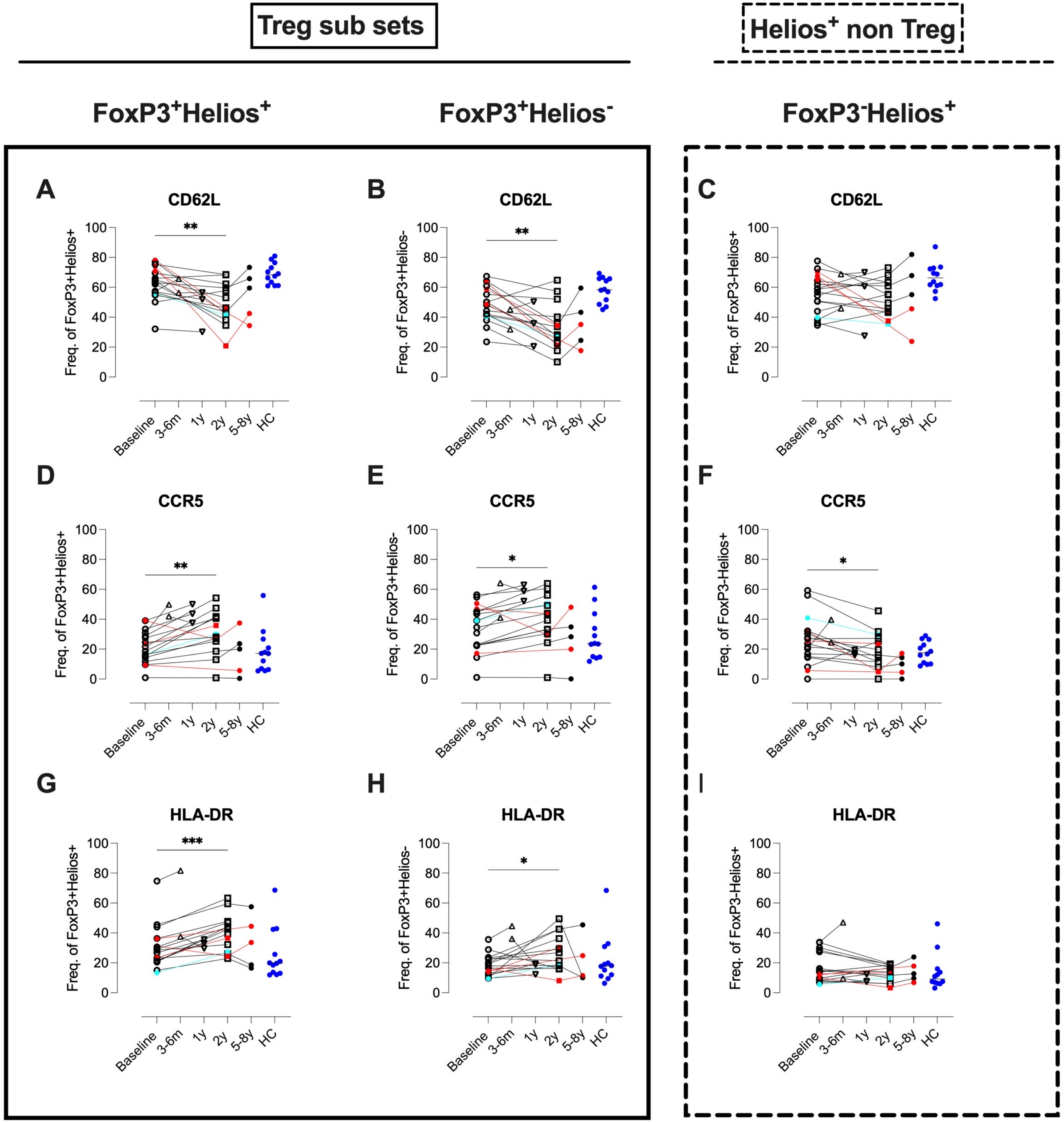

Figure S2.14: Summary graphs depicting the relative frequencies of the two Treg subsets and Helios+ non Treg CD4+ cells expressing CD62L, CCR5 and HLA-DR in PBMCs, at baseline (n=16), 3-6m (n=2), 1y (n=3), 2y (n=13) and 5-8y post AHSCT (n=5) as well as in healthy subjects (HC) (n=12). (**A**) CD62L+ FoxP3+Helios+ (tTregs), (**B**) CD62L+ FoxP3+Helios- (iTregs) and (**C**) CD62L+ Helios+ non Treg CD4+ T cells. (**D**) CCR5+ FoxP3+Helios+ (tTregs), (**E**) CCR5+ FoxP3+Helios- (iTregs) and (**F**) CCR5+ Helios+ non Treg CD4+ T cells. (**G**) HLA-DR+ FoxP3+Helios+ (tTregs), (**H**) HLA-DR+ FoxP3+Helios- (iTregs) and (**I**) HLA-DR+ Helios+ non Treg CD4+ T cells. Frequencies presented are out of respective cell sub set. Patients with relapse post HSCT is marked with red and the patient with new T2 event at 2 y but in clinical remission at that time point is marked with turquoise. Statistical analysis: Paired samples, Wilcoxon matched-paired test used (solid line, *p<0.05, **p<0.01 and ***p<0.001).
